# Supplementary material for: Improving mental health and physiological stress responses in mothers following traumatic childbirth and in their infants: study protocol for the Swiss TrAumatic biRth Trial (START)
Source: BMJ Open. 2019 Dec 30;9(12):e032469. doi: 10.1136/bmjopen-2019-032469 (PMC6955544; doi:10.1136/bmjopen-2019-032469)
Supplement: Author's manuscript [file bmjopen-2019-032469.draft_revisions.pdf]

BMJ Open is committed to open peer review. As part of this commitment we make the peer review history of every article we publish publicly available.

When an article is published we post the peer reviewers' comments and the authors' responses online. We also post the versions of the paper that were used during peer review. These are the versions that the peer review comments apply to.

The versions of the paper that follow are the versions that were submitted during the peer review process. They are not the versions of record or the final published versions. They should not be cited or distributed as the published version of this manuscript.

BMJ Open is an open access journal and the full, final, typeset and author-corrected version of record of the manuscript is available on our site with no access controls, subscription charges or pay-per-view fees (http://bmjopen.bmj.com).

If you have any questions on BMJ Open's open peer review process please email info.bmjopen@bmj.com

BMJ Open

Improving mental health and physiological stress responses in mothers following traumatic childbirth and in their infants: Study protocol for the Swiss TrAumatic biRth Trial (START)

Journal:	*BMJ Open*
Manuscript ID	bmjopen-2019-032469
Article Type:	Protocol
Date Submitted by the Author:	25-Jun-2019

Complete List of Authors:	Sandoz, Vania; University of Lausanne Faculty of Biology and Medicine, Institute of Higher Education and Research in Healthcare<br>Deforges, Camille; University of Lausanne Faculty of Biology and Medicine, Institute of Higher Education and Research in Healthcare<br>Stuijfzand, Suzannah; University of Lausanne Faculty of Biology and Medicine, Institute of Higher Education and Research in Healthcare<br>Epiney, Manuella; Geneva University Hospitals, Department Woman-Child-Adolescent<br>Vial, Yvan; University Hospital of Lausanne, 3Obstetrics and Gynecology Service, Woman-Mother-Child Department<br>Sekarski, Nicole; University Hospital of Lausanne, Paediatric Cardiology Unit, Woman-Mother-Child Department<br>Messerli, Nadine; University of Fribourg, Clinical Child Psychology & Biological Psychology<br>Ehlert, U; University of Zurich, Department of Clinical Psychology and Psychotherapy<br>Bickle-Graz, Myriam; University Hospital of Lausanne, Neonatology Service, Woman-Mother-Child Department<br>Morisod Harari, Mathilde; University Hospital of Lausanne, Service of Child and Adolescent Psychiatry,<br>Porcheret, Kate; Monash University, Turner Institute for Brain and Mental Health<br>Schechter, Daniel; NYU Langone Medical Center, Department of Child & Adolescent Psychiatry, ; University of Geneva Faculty of Medicine, Department of Psychiatry<br>Ayers, Susan; INTO City University London, Centre for Maternal and Child Health Research, School of Health Sciences<br>Holmes, Emily; Uppsala University, Department of Psychology<br>Horsch, Antje; University of Lausanne Faculty of Biology and Medicine, Institute of Higher Education and Research in Healthcare; University Hospital of Lausanne, Neonatology Service, Woman-Mother-Child Department,

Keywords:	early intervention, PTSD, maternal mental health, infant development, cortisol, HRV

SCHOLARONE™
Manuscripts

Improving mental health and physiological stress responses in mothers following traumatic childbirth and in their infants: Study protocol for the Swiss TrAumatic biRth Trial (START)

Vania Sandoz[1], Camille Deforges[1], Suzannah Stuijfzand[1], Manuella Epiney[2], Yvan Vial[3], Nicole Sekarski[4], Nadine Messerli, U Ehlert[6], Myriam Bickle-Graz[7], Mathilde Morisod Harari[8], Kate Porcheret[9], Daniel Schechter[10,11], Susan Ayers[12], Emily A. Holmes[13], & Antje Horsch[1,7] on behalf of the *START Research Consortium*

[1]Institute of Higher Education and Research in Healthcare (IUFRS), University of Lausanne, Lausanne, Switzerland

[2]Department Woman-Child-Adolescent, Geneva University Hospital and University of Geneva, Geneva, Switzerland.

[3]Obstetrics and Gynecology Service, Woman-Mother-Child Department, Lausanne University Hospital and University of Lausanne, Lausanne, Switzerland

[4]Paediatric Cardiology Unit, Woman-Mother-Child Department, Lausanne University Hospital and University of Lausanne, Lausanne, Switzerland

[5]Clinical Child Psychology & Biological Psychology, University of Fribourg, Fribourg, Switzerland

[6]Department of Clinical Psychology and Psychotherapy, University of Zurich, Zurich, Switzerland

[7]Neonatology Service, Woman-Mother-Child Department, Lausanne University Hospital and University of Lausanne, Lausanne, Switzerland

[8]Service of Child and Adolescent Psychiatry, Lausanne University Hospital and University of Lausanne, Lausanne, Switzerland

For peer review only - http://bmjopen.bmj.com/site/about/guidelines.xhtml

[9]Turner Institute for Brain and Mental Health, Monash University, Australia

[10]Department of Child & Adolescent Psychiatry, New York University Langone Medical School, New York, NY USA

[11]Department of Psychiatry, University of Geneva Faculty of Medicine, Geneva, Switzerland

[12]Centre for Maternal and Child Health Research, School of Health Sciences, City, University of London, London, UK

[13]Department of Psychology, Uppsala University, Uppsala, Sweden

Email addresses:

Vania.Sandoz@bluewin.ch

Camille.Deforges@chuv.ch

Suzannah.Ravenscroft@chuv.ch

Manuella.Epiney@hcuge.ch

Yvan.Vial@chuv.ch

Nicole.Sekarski@chuv.ch

Nadine.Messerli@unifr.ch

U.Ehlert@psychologie.uzh.ch

Myriam.Bickle-Graz@chuv.ch

Mathilde.Morisod@chuv.ch

Kate.Porcheret@monash.edu

Daniel.Schechter@hcuge.ch

Susan.Ayers.1@city.ac.uk

Emily.Holmes@ki.se

Antje.Horsch@chuv.ch

For peer review only

Corresponding author:

Prof. Antje Horsch, Institute of Higher Education and Research in Healthcare (IUFRS), University of Lausanne, SV-A, Route de la Corniche 10, 1010 Lausanne. Phone: 00 41 21 314 85 71. Antje.Horsch@chuv.ch

KEYWORDS

early intervention, PTSD, maternal mental health, randomised controlled trial, infant development, HRV, cortisol, sleep, father, Bayley-III

WORD COUNT

4319 words, excluding abstract, acknowledgements, author contributions, and references

For peer review only - http://bmjopen.bmj.com/site/about/guidelines.xhtml

ABSTRACT

Introduction

Emergency caesarean section (ECS) qualifies as a psychological trauma, which may result in postnatal posttraumatic stress disorder (PTSD). Maternal PTSD may not only have a significant negative impact on mother-infant interactions, but also on long-term infant development. While the impact of ECS on partners is understudied, the partner's mental health may also affect infant development. Evidence-based early interventions to prevent the development of postpartum PTSD are lacking. Immediately after a traumatic event, memory formation is vulnerable to interference. There is accumulating evidence that a brief behavioural intervention including a visuospatial task may result in a reduction in intrusive memories.

Methods and analysis

This study protocol describes a double-blind multi-centre randomised controlled phase III trial testing an early brief intervention including the computer game "Tetris" on intrusive memories (1 week) and PTSD symptoms (6 weeks) of 144 women following an ECS. The intervention group will carry out a brief behavioural procedure including playing Tetris. The attention-placebo control group will complete a brief written activity log. Both simple cognitive tasks will be completed within the first 6 hours following childbirth. The intervention is delivered by midwives/nurses in the maternity unit.

The primary outcome will be differences in the presence and severity of maternal PTSD symptoms between the intervention and the attention-placebo control group at 6 weeks postpartum. Secondary outcomes will be physiological stress and psychological vulnerability, mother-infant interaction, and infant developmental outcomes. Other outcomes will be

For peer review only - http://bmjopen.bmj.com/site/about/guidelines.xhtml

psychological vulnerability and physiological regulation of the partner and their bonding with the infant.

Ethics and dissemination

Ethical approval was granted by the Human Research Ethics Committee of the Canton de Vaud (study number 2017-02142). Dissemination of results will occur via national and international conferences, in peer-reviewed journals, public conferences, and social media.

Trial registration number

clinicaltrials.gov (NCT 03576586)

ARTICLE SUMMARY

Strengths and limitations of this study

- The multi-centre RCT will test the effects of an early brief intervention and is carried out by midwives/nurses on the labour ward.
- Among other objectives, the intervention aims at preventing PTSD, including intrusive traumatic memories, following traumatic childbirth and reducing its impact on maternal and child physiology, child development, the partner, and the couple relationship.
- The early brief intervention, conducted in the same hospital where the traumatic event occurred, includes a visuospatial task aimed at competing with sensory aspects of the traumatic memory before it has been fully consolidated.
- Methodological rigour, including a double-blind design, an active control group, concealment of random allocation, regular monitoring, and prospective trial registration and publication, limits risk of bias.

For peer review only - http://bmjopen.bmj.com/site/about/guidelines.xhtml

ABBREVIATIONS

ANS: Autonomic Nervous System / ASD: Acute Stress Disorder / ASDS: Acute Stress Disorder Scale / BSID-III: Bayley Scales of Infant Development, 3rd Edition / CAPS: Clinician-Administered PTSD Scale for DSM-5 / CAR: Cortisol Awakening Response / CRIB: Clinical Risk Index for Babies / EAS: Emotional Availability Scales / ECG: Electrocardiography / ECS: Emergency Caesarean Section / EPDS: Edinburgh Postnatal Depression Scale / FFSF: Face-to-Face Still-Face paradigm / HADS: Hospital Anxiety and Depression Scale / HPA: Hypothalamic-Pituitary-Adrenal / HRV: Heart Rate Variability / IBIs: Interbeat Intervals / IBQ-R: Infant Behavior Questionnaire-Revised / MEQ: Morningness-Eveningness Questionnaire / MIBS: Mother-to-Infant Bonding Scale / MOS-8: Modified Medical Outcomes Study Social Support Survey / NBAS: Neonatal Behavioural Assessment Scale / PCL-5: PTSD Checklist for DSM-5 / PDPSI: Post-delivery Perceived Stress Inventory / PPSI: Postnatal Perceived Stress Inventory / PSI-SF: Parenting Stress Index - Short Form / PSQI: Pittsburgh Sleep Quality Index / PSQI-A: Pittsburgh Sleep Quality Index Addendum / PTSD: Post-Traumatic Stress Disorder / RCT: Randomised Controlled Trial / RDAS: Revised Dyadic Adjustment Scale / START: Swiss Traumatic Birth Trial

For peer review only

For peer review only - http://bmjopen.bmj.com/site/about/guidelines.xhtml

INTRODUCTION

Childbirth and PTSD

Though childbirth is a common and often fulfilling event, one third of mothers rate their childbirth as traumatic[1]. Childbirth can meet diagnostic criteria for a traumatic event, if women perceived their life and/or the life of their baby to be in danger[2]. Posttraumatic stress disorder (PTSD) related to childbirth is diagnosable in around 3-4% of women[3 4]. PTSD consists of four symptom clusters (intrusions, avoidance, hyperarousal, and negative cognitions and mood) and can be diagnosed at least one month after the traumatic stressor occurred[2]. Comparing different modes of childbirth, obstetric complications, such as emergency caesarean section (ECS) produce higher rates of postnatal PTSD (19-39%)[3]. ECS is a relatively frequent event, and there is thus a need to better identify and support women who are vulnerable to developing PTSD following an ECS.

Postnatal PTSD can significantly influence the experience of subsequent pregnancies, with increased risk of maternal stress and its associated risks of intrauterine growth retardation, premature birth and low birth weight[5-7]. It can lead to a fear of subsequent pregnancy and childbirth, sexual problems, and avoidance of medical care[8 9]. Postnatal PTSD can also have important negative consequences for breastfeeding, the attachment relationship with the baby and mother-infant interactions, with a subsequent detrimental impact on the development of the child, as well as for the couple relationship[7 10-12]. PTSD is also highly co-morbid with depression, for which there is substantial evidence of long-term negative effects on child development and behaviour[13 14]. Estimated economic costs of perinatal mental health problems are about £8.1 billion for each one-year cohort of UK births, of which 72% relate to adverse impacts on the child rather than the mother[15]. In Switzerland, 16.7% of women in the perinatal period used mental health services[16]. New and innovative evidence-based

For peer review only - http://bmjopen.bmj.com/site/about/guidelines.xhtml

interventions are therefore needed to reduce those costs by preventing the development of postnatal PTSD[17].

To date, research investigating PTSD symptoms in partners following ECS is missing. Most studies on fathers so far have focused on postnatal depression reporting that 1-8% experience depression symptoms in the first 6 weeks and 5-6% at 3-6 months following childbirth without complications[18-20], with increased risk following high risk situations[21]. In one study, 5% of fathers reported severe intrusions and avoidance symptoms at 9 weeks postpartum[22]. Although the influence of partner mental health is understudied, it also seems to negatively impact child outcomes[23-25]. Thus, partner mental health needs to be better understood not only in order to help the partner but also to support family and child outcomes.

Physiological stress responses associated with PTSD

Traumatic exposure activates the hypothalamic-pituitary-adrenal (HPA) axis, a cascade-like hormonal system resulting in the release of cortisol from adrenal cortex cells in body fluids. In parallel, the organism activates the more rapidly mobilizing autonomic nervous system (ANS) resulting in the release of norepinephrine from nerve terminals of the sympathetic nervous system as well as epinephrine and norepinephrine from the adrenal medulla[26]. While the HPA axis shows the above stress-related reactivity, it also shows a basal activity with circadian variations in the respective hormones. For instance, cortisol peaks 30-45 minutes after awakening (the so-called CAR: cortisol awakening response) and gradually declines throughout the day with lowest levels early during sleep[27 28].

Specific patterns of HPA axis functioning have been shown in PTSD[29] although this has so far not been studied after traumatic childbirth. While studies indicate that individuals with PTSD show different patterns of HPA axis functioning to those without PTSD, there is little consistency in the specificity of these patterns[30-36]. A meta-analysis examining diurnal

For peer review only - http://bmjopen.bmj.com/site/about/guidelines.xhtml

cortisol levels in adults with PTSD showed that low cortisol levels were not related to PTSD in general, but rather to trauma exposure and co-morbidities[37]. Finally, a recent study in a postnatal population found a negative association between symptoms of re-experiencing and diurnal cortisol slopes in mothers of preterm children[38]. Concerning cortisol reactivity to a subsequent stressor, again, results are discrepant[39 40].

Although HPA axis reactivity following stress or trauma is thought to be adaptive, acute or chronic exposure to stress has been shown to have deleterious effects[41 42]. It may not only result in dysfunctions of HPA re-activity, but also in health-relevant changes in the basal activation of this system[43]. Overall, these studies show the strong implication of the HPA axis dysregulation in the development and maintenance of PTSD, although studies in postnatal populations are scarce.

Reduced heart rate variability (HRV), an indicator of autonomic flexibility, has been found to be related to psychopathological processes[44]. Individuals with PTSD show lower levels of HRV in comparison with trauma-exposed individuals without PTSD or healthy controls[45 46]. However, the relationship of PTSD and HRV has so far not been studied in a postnatal population.

Sleep in PTSD is also disrupted. Sleep disturbance (i.e., difficulty falling or staying asleep) and recurrent distressing dreams are both diagnostic criteria for PTSD[47], with 70-91% of patients with PTSD suffering from subjective sleep disturbances and 19-71% reporting nightmares[48]. Findings from experimental research indicate that sleep on the first night after trauma may be important for the development of subsequent intrusive memories. One study found that the sleep deprived participants reported *fewer* intrusive memories compared to those who slept[49], though findings are mixed[50-52]. Thus, it is important to assess sleep over time following a real world traumatic event, such as following an ECS. To date, no studies to our knowledge have examined sleep in postpartum PTSD.

For peer review only - http://bmjopen.bmj.com/site/about/guidelines.xhtml

Maternal PTSD and infant physiological stress responses

Maternal PTSD and its associated dysfunction of the HPA axis can also impact on the stress regulation of the offspring[53-63], such as the infant's HPA secretion patterns[64-67]. A growing body of neuroendocrine research supports the notion that an altered maternal HPA axis functioning plays a role in the intergenerational transmission of stress-related psychopathology from parents[68 69]. Overall, findings suggest that PTSD symptoms and cortisol levels in mothers are important to assess, prevent and/or treat as they may affect the relationship with the infant[70-73] and impact the child's later regulative abilities[53]. Some authors have suggested low maternal cortisol as a possible mechanism contributing to the mother's difficulty in sensitively attuning to her infant's cues, which in turn impacts on the infant's reactivity to and recovery from a stressor[74-79].

In contrast, studies assessing the role of ANS in the intergenerational transmission of stress in the postpartum period are so far scarce. Lifetime maternal psychopathology and maternal postnatal psychopathology have been found to be related with reduced HRV of their infants[80]. Furthermore, mothers with anxiety symptoms during pregnancy and their infants showed lower HRV[81] and there was a higher sympathetic activation in children of mothers with abuse histories[82]. However, to our knowledge none of the previous research has investigated the autonomic functioning in offspring of mothers with PTSD.

Developing an early intervention inspired by cognitive neuroscience

To date, there is a lack of evidence-based early interventions for women following a traumatic childbirth[83]. At the heart of PTSD are intrusive memories of the traumatic event, in which the person re-experiences aspects of the traumatic event, inflicting significant distress[2]. They have also been indicated as a precursor to the disorder[84]. Intrusive memories of trauma

For peer review only - http://bmjopen.bmj.com/site/about/guidelines.xhtml

comprise sensory-perceptual images[85] that are proposed to occur due to excessive perceptual (sensory) processing during a trauma[86].

The way in which individuals process a traumatic event influences their later intrusive memories. Evidence from lab-based experiments have demonstrated that a brief behavioural intervention including a reminder cue and a visuospatial task (Tetris) can significantly reduce the frequency of intrusive traumatic images following exposure to traumatic film material[87 88]. One study showed that individuals who were instructed to engage in conceptually-driven processing, relative to those engaged in sensory-based, data-driven processing, reported more intrusive traumatic memories to a traumatic film[89]. It has been hypothesised that tasks which interfere with data-driven processing, such as sensory-perceptual, visuospatial tasks, may reduce the occurrence of intrusive memories[90]. Visuospatial cognitive tasks, such as the computer game Tetris, have been shown to compete for resources with visuospatial images[91]. Studies of memory consolidation have shown that memories are malleable within 10 min to 6 hours, at which point the memory is thought to undergo the first stabilization phase of consolidation, making it resistant to interference from a competing memory[92]. This indicates that such a visuospatial working memory task needs to take place within approximately the first 6 hours following a traumatic event (see[90] for a review).

Two recent translational studies presented preliminary evidence for the efficacy of a brief intervention (including Tetris) in reducing the number of intrusive traumatic memories (over 1 week post-trauma) in patients arriving at an accident and emergency department[93] or in women following ECS, compared with a treatment-as-usual control group[4]. In the latter study, per protocol analyses also showed significantly lower re-experiencing symptoms at 1 week and lower rates of PTSD diagnosis at 1 month following ECS (secondary outcomes)[4].

Aims of the present study

For peer review only - http://bmjopen.bmj.com/site/about/guidelines.xhtml

The objectives of the present study are to measure the effects of a brief computerized intervention (Tetris) on maternal mental health and infant development after a traumatic event (ECS). The primary outcome measure will be the presence and the severity of maternal PTSD symptoms at 6 weeks. Secondary objective will be to measure the impact of this intervention on intrusive traumatic memories, on stress exposure and perception, on other indicators of maternal psychological vulnerability (including acute stress disorder (ASD), PTSD, anxiety, depression, and sleep), on physiological stress reactivity, on physiological regulation, on mother-infant interactions, and on infant development. The START study also aims to investigate additional maternal and partner psychological vulnerability, partner physiological regulation, partner-infant bonding, and measures related to the acceptability and expectancy of the intervention.

METHODS

Study Design

We will conduct a multi-centre double-blind randomised controlled trial (RCT) with minimal risk testing the effect of an early brief behavioural intervention including computer game play for women at risk of PTSD following an ECS and their infant, compared with an attention-placebo control task.

Study population, recruitment, group allocation, and blinding

All women who have an ECS ≥34 weeks gestation, give birth to a live baby, and give written consent are eligible to participate. In addition, they have to answer with a score of ≥2 separately for at least two out of four screening questions regarding perceived threat[94]. All screening questions are answered on a 7-point Likert scale (*1 = not at all, 7 = extremely*): Did

For peer review only - http://bmjopen.bmj.com/site/about/guidelines.xhtml

you think that your life was in danger? Did you think that your baby's life was in danger? Did you feel frightened during the birth? Did you feel helpless during the birth?

Exclusion criteria include: established intellectual disability or psychotic illness, insufficient French-speaking level to participate in assessments, severe illness of mother or infant (e.g., cancer, cardiovascular disease, severe neurodevelopmental difficulties, malformations) or if the infant requires intensive care, and alcohol abuse and/or illegal drug use during pregnancy.

Following an ECS and once the mother has sufficiently recovered, the mother's midwife/nurse will assess eligibility and if eligible, will inform the mother about the study. After providing written and informed consent to be screened, participants will be screened immediately for perceived threat of the mother and/or child with four screening questions. If participants score ≥2 separately for at least two out of the four questions, they will be randomly assigned to either the intervention or attention-placebo control group. We aim to recruit 144 women and their infants (see sample size calculation).

If the woman agrees, then the partner will also be informed about the study. Inclusion criteria for the partners are that they were present at the childbirth and give written consent. Partners are excluded if they do not speak French sufficiently well to participate in assessments. All participants will be reimbursed for their time and effort.

The allocation ratio of randomisation is 1:1. The randomization sequence will be generated using a computer-generated block randomisation (StataCorp. 2017. *Stata Statistical Software: Release 15*. College Station, TX) using blocks of sizes 2, 4 and 6 over 144 participants per stratum (stratified by research centre). Opaque envelopes will be prepared in advance, numbered sequentially by alternating between stratums by block. After conducting the baseline assessment, the clinical midwife/nurse will open the envelope and announce the group allocation to the participant[95]. All members of the research group as well as participants

For peer review only - http://bmjopen.bmj.com/site/about/guidelines.xhtml

are blind to group allocation. All participant data will be coded to ensure confidentiality. After completing the 15-minute cognitive tasks, both women of the control and intervention group will complete the same assessments, as shown in Table 1; see Figure 1 for an overview of the study variables.

Intervention group

Mothers in the intervention group will be instructed to engage in a cognitive visuospatial task, the computer game Tetris, for 15 minutes continuously, on a handheld gaming device (Nintendo DS) (see[4]). They are given a 3-min training in how to play the game and how to actively use mental rotation as they played the game. The intervention is delivered in the same context as that in which the trauma occurred (e.g, wake-up room) so additional memory reminder cue to the trauma is not used.

The intervention will take place within the first six hours after ECS. Procedures are managed not to interfere with important routine care.

Attention-placebo control group

Mothers assigned to the control group will be asked to engage in a written activity log for 15 minutes. The activity log was selected to control for as many nonspecific confounding factors as possible, while minimizing the potential for harmful effects, as to date, no preventive treatment in the immediate aftermath of trauma exists that could be used as a control condition[96-99]. This control condition was matched with the intervention condition for nonspecific factors including length of the task, contact with the midwife/nurse, location of treatment procedure and engagement in a structured task[93]. Study information and materials refer to 'simple tasks' in both conditions for credibility. The attention-placebo control task will

For peer review only

For peer review only - http://bmjopen.bmj.com/site/about/guidelines.xhtml

take place within the six hours after ECS, whilst participants are still in their hospital bed and will not interfere with important routine care procedures[88 100].

Primary outcome

The primary outcomes are differences in the presence and severity of maternal PTSD symptoms between the intervention and the attention-placebo control group at 6 weeks postpartum measured with the Clinician-administered PTSD Scale and the PTSD Checklist.

Secondary outcomes

The following outcomes will be assessed via validated assessments at different time-points (i.e., ≥6 hours following ECS, ≤1 and 6 weeks, and 6 months follow-up).

Maternal outcomes

The maternal mental health outcomes will be compared between the two groups at all time-points, including number of intrusive traumatic memories and indicators of maternal psychological vulnerability namely: symptoms of ASD, PTSD, anxiety, depression, sleep and physical activity (see Table 1). Additional physiological outcomes will be collected in reactivity to stress and as regulation indicators. Finally, maternal bonding and sensitivity in mother-infant interaction will also be measured.

Child outcomes

As shown in Table 1, infant development will be assessed at 6 months postpartum. Additionally, physiological outcomes will be assessed in response to stress and as regulation indicators.

For peer review only - http://bmjopen.bmj.com/site/about/guidelines.xhtml

Other outcomes

Additional measures of maternal and partner psychological vulnerability, partner infant interaction, infant neurodevelopmental vulnerability, medical outcomes, and measures related to the acceptability and expectancy of the intervention are described in Table 2.

Data collection and visits

Figure 2 summarises study procedures and Table 1 indicates the measures collected at each time point.

Measures

Measures of the primary and secondary outcomes can be found below, measures relating to the 'other outcomes' in the study can be found in Table 2. The time points of when these measures are taken can be seen in Table 1.

Psychological Vulnerability

Clinician-administered PTSD Scale CAPS-5;[101]. The CAPS-5 assesses presence and severity of PTSD symptoms and diagnosis. This gold standard instrument contains 20 items referring to the four symptom clusters, as well as 10 items referring to symptoms duration, distress or impairment, global ratings, and dissociative subtype. Each diagnostic criterion is rated from *0=absent* to *4=extreme/incapacity* in function of symptoms intensity and frequency with a diagnostic cut-off equal to 2. The CAPS-5 has demonstrated good psychometric proprieties[101]. In the absence of a French version at the time when the study was designed, a forward-back method was executed to realise a translation and cultural adaptation[102].

For peer review only - http://bmjopen.bmj.com/site/about/guidelines.xhtml

The PTSD Checklist PCL-5; [103]. This 20-item self-report questionnaire measures symptoms of PTSD over the past month and is used to assess frequency of PTSD symptoms. The PCL-5 refers to the four symptoms clusters of PTSD and scales on a 5-point scale with *0=not at all* and *4=extremely*. Scores are summed to create a total symptom severity score [104]. The French version of the PCL-5 demonstrated strong reliability and validity[105].

Trauma-related intrusive memories diary. Intrusive memories of birth-related trauma experienced during the seven days following ECS are reported in a daily diary, adapted from previous work[4], to assess the frequency of intrusive traumatic memories, . For each intrusion, the time, content and type (intrusive memory, nightmare or other) are recorded, as well as the level of distress on a 5-point scale with ratings of *0=not at all* to *5=extremely.*

Acute Stress Disorder Scale ASDS; [106] This self-assessment instrument measures frequency of ASD symptoms over the last week and is based on DSM 5[107]. Each of the 19 items is scored using a scale from *1=not at all* to *5=extremely*, with a higher score indicating higher ASD symptoms. Good sensitivity and specificity has been reported[106]. The forward-back method was executed to realise a French version translation and cultural adaptation[102].

Anxiety subscale of Hospital Anxiety and Depression Scale HADS; [108]. This self-report questionnaire measures severity of anxiety symptoms during the last week. The anxiety subscale consists of 7 items scored on a 4-point scale (*0=never*, *3=most of the time*). Higher scores indicate higher distress. Good psychometric properties have been reported for the French version[109].

Edinburgh Postnatal Depression Scale (EPDS). This self-assessment examines postnatal depression symptoms over the previous week[110]. The 10 items are scored on a 4-point scale and scores range from 0 to 30. Higher scores indicate higher distress. The French version has demonstrated good psychometric proprieties[111]. A clinical cut-off score of 10.5 has been reported for the use of the French validated version[112].

For peer review only - http://bmjopen.bmj.com/site/about/guidelines.xhtml

Physical and sleep activity. Frequency and duration of maternal sleep and physical activity of the 5 days following childbirth is measured using an accelerometer watch GENEActiv; [113].

Sleep diary. Mothers record their hours of sleep during the week following ECS in a daily sleep diary.

Pittsburgh Sleep Quality Index PSQI; [114]. This 19-item questionnaire measuring sleep during the past month is composed of seven sleep quality-related subscales. The overall score assessing sleep quality is scored by summing the subscales; scores range from 0 to 21. Higher results indicate poor sleep quality while a score of >5 distinguishes good and poor sleepers. The validated French version of the PSQI has shown good psychometrics proprieties[115].

We also included the 10 items of the PSQI-A to assess PTSD-specific sleep disturbances over the past month, answered on a four-point Likert scale (0=*not during the past month*; 3=*three or more times a week*), that assess the frequency of different kinds of trauma-related sleep disturbance. The items are summed to create the total score, where the higher the score the more disturbed the sleep. The validated French version of the PSQI-A has shown good psychometrics[116].

Maternal and infant physiological stress responses.

Physiological regulation. Resting heart rate of the mother and infant are assessed using resting HRV measured by Firstbeat Bodyguard 2 devices providing a continuous measure of cardiac activity. Resting heart rate will be assessed during the 15 minutes resting period before the stress paradigms (see *Physiological stress reactivity* for stress paradigms) at ≤1week and 6 months. Baseline salivary cortisol and cortisol daily profile will be established for the mother and her infant in the two days after leaving the maternity ward (usually the 6th and 7th day postpartum) and for two days at 6 months through salivary sample; 5 saliva

For peer review only - http://bmjopen.bmj.com/site/about/guidelines.xhtml

samples are taken per day, including CAR. Maternal salivary cortisol will be collected using Salivettes© Sarstedt (item number: 51.1534.500) and SalivaBio Infant's Swab (Salimetrics, item number: 5001.08 50) for infants.

Physiological stress reactivity. Maternal and infant stress reactivity will be assessed via salivary cortisol and HRV using Firstbeat Bodyguard 2 devices during the stress phases of their respective stress paradigms at 6 months for the mother and at ≤1week and at 6 months for the infant. The stress paradigm for the mothers is the Trier Social Stress Test (TSST)[117] at 6 months. Maternal salivary cortisol will be measured 7 times before, during, and after the stress paradigms and heart rate throughout the stress paradigm. Stress paradigms for the infants involve the Neonatal Behavioural Assessment Scale (NBAS)[118] at ≤1 week and the double-exposure Face-to-Face Still-Face paradigm (FFSF)[119-122] at 6 months. Salivary cortisol will be measured 3 times, once before and twice after the stressor, and HRV will be measured throughout the stress paradigms. Maternal cortisol will also be collected during the FFSF. Maternal salivary cortisol will be collected using Salivettes© Sarstedt and SalivaBio Infant's Swab (Salimetrics) for infants.

Mother-infant interaction

Mother-to-Infant Bonding Scale (MIBS); [123]. This 8-item questionnaire assesses the mothers' feelings towards her new baby in the first few weeks after birth. Eight adjectives are rated on a scale from *0=very much* to *5=not at all*. Scores are summed to create a total score, with a higher score indicating worse mother-to infant bonding. The MIBS has shown good initial psychometrics[123], and has been validated in French[124].

Emotional Availability Scale (EAS); [125]. Maternal sensitivity and responsiveness will be investigated during a free-play session of mothers with their six-months old. Interactions

For peer review only - http://bmjopen.bmj.com/site/about/guidelines.xhtml

are coded on six dimensions: sensitivity, structuration, intrusion, hostility toward the infant, reactivity to the mother, and maternal involvement[125]. Higher scores on each scale indicate better performance. Two trained psychologists blind to the condition will rate each interaction and inter-observer reliability will be calculated. The EAS shows good psychometric properties[125].

Infant developmental outcomes.

Infant Behaviour Questionnaire – Revised (IBQ-R) Very Short Form; [126] .This is a parent-report questionnaire consisting of 36 items answered on a 7-point Likert-scale (*1=never* to *7=always*). The items assess the frequency of infants' behaviours during the previous two weeks to measure child temperament. The very short form has shown good psychometric properties[126]. There was no available validation French translation. Therefore, the forward-back method was completed to realise a French version translation and cultural adaptation[102]

Bayley Scales of Infant Development (Bayley-lll); [127]. The cognitive, language and motor subscales of the Bayley Scales of Infant Development assess the developmental functioning of the infant. The scales are administered by a trained psychologist or paediatrician through a standard set of play tasks following a standardized protocol. The composite scores for the subscales are age-standardized with a mean score of 100[128].

Sociodemographic, obstetric and neonatal characteristics

Mothers will also report demographic information, including marital status, nationality, profession, level of education[129], and previous and current psychiatric disease as well as any trauma history. Mothers report their height, weight (before pregnancy and current), menstrual cycle, smoking behaviours, and alcohol/drug use. Obstetric data will be

For peer review only - http://bmjopen.bmj.com/site/about/guidelines.xhtml

extracted from the hospital medical record, such as pregnancy-, labour- and birth-related information, gravidity, parity, history of miscarriage, stillbirth, and prematurity, pain, medication, birth control, sexual activity, and psychological support. Neonatal characteristics will be collected from the medical record on severity of morbidity (gestational age and weight at birth, Apgar score, neonatal complications), as well as the Clinical Risk Index for Babies (CRIB II)[130], which represents neonatal morbidity severity.

Sample size calculation

Based on a previous proof-of-principle RCT[4], a sample size of n=120 participants is necessary to have 80% power (α=0.05) to detect a between-group difference of d=0.30 of the primary outcome (PTSD symptoms at 6 weeks). Furthermore, we calculated the sample sizes necessary to obtain significant group differences based on small effect sizes for maternal secondary outcomes (PTSD diagnosis, ASD symptoms and physiological stress reactivity) and infant secondary outcomes (physiological stress reactivity), which range between 56 and 84. Predicting a 20% drop-out rate, we aim to recruit 144 women.

PATIENT AND PUBLIC INVOLVEMENT

The acceptability of the intervention was assessed by participants during the previous proof-of-principle RCT[4]. A questionnaire of satisfaction and acceptability examined the burden of the intervention. An individual feedback and debriefing session will be offered to each participant. Results will be disseminated in written form to the participants and distributed to the public via social media and public events. They will also be discussed with clinical professionals involved in this project.

STATISTICAL ANALYSES

For peer review only - http://bmjopen.bmj.com/site/about/guidelines.xhtml

For the primary analyses, group differences regarding the mean subscale and total scores of the PCL-5 and CAPS-5 at 6 weeks will be analysed using linear regression analysis. Analyses will be adjusted for recruitment centre and the respective baseline values. For secondary aims, the analyses will be performed for differences in changes between groups and differences between groups at different time points in maternal and infant outcomes. Proportion of participants meeting the diagnostic criteria for PTSD at 6 weeks and 6 months between the two groups will also be compared using logistic regression analyses. Furthermore, post-hoc exploratory analyses will be conducted but described as such in publications. All regression analyses regarding cortisol data will be adjusted for potential covariates, such as co-sleeping, breastfeeding, menstrual cycle, and infant and maternal medication. For HRV analyses interbeat intervals (IBIs) from baseline, stress task and recovery will be analysed by the extraction from ECG recordings. Statistical parameters of HRV[132] will be calculated using Kubios HRV Analysis2.2 software[133]. Time domain measures and spectral frequency measures will be used for calculations.

ETHICS AND DISSEMINATION

This study protocol was approved by the Human Research Ethics Committee of the Canton de Vaud (Switzerland, study number 2017-02142) and is registered in *clinicaltrials.gov* (NCT03576586). Participating mothers and, where included, partners will provide their written informed consent. Participation will not interfere with typical care provided to patients after an ECS. Dissemination of results is planned to occur via national and international conferences, in peer-reviewed journals, public conferences, social media, and patient/professional associations.

ACKNOWLEDGMENTS

For peer review only - http://bmjopen.bmj.com/site/about/guidelines.xhtml

We thank Pierluigi Ballabeni for his statistical advice and the generation of the randomization sequence. We also thank our interns for help with study co-ordination and data collection: Lara Schütz, Anna Favero, Yasmina Lotfi, Mathilde Duroux, Cassie Pernet, Daniela Barros Rodrigues, Mélanie Hausser, and Annabelle Reuse. For institutional support, access to the study population, help with recruitment, data collection, and administrative support, we acknowledge Laetitia Accrombessi, Danièle Losguardi, Pascale Gerdy, Maria-Teresa Adjaho, Lauretta Monney, Nadia Carota, Annette Schauenberg, Laetitia Favre, Antonina Chilin, Céline Favrod, and all clinical teams of the labor and postpartum wards at the Lausanne and Geneva University Hospitals.

COLLABORATORS

The following are members of the *START Research Consortium* (including the authors of the present article), listed in alphabetical order:

- Valérie Avignon (Woman-Mother-Child Department, Lausanne University Hospital and University of Lausanne, Lausanne, Switzerland; valerie.avignon@chuv.ch),
- Susan Ayers (Centre for Maternal and Child Health Research, School of Health Sciences, City, University of London, London, UK; susan.ayers.1@city.ac.uk),
- Myriam Bickle-Graz (Neonatology Service, Woman-Mother-Child Department, Lausanne University Hospital and University of Lausanne, Lausanne, Switzerland; myriam.bickle-graz@chuv.ch),
- Julie Bourdin (Woman-Mother-Child Department, Lausanne University Hospital and University of Lausanne, Lausanne, Switzerland; julie.bourdin@chuv.ch),
- Camille Deforges (Institute of Higher Education and Research in Healthcare (IUFRS), University of Lausanne, Lausanne, Switzerland; camille.deforges@chuv.ch),

- Dominique Delecraz (Department of Obstetrics and Gynecology, Geneva University Hospital, Geneva, Switzerland; dominique.delecraz@hcuge.ch),
- David Desseauve (Woman-Mother-Child Department, Lausanne University Hospital and University of Lausanne, Lausanne, Switzerland; david.desseauve@chuv.ch),
- Ulrike Ehlert (Department of Clinical Psychology and Psychotherapy, University of Zurich, Zurich, Switzerland; u.ehlert@psychologie.uzh.ch),
- Manuella Epiney (Department of Obstetrics and Gynecology, Geneva University Hospital, Geneva, Switzerland; manuella.epiney@hcuge.ch),
- Isabelle Eragne (Department of Obstetrics and Gynecology, Geneva University Hospital, Geneva, Switzerland; Isabelle.Eragne@hcuge.ch),
- Emily A. Holmes (Department of Psychology, Uppsala University, Uppsala, Sweden; emily.holmes@psyk.uu.se),
- Antje Horsch (Institute of Higher Education and Research in Healthcare (IUFRS), University of Lausanne and Neonatology Service, Woman-Mother-Child Department, Lausanne University Hospital and University of Lausanne, Lausanne, Switzerland; antje.horsch@chuv.ch),
- Justine Imbert (Woman-Mother-Child Department, Lausanne University Hospital and University of Lausanne, Lausanne, Switzerland; justine.imbert@chuv.ch),
- Nadine Messerli-Bürgy (Clinical Child Psychology & Biological Psychology, University of Fribourg, Fribourg, Switzerland; nadine.messerli@unifr.ch),
- Mathilde Morisod Harari (Service of Child and Adolescent Psychiatry, Lausanne University Hospital and University of Lausanne, Lausanne, Switzerland; mathilde.morisod@chuv.ch),
- Kate Porcheret (Turner Institute for Brain and Mental Health, Monash University, Monash University, Melbourne, Australia; kate.porcheret@monash.edu),

For peer review only

- Véronique Othenin-Girard (Department of Obstetrics and Gynecology, Geneva University Hospital, Geneva, Switzerland; veronique.othenin-girard@hcuge.ch),
- Suzannah Stuijfzand (Institute of Higher Education and Research in Healthcare (IUFRS), University of Lausanne, Lausanne, Switzerland; suzannah.ravenscroft@chuv.ch),
- Alain-Miguel Ribeiro-Almeida (Department of Obstetrics and Gynecology, Geneva University Hospital, Geneva, Switzerland; Alain-Miguel.RibeiroAlmeida@hcuge.ch),
- Vania Sandoz, M.Sc. (Institute of Higher Education and Research in Healthcare (IUFRS), University of Lausanne, Lausanne, Switzerland; vania.sandoz@chuv.ch),
- Daniel S. Schechter (Department of Child & Adolescent Psychiatry, New York University Langone Medical School, New York, NY USA, and Department of Psychiatry, University of Geneva Faculty of Medicine, Geneva, daniel.schechter@hcuge.ch),
- Nicole Sekarski (Paediatric Cardiology Unit, Woman-Mother-Child Department, Lausanne University Hospital and University of Lausanne, Lausanne, Switzerland; nicole.sekarski@chuv.ch),
- Jean-François Tolsa (Department of Woman-Mother-Child, Clinic of Neonatology, Lausanne University Hospital and University of Lausanne, Lausanne, Switzerland; jean-francois.tolsa@chuv.ch),
- Yvan Vial (Obstetrics and Gynecology Service, Woman-Mother-Child Department, Lausanne University Hospital and University of Lausanne, Lausanne, Switzerland; yvan.vial@chuv.ch).

AUTHOR CONTRIBUTIONS

AH designed the study with input from all co-authors and members of the consortium. VS, ME, YV, NS, NMB, UE, MBG, MMH, KP, DS, SA and EH participated in the design of

For peer review only - http://bmjopen.bmj.com/site/about/guidelines.xhtml

the study. AH, VS, CD and SR drafted the manuscript. VS, CD, ME, NMB, MBG, SR, EH and AH significantly contributed to the establishment and refinement of study procedures. All authors critically revised the manuscript and approved the final version of the manuscript.

FUNDING

The START study is funded by a project grant from the Swiss National Science Foundation (SNF 32003B_172982/1).

COMPETING INTERESTS

None declared.

ETHICAL APPROVAL

Ethical approval was granted by the Human Research Ethics Committee of the Canton de Vaud (study number 2017-02142).

For peer review only

REFERENCES

1. Soet JE, Brack GA, DiIorio C. Prevalence and Predictors of Women's Experience of Psychological Trauma During Childbirth. *Birth* 2003;30(1):36-46. doi: 10.1046/j.1523-536X.2003.00215.x

2. American Psychiatric Association. Diagnostic and statistical manual of mental disorders (5th ed.). Arlington, VA: American Psychiatric Publishing 2013.

3. Yildiz PD, Ayers S, Phillips L. The prevalence of posttraumatic stress disorder in pregnancy and after birth: A systematic review and meta-analysis. *Journal of Affective Disorders* 2017;208:634-45. doi: http://dx.doi.org/10.1016/j.jad.2016.10.009

4. Horsch A, Vial Y, Favrod C, et al. Reducing intrusive traumatic memories after emergency caesarean section: A proof-of-principle randomized controlled study. *Behaviour Research and Therapy* 2017;94:36-47. doi: http://doi.org/10.1016/j.brat.2017.03.018

For peer review only - http://bmjopen.bmj.com/site/about/guidelines.xhtml

5. Rogal SS, Poschman K, Belanger K, et al. Effects of posttraumatic stress disorder on pregnancy outcomes. *Journal of affective disorders* 2007;102(1-3):137-43. doi: 10.1016/j.jad.2007.01.003 [published Online First: 2007/02/13]

6. Seng JS, Oakley DJ, Sampselle CM, et al. Posttraumatic stress disorder and pregnancy complications. *Obstet Gynecol* 2001;97 doi: 10.1016/s0029-7844(00)01097-8

7. Cook N, Ayers S, Horsch A. Maternal posttraumatic stress disorder during the perinatal period and child outcomes: A systematic review. *Journal of affective disorders* 2018;225:18-31.

8. Hofberg K, Brockington I. Tokophobia: an unreasoning dread of childbirth. A series of 26 cases. *Br J Psychiatry* 2000;176:83-5. [published Online First: 2000/05/02]

9. Morland L, Goebert D, Onoye J, et al. Posttraumatic stress disorder and pregnancy health: preliminary update and implications. *Psychosomatics* 2007;48(4):304-8. doi: 10.1176/appi.psy.48.4.304 [published Online First: 2007/06/30]

10. Garthus-Niegel S, Horsch A, Ayers S, et al. The influence of postpartum PTSD on breastfeeding: A longitudinal population-based study. *Birth* 2018;45(2):193-201. doi: 10.1111/birt.12328 [published Online First: 2017/12/22]

11. Garthus-Niegel S, Horsch A, Bickle Graz M, et al. The prospective relationship between postpartum PTSD and child sleep: A 2-year follow-up study. *Journal of affective disorders* 2018;241:71-79. doi: 10.1016/j.jad.2018.07.067 [published Online First: 2018/08/12]

12. Garthus-Niegel S, Horsch A, Handtke E, et al. The Impact of Postpartum Posttraumatic Stress and Depression Symptoms on Couples' Relationship Satisfaction: A Population-Based Prospective Study. *Front Psychol* 2018;9:1728. doi: 10.3389/fpsyg.2018.01728 [published Online First: 2018/10/05]

13. Grace SL, Evindar A, Stewart D. The effect of postpartum depression on child cognitive development and behavior: a review and critical analysis of the literature. *Archives of Women's Mental Health* 2003;6(4):263-74.

14. Pearson RM, Bornstein MH, Cordero M, et al. Maternal perinatal mental health and offspring academic achievement at age 16: the mediating role of childhood executive function. *Journal of Child Psychology and Psychiatry* 2016;57(4):491-501.

15. Bauer A, Parsonage M, Knapp M, et al. The costs of perinatal mental health problems. London: Centre for Mental Health and London School of Economics, 2014.

16. Berger A, Bachmann N, Signorell A, et al. Perinatal mental disorders in Switzerland: prevalence estimates and use of mental-health services. *Swiss medical weekly* 2017

17. Horsch A. Post traumatic stress disorder following childbirth and pregnancy loss. *Clinical Psychology in Practice* 2009:274-87.

For peer review only - http://bmjopen.bmj.com/site/about/guidelines.xhtml

18. Bradley R, Slade P. A review of mental health problems in fathers following the birth of a child. *Journal of reproductive and infant psychology* 2011;29(1):19-42.

19. Bradley R, Slade P, Leviston A. Low rates of PTSD in men attending childbirth: A preliminary study. *British Journal of Clinical Psychology* 2008;47(3):295-302. doi: 10.1348/014466508x279495

20. Lane A, Keville R, Morris M, et al. Postnatal depression and elation among mothers and their partners: prevalence and predictors. *The British Journal of Psychiatry* 1997;171(6):550-55.

21. Horsch A, Jacobs I, Gilbert L, et al. Impact of perinatal asphyxia on parental mental health and bonding with the infant: a questionnaire survey of Swiss parents. *BMJ Paediatrics Open* 2017;1(1) doi: 10.1136/bmjpo-2017-000059

22. Ayers S, Wright DB, Wells N. Symptoms of post-traumatic stress disorder in couples after birth: association with the couple's relationship and parent–baby bond. *Journal of Reproductive and Infant Psychology* 2007;25(1):40-50.

23. Lamb ME. The role of the father in child development: John Wiley & Sons 2004.

24. Paulson JF, Keefe HA, Leiferman JA. Early parental depression and child language development. *Journal of Child Psychology and Psychiatry* 2009;50(3):254-62.

25. Ramchandani P, Psychogiou L. Paternal psychiatric disorders and children's psychosocial development. *The Lancet* 2009;374(9690):646-53.

26. De Kloet ER, Joëls M, Holsboer F. Stress and the brain: from adaptation to disease. *Nature Review Neurosciences* 2005;6:463-75.

27. Rohleder N, Nater UM, Wolf JM, et al. Psychosocial stress-induced activation if salivary alpha-amylase. An indicator of sympathetic activity? *Annals of the New York Academy of Sciences* 2004;1032:258-63.

28. Nater UM, Rohleder N, Scholtz W, et al. Determinants of the diurnal course of salivary alpha-amylase. *Psychoneuroendocrinology* 2007;32:392-401.

29. Ehlert U, Gaab J, Heinrichs M. Psychoneuroendocrinological contributions to the etiology of depression, posttraumatic stress disorder, and stress-related bodily disorders: the role of the hypothalamus–pituitary–adrenal axis. *Biological psychology* 2001;57(1-3):141-52.

30. De Kloet ER, Vermetten E, Heijnen CJ, et al. Enhanced cortisol suppression in response to dexamethasone administration in traumatized veterans with and without posttraumatic stress disorder. *Psychoneuroendocrinology* 2007;32:215-26.

31. Neylan TC, Brunet A, Pole N, et al. PTSD symptoms predict waking salivary cortisol levels in police officers. *Psychoneuroendocrinology* 2005;30(4):373-81.

For peer review only

32. Wessa M, Rohleder N, Kirschbaum C, et al. Altered cortisol awakening response in posttraumatic stress disorder. *Psychoneuroendocrinology* 2006;31:209-15.

33. Yehuda R, Golier JA, Kaufman S. Circadian rhythm of salivary cortisol in Holocaust survivors with and without PTSD. *American Journal of Psychiatry* 2005;162:998-1000.

34. Pinto RJ, Correia-Santos P, Costa-Leite J, et al. Cortisol awakening response among women exposed to intimate partner violence. *Psychoneuroendocrinology* 2016;74:57-64. doi: http://dx.doi.org/10.1016/j.psyneuen.2016.08.024

35. Young EA, Breslau N. Cortisol and catecholamines in posttraumatic stress disorder: an epidemiologic community study. *Archives of General Psychiatry* 2004;61:394-401.

36. Shalev AY, Videlock EJ, Peleg T, et al. Stress hormones and posttraumatic stress disorder in civilian trauma victims: a longitudinal study. Part I: HPA axis responses. *Neuropsychopharmacology* 2008;11:365-72.

37. Meewisse ML, Reitsma JB, De Vries G-J, et al. Cortisol and post-traumatic stress disorder in adults: systematic review and meta-analysis. *British Journal of Psychiatry* 2007;191:387-92.

38. Habersaat S, Borghini A, Nessi J, et al. Posttraumatic stress symptoms and cortisol regulation in mothers of very preterm infants. *Stress and Health* 2014;30(2):134-41.

39. Santa Ana EJ, Saladin ME, Back SE, et al. PTSD and the HPA axis: Differences in response to the cold pressor task among individual with child vs. adult trauma. *Psychoneuroendocrinology* 2006;31:501-09.

40. Bremner JD, Vythilingam M, Vermetten E, et al. Cortisol response to a cognitive stress challenge in posttraumatic stress disorder (PTSD) related to childhood abuse. *Psychoneuroendocrinology* 2003;28:733-50.

41. Vyas A, Bernal S, Chattarji S. Effects of chronic stress on dendritic arborization in the central and extended amygdala. *Brain Res* 2003;965(1-2):290-4. [published Online First: 2003/02/20]

42. Fries E, Hesse J, Hellhammer J, et al. A new view on hypocortisolism. *Psychoneuroendocrinology* 2005;30:1010-16.

43. Chrousos GP. Stress and disorders of the stress system. *Nature reviews endocrinology* 2009;5:374-81.

44. Beauchaine TP, Thayer JF. Heart rate variability as a transdiagnostic biomarker of psychopathology. *International journal of psychophysiology : official journal of the International Organization of Psychophysiology* 2015 doi: 10.1016/j.ijpsycho.2015.08.004 [published Online First: 2015/08/15]

45. Nagpal M, Gleichauf K, Ginsberg J. Meta-analysis of heart rate variability as a psychophysiological indicator of posttraumatic stress disorder. *J Trauma Treat* 2013;3(182):2167-1222.1000182.

For peer review only

46. Norte CE, Souza GG, Vilete L, et al. They know their trauma by heart: an assessment of psychophysiological failure to recover in PTSD. *Journal of affective disorders* 2013;150(1):136-41. doi: 10.1016/j.jad.2012.11.039 [published Online First: 2013/01/01]

47. Association AP. Diagnostic and Statistial Manual of Mental Disorders. Fifth ed: American Psychiatric Association 2013.

48. Maher MJ, Rego SA, Asnis GM. Sleep disturbances in patients with post-traumatic stress disorder: epidemiology, impact and approaches to management. *CNS drugs* 2006;20(7):567-90.

49. Porcheret K, Holmes EA, Goodwin GM, et al. Psychological impact of an analogue traumatic event reduced by sleep deprivation. *Sleep* 2015;38(7):1017-25. doi: http://dx.doi.org/10.5665/sleep.4802

50. Kleim B, Wysokowsky J, Schmid N, et al. Effects of sleep after experimental trauma on intrusive emotional memories. *Sleep* 2016;39(12):2125-32.

51. Woud ML, Cwik JC, Blackwell SE, et al. Does napping enhance the effects of Cognitive Bias Modification-Appraisal training? An experimental study. *PloS one* 2018;13(2):e0192837.

52. Porcheret K, van Heugten–van der Kloet D, Goodwin GM, et al. Investigation of the impact of total sleep deprivation at home on the number of intrusive memories to an analogue trauma. *Translational psychiatry* 2019;9(1):104.

53. Feldman R. Parent-Infant synchrony and the construction of shared timing: psychological precursors, developmental outcomes, and risk conditions *Journal of Child Psychology and Psychiatry* 2007;48(3/4):329-54.

54. McCormick MC, Workman-Daniels K, Brooks-Gunn J. The behavioral and emotional well-being of school-age children with differen birth weights. *Pediatrics* 1996;97(1):18-25.

55. Treyvaud K, Anderson VA, Lee KJ, et al. Parental mental health and early social-emotional development of children born very preterm. *Journal of pediatric psychology* 2010;35(7):768-77. doi: 10.1093/jpepsy/jsp109 [published Online First: 2009/12/04]

56. Blandon AY, Calkins SD, Keane SP, et al. Individual differences in trajectories of emotion regulation processes: the effects of maternal depressive symptomatology and children's physiological regulation. *Developmental Psychology* 2008;44(4):1110-23.

57. Eisenberg N, Valiente C, Morris AS, et al. Longitudinal relations among parental emotional expressivity, children's regulation, and quality of socio-emotional functioning *Developmental Psychology* 2003;39(1):3-19.

58. Feng X, Shaw DS, Kovacs M, et al. Emotion regulation in preschoolers: the roles of behavioral inhibition, maternal affective behavior, and maternal depression. *Journal of Child Psychology and Psychiatry* 2008;49(2):132-41.

For peer review only - http://bmjopen.bmj.com/site/about/guidelines.xhtml

59. Francis DD, Meaney MJ. Maternal care and the development of stress responses. *Current Opinion in Neurobiology* 1999;9:128-34.

60. Liu D, Diorio J, Tannenbaum B, et al. Maternal care, hippocampal glucocorticoid receptors, and hypothalamic-pituitary-adrenal responses to stress. *Science* 1997;277:1659-62.

61. Gunzenhauser N. Infant stimulation: for whom, what kind, when, and how much? Safe Harbor, CT: Johnson and Johnson 1987.

62. Schore AN. Affect regulation and the origin of the self. Hillsdale, NJ: Lawrence Erlbaum Associates 1994.

63. Liu K, Ruggero CJ, Goldstein B, et al. Elevated cortisol in healthy female adolescent offspring of mothers with posttraumatic stress disorder. *Journal of Anxiety Disorders* 2016;40:37-43. doi: http://dx.doi.org/10.1016/j.janxdis.2016.04.003

64. Essex MJ, Klein MH, Cho E, et al. Maternal stress begining in infancy may sensitize children to later stress exposure : Effects on cortisol and behavior. *Biological Psychiatry* 2002;52:776 - 84

65. Lupien SJ, King S, Meaney MJ, et al. Child's stress hormone levels correlate with mother's socio-economic status and depressive state. *Biological Psychiatry* 2000;48:976 - 80.

66. Stenius F, Theorell TL, Scheynius A, et al. Salivary cortisol levels in 6-months-olds and their parents. *Allergy* 2008;63(88):supplement 485.

67. Warren SL, Gunnar MR, Kagan J, et al. Maternal panic disorder: infant temperament, neurophysiology and parentiong behaviors. *Journal of the American Academy of Child and Adolescent Psychiatry* 2003;42(7):814 - 25

68. Yehuda R, Halligan SL, Bierer LM. Cortisol levels in adult offspring of Holocaust survivors: relation to PTSD symptom severity in the parent and child. *Psychoneuroendocrinology* 2002;27(1-2):171-80. [published Online First: 2001/12/26]

69. Ehlert U. Understanding the trans-generational consequences of prenatal stress. *Journal of psychosomatic research* 2013;75(4):297.

70. Muller-Nix C, Forcada-Guex M, Pierrehumbert B, et al. Prematurity, maternal stress and mother-child interactions. *Early Human Development* 2004;79:145-58.

71. Schmücker G, Brisch K-H, Köhntop B, et al. The influence of prematurity, maternal anxiety, and infants' neurobiological risk on mother - infant interactions. *Infant Mental Health Journal* 2005;26(5):423 - 41.

72. Zelkowitz P, Papageorgiou A. Maternal anxiety: an emerging prognostic factor in neonatology. *Acta Paediatrica Academiae* 2005;94(12):1704 - 05

For peer review only - http://bmjopen.bmj.com/site/about/guidelines.xhtml

73. Korja R, Savonlahti E, Ahlqvist-Bjorkroth S, et al. Maternal depression is associated with mother-infant interaction in preterm infants. *Acta Paediatr* 2008;97(6):724-30. doi: 10.1111/j.1651-2227.2008.00733.x [published Online First: 2008/04/01]

74. Crockett EE, Holmes BM, Granger DA, et al. Maternal Disrupted Communication During Face-to-Face Interaction at 4 months: Relation to Maternal and Infant Cortisol Among at-Risk Families. *Infancy : the official journal of the International Society on Infant Studies* 2013;18(6):1111-34. doi: 10.1111/infa.12015

75. Ahnert L, Gunnar MR, Lamb ME, et al. Transition to Child Care: Associations with Infant–Mother Attachment, Infant Negative Emotion, and Cortisol Elevations. *Child Development* 2004;75(3):639-50.

76. Domes G, Heinrichs M, Reichwald U, et al. Hypothalamic-pituitary-adrenal axis reactivity to psychological stress and memory in middle-aged women: high responders exhibit enhanced declarative memory performance. *Psychoneuroendocrinology* 2002;27(7):843 - 53

77. Nachmias M, Gunnar M, Mangelsdorf S, et al. Behavioral inhibition and stress reactivity: the moderating role of attachment security. *Child Dev* 1996;67(2):508-22.

78. Spangler G, Scheubeck R. Behavioral organization in newborns and its relation to adrenocortical and cardiac activity. *Child Development* 1993;64:622-33.

79. Cordero MI, Moser DA, Manini A, et al. Effects of interpersonal violence-related post-traumatic stress disorder (PTSD) on mother and child diurnal cortisol rhythm and cortisol reactivity to a laboratory stressor involving separation. *Hormones and Behavior* 2017;90:15-24. doi: https://doi.org/10.1016/j.yhbeh.2017.02.007

80. Dierckx B, Tulen JH, van den Berg MP, et al. Maternal psychopathology influences infant heart rate variability: Generation R Study. *Psychosom Med* 2009;71(3):313-21. doi: 10.1097/PSY.0b013e318198a82c [published Online First: 2009/03/03]

81. Braeken MA, Kemp AH, Outhred T, et al. Pregnant mothers with resolved anxiety disorders and their offspring have reduced heart rate variability: implications for the health of children. *PloS one* 2013;8(12):e83186. doi: 10.1371/journal.pone.0083186 [published Online First: 2013/12/18]

82. Jovanovic T, Smith A, Kamkwalala A, et al. Physiological markers of anxiety are increased in children of abused mothers. *Journal of child psychology and psychiatry, and allied disciplines* 2011;52(8):844-52. doi: 10.1111/j.1469-7610.2011.02410.x [published Online First: 2011/04/20]

83. Bastos MH, Furuta M, Small R, et al. Debriefing interventions for the prevention of psychological trauma in women following childbirth. *The Cochrane Library* 2015

84. O'Donnell ML, Elliott P, Lau W, et al. PTSD symptom trajectories: From early to chronic response. *Behaviour research and therapy* 2007;45:601-06.

For peer review only - http://bmjopen.bmj.com/site/about/guidelines.xhtml

85. Brewin CR. Episodic memory, perceptual memory, and their interaction: Foundations for a theory of posttraumatic stress disorder. 2013

86. Brewin CR. Episodic memory, perceptual memory, and their interaction: foundations for a theory of posttraumatic stress disorder. *Psychological bulletin* 2014;140(1):69-97. doi: 10.1037/a0033722 [published Online First: 2013/08/07]

87. Holmes EA, James EL, Coode-Bate T, et al. Can playing the computer game "Tetris" reduce the build-up of flashbacks for trauma? A proposal from cognitive science. *PloS one* 2009;4(1):e4153. doi: 10.1371/journal.pone.0004153 [published Online First: 2009/01/08]

88. Holmes EA, James EL, Kilford EJ, et al. Key steps in developing a cognitive vaccine against traumatic flashbacks: visuospatial Tetris versus verbal Pub Quiz. *PloS one* 2010;5(11):e13706. doi: 10.1371/journal.pone.0013706

89. Kindt M, van den Hout M, Arntz A, et al. The influence of data-driven versus conceptually-driven processing on the development of PTSD-like symptoms. *Journal of behavior therapy and experimental psychiatry* 2008;39(4):546-57. doi: 10.1016/j.jbtep.2007.12.003 [published Online First: 2008/03/11]

90. Iyadurai L, Visser RM, Lau-Zhu A, et al. Intrusive memories of trauma: A target for research bridging cognitive science and its clinical application. *Clinical psychology review* 2018

91. Deeprose C, Zhang S, Dejong H, et al. Imagery in the aftermath of viewing a traumatic film: Using cognitive tasks to modulate the development of involuntary memory. *Journal of behavior therapy and experimental psychiatry* 2012;43(2):758-64. doi: 10.1016/j.jbtep.2011.10.008

92. Walker MP, Brakefield T, Hobson JA, et al. Dissociable stages of human memory consolidation and reconsolidation. *Nature* 2003;425(6958):616-20. doi: 10.1038/nature01930 [published Online First: 2003/10/10]

93. Iyadurai L, Blackwell SE, Meiser-Stedman R, et al. Preventing intrusive memories after trauma via a brief intervention involving Tetris computer game play in the emergency department: a proof-of-concept randomized controlled trial. *Molecular psychiatry* 2018;23(3):674.

94. Foa EB, Cashman L, Jaycox L, et al. The validation of a self-report measure of posttraumatic stress disorder: the Posttraumatic Diagnostic Scale. *Psychological assessment* 1997;9(4):445.

95. Kim J, Shin W. How to Do Random Allocation (Randomization). *Clinics in Orthopedic Surgery* 2014;6(1):103-09. doi: 10.4055/cios.2014.6.1.103

96. Furuta M, Horsch A, Ng ES, et al. Effectiveness of trauma-focused psychological therapies for treating post-traumatic stress disorder symptoms in women following childbirth: a systematic review and meta-analysis. *Frontiers in psychiatry* 2018;9:591.

For peer review only - http://bmjopen.bmj.com/site/about/guidelines.xhtml

97. Roberts NP, Kitchiner NJ, Kenardy J, et al. Multiple session early psychological interventions for the prevention of post-traumatic stress disorder. *Cochrane Database of Systematic Reviews* 2009;Art. No.: CD006869(3) doi: 101002/14651858.CD006869.

98. Sijbrandij M, Kleiboer A, Bisson JI, et al. Pharmacological prevention of post-traumatic stress disorder and acute stress disorder: a systematic review and meta-analysis. *The Lancet Psychiatry* 2015;2(5):413-21.

99. Rose SC, Bisson J, Churchill R, et al. Psychological debriefing for preventing post traumatic stress disorder (PTSD). *The Cochrane Library* 2002

100. Bourne C, Frasquilho F, Roth AD, et al. Is it mere distraction? Peri-traumatic verbal tasks can increase analogue flashbacks but reduce voluntary memory performance. *Journal of behavior therapy and experimental psychiatry* 2010;41(3):316-24.

101. Weathers FW, Bovin MJ, Lee DJ, et al. The Clinician-Administered PTSD Scale for DSM–5 (CAPS-5): Development and Initial Psychometric Evaluation in Military Veterans. *Psychological Assessment* 2017:No Pagination Specified-No Pagination Specified. doi: 10.1037/pas0000486

102. Wild D, Grove A, Martin M, et al. Principles of Good Practice for the Translation and Cultural Adaptation Process for Patient-Reported Outcomes (PRO) Measures: report of the ISPOR Task Force for Translation and Cultural Adaptation. *Value Health* 2005;8(2):94-104. doi: 10.1111/j.1524-4733.2005.04054.x [published Online First: 2005/04/05]

103. Blevins CA, Weathers FW, Davis MT, et al. The posttraumatic stress disorder checklist for DSM-5 (PCL-5): Development and initial psychometric evaluation. *Journal of Traumatic Stress* 2015;28(6):489-98.

104. Bovin MJ, Marx BP, Weathers FW, et al. Psychometric properties of the PTSD checklist for diagnostic and statistical manual of mental disorders–fifth edition (PCL-5) in veterans. *Psychological Assessment* 2016;28(11):1379.

105. Ashbaugh AR, Houle-Johnson S, Herbert C, et al. Psychometric Validation of the English and French Versions of the Posttraumatic Stress Disorder Checklist for DSM-5 (PCL-5). *PloS one* 2016;11(10):e0161645. doi: 10.1371/journal.pone.0161645

106. Bryant RA, Moulds ML, Guthrie RM. Acute Stress Disorder Scale: a self-report measure of acute stress disorder. *Psychological Assessment* 2000;12(1):61-68.

107. American Psychiatric Association. Diagnostic and statistical manual of mental disorders. 4th ed., text rev. ed. Washington, DC: American Psychiatric Publishing 2000.

108. Zigmond AS, Snaith RP. The hospital anxiety and depression scale. *Acta psychiatrica scandinavica* 1983;67(6):361-70.

For peer review only

For peer review only - http://bmjopen.bmj.com/site/about/guidelines.xhtml

109. Bocerean C, Dupret E. A validation study of the Hospital Anxiety and Depression Scale (HADS) in a large sample of French employees. *BMC Psychiatry* 2014;14:354. doi: 10.1186/s12888-014-0354-0 [published Online First: 2014/12/17]

110. Cox JL, Holden JM, Sagovsky R. Detection of postnatal depression. Development of the 10-item Edinburgh Postnatal Depression Scale. *The British journal of psychiatry : the journal of mental science* 1987;150:782-6.

111. Guedeney N, Fermanian J. Validation study of the French version of the Edinburgh Postnatal Depression Scale (EPDS): new results about use and psychometric properties. *Eur Psychiatry* 1998;13(2):83-9. doi: 10.1016/S0924-9338(98)80023-0 [published Online First: 1998/01/01]

112. Guedeney N, Fermanian J. Validation study of the French version of the Edinburgh Postnatal Depression Scale (EPDS): new results about use and psychometric properties. *European psychiatry* 1998;13(2):83-89.

113. Esliger DW, Rowlands AV, Hurst TL, et al. Validation of the GENEA Accelerometer. 2011

114. Buysse DJ, Reynolds CF, Monk TH, et al. The Pittsburgh sleep quality index: A new instrument for psychiatric practice and research. *Psychiatry research* 1989;28(2):193-213.

115. Blais F, Gendron L, Mimeault V, et al. Evaluation de l'insomnie: Validation de trois questionnaires. *L'Encéphale: Revue de psychiatrie clinique biologique et thérapeutique* 1997

116. Ait-Aoudia M, Levy PP, Bui E, et al. Validation of the French version of the Pittsburgh Sleep Quality Index Addendum for posttraumatic stress disorder. *European journal of psychotraumatology* 2013;4 doi: 10.3402/ejpt.v4i0.19298 [published Online First: 2013/09/18]

117. Kirschbaum C, Pirke K-M, Hellhammer DH. The 'Trier Social Stress Test'–a tool for investigating psychobiological stress responses in a laboratory setting. *Neuropsychobiology* 1993;28(1-2):76-81.

118. Brazelton TB, Nugent JK. The Neontal Behavioral Assessment Scale. 4th ed. London: Mac Keith Press 2011.

119. Tronick E, Als H, Adamson L, et al. The infant's response to entrapment between contradictory messages in face-to-face interaction. *Journal of the American Academy of Child Psychiatry* 1978;17(1):1-13.

120. Provenzi L, Giusti L, Montirosso R. Do infants exhibit significant cortisol reactivity to the Face-to-Face Still-Face paradigm? A narrative review and meta-analysis. *Developmental Review* 2016;42:34-55.

For peer review only - http://bmjopen.bmj.com/site/about/guidelines.xhtml

121. Haley DW, Stansbury K. Infant stress and parent responsiveness: regulation of physiology and behavior during still-face and reunion. *Child development* 2003;74(5):1534-46.

122. DiCorcia JA, Snidman N, Sravish AV, et al. Evaluating the Nature of the Still-Face Effect in the Double Face-to-Face Still-Face Paradigm Using Different Comparison Groups. *Infancy* 2016;21(3):332-52.

123. Taylor A, Atkins R, Kumar R, et al. A new Mother-to-Infant Bonding Scale: links with early maternal mood. *Archives of women's mental health* 2005;8(1):45-51.

124. Bienfait M, Haquet A, Maury M, et al. Traduction française de l'autoquestionnaire MIBS (Mother to Infant Bonding Scale) et validation comme évaluation du lien mère-nouveau-né en maternité. . *Devenir* 2017;29(4):233-53.

125. Biringen Z, Robinson JL, Emde RN. Appendix B: The emotional availability scales (; an abridged infancy/early childhood version). *Attachment & human development* 2000;2(2):256-70.

126. Putnam SP, Helbig AL, Gartstein MA, et al. Development and Assessment of Short and Very Short Forms of the Infant Behavior Questionnaire–Revised. *Journal of Personality Assessment* 2014;96(4):445-58. doi: 10.1080/00223891.2013.841171

127. Bayley N, Reuner G. Bayley scales of infant and toddler development: Bayley-III: Harcourt Assessment, Psych. Corporation San Antonio, Tex, USA 2006.

128. Albers EM, Marianne Riksen-Walraven J, Sweep FC, et al. Maternal behavior predicts infant cortisol recovery from a mild everyday stressor. *Journal of Child Psychology and Psychiatry* 2008;49(1):97-103.

129. Largo RH, Pfister D, Molinari L, et al. Significance of prenatal, perinatal and postnatal factors in the development of AGA preterm infants at five to seven years. *Dev Med Child Neurol* 1989;31(4):440-56. [published Online First: 1989/08/01]

130. Parry G, Tucker J, Tarnow-Mordi W, et al. CRIB II: an update of the clinical risk index for babies score. *Lancet* 2003;361(9371):1789-91. doi: 10.1016/S0140-6736(03)13397-1 [published Online First: 2003/06/05]

131. Pruessner JC, Kirschbaum C, Meinlschmid G, et al. Two formulas for computation of the area under the curve represent measures of total hormone concentration versus time-dependent change. *Psychoneuroendocrinology* 2003;28(7):916-31.

132. Camm AJ, Malik M, Bigger J, et al. Heart rate variability: standards of measurement, physiological interpretation and clinical use. Task Force of the European Society of Cardiology and the North American Society of Pacing and Electrophysiology. *Circulation* 1996;93(5):1043-65.

For peer review only - http://bmjopen.bmj.com/site/about/guidelines.xhtml

133. Tarvainen MP, Niskanen JP, Lipponen JA, et al. Kubios HRV--heart rate variability analysis software. *Computer methods and programs in biomedicine* 2014;113(1):210-20. doi: 10.1016/j.cmpb.2013.07.024 [published Online First: 2013/09/24]

For peer review only

For peer review only - http://bmjopen.bmj.com/site/about/guidelines.xhtml

Table 1. Overview of primary, secondary and other outcomes, measures, and time points

	DOMAIN	VARIABLES	INSTRUMENTS	TIMING<br>6 h after ECS (T1)	≤ 1 week (T2)	6 weeks (T3)	6 months (T4)
**MOTHER**	Sociodemographic and medical data	Sociodemographic variables	Demographic questionnaire		**X**	**X**	**X**
		Medical data	Obstetric data and pregnancy outcomes from medical records		**X**		**X**
			Menstrual cycle (if applicable)				**X**
			Breastfeeding diary/questions		**X**	**X**	**X**
	Acceptability and expectancy of the intervention	Satisfaction and expectancy	Feedback questionnaire	**X**			
	Maternal psychological vulnerability	Intrusive trauma-related memories	Trauma-related intrusive memories diary [1]		**X**		
		Stress exposure and perception	Post-delivery Perceived Stress Inventory (PDPSI) [2]		**X**		
			Major life events [3, 4]		**X**		**X**
			Postnatal Perceived Stress Inventory (PPSI) [5]				**X**

For peer review only

		Parenting Stress Index – Short Form (PSI-SF) [6]				X
	PTSD	Clinician-administered PTSD scale (CAPS) [7]			X	X
		PTSD Checklist (PCL-5) [8]			X	X
	ASD	Acute Stress Disorder Scale (ASDS) [9]	X	X		
	Anxiety	Hospital Anxiety and Depression Scale: anxiety subscale (HADS) [10]	X	X	X	X
	Depression	Edinburgh Postnatal Depression Scale (EPDS) [11]	X	X	X	X
	Social support	Modified Medical Outcomes Study Social Support Survey (MOS-8) [12]		X	X	X
		Revised Dyadic Adjustment Scale (RDAS) [13, 14]		X	X	X
	Sleep	Pittsburgh Sleep Quality Index Addendum for posttraumatic stress disorder (PSQI-A) [15]			X	X

For peer review only

			Morningness-Eveningness questionnaire (MEQ) [16]		**X**		
			Pittsburgh Sleep Quality Index (PSQI) [17]		**X**	**X**	**X**
			Sleep diary		**X**		
			Overnight accelerometer assessments (GeneActiv®)		**X**		**X**
	Maternal physiological stress responses	Physiological regulation	Baseline cortisol and cortisol daily profile (saliva)		**X**		**X**
			Resting heart rate and heart rate variability (Firstbeat Bodyguard 2)				**X**
		Physiological stress reactivity	Cortisol (saliva)				**X**
			Heart rate variability (Firstbeat Bodyguard 2)				**X**
**PARTNER**	Sociodemographic data	Sociodemographic variables	Demographic questionnaire		**X**	**X**	**X**
	Paternal psychological vulnerability	Intrusive traumatic memories	Traumatic intrusions diary [1]		**X**		
		Stress exposure and perception	Major life events [3, 4]		**X**		**X**

For peer review only

			Parenting Stress Index – Short Form (PSI-SF) [6]				**X**
		PTSD	Clinician-administered PTSD scale (CAPS) [7]			**X**	**X**
			PTSD Checklist (PCL-5) [8]			**X**	**X**
		ASD	Acute Stress Disorder Scale (ASDS) [9]		**X**		
		Anxiety	Hospital Anxiety and Depression Scale: anxiety subscale (HADS) [10]		**X**	**X**	**X**
		Depression	Edinburgh Postnatal Depression Scale (EPDS) [11]		**X**	**X**	**X**
		Social support	Modified Medical Outcomes Study Social Support Survey (MOS-8) [12]		**X**	**X**	**X**
			Revised Dyadic Adjustment Scale (RDAS) [13, 14]		**X**	**X**	**X**
		Perception of ECS-related trauma	Screening questions of the Posttraumatic Diagnostic Scale [18]		**X**		

		Sleep	Pittsburgh Sleep Quality Index (PSQI) [17]		X	X	X
			Pittsburgh Sleep Quality Index Addendum for posttraumatic stress disorder (PSQI-A)[15]			X	X
			Morningness-Eveningness questionnaire (MEQ) [16]		X		
	Partner physiological responses	Physiological regulation	Cortisol daily profile (saliva)		X		X
**INFANT**	Sociodemographic and medical data	Sociodemographic variables	Demographic questionnaire (completed by mother)		X	X	X
		Medical data	Neonatal outcomes from medical records		X		
	Infant neurodevelopmental vulnerability	Infant irritability	Dubowitz neurologic examination [19]		X		
	Infant physiological stress responses	Physiological regulation	Baseline cortisol and cortisol daily profile (saliva)		X		X
			Resting heart rate and heart rate variability by (Firstbeat Bodyguard 2)		X		X
		Physiological	Cortisol (saliva)		X		X

For peer review only - http://bmjopen.bmj.com/site/about/guidelines.xhtml

		stress reactivity					
			Heart rate variability (Firstbeat Bodyguard 2)		**X**		**X**
	Developmental outcomes	Neonatal behaviour	Neonatal Behavioural Assessment Scale (NBAS) [20]		**X**		
		Infant development	Infant Behaviour Questionnaire-revised (IBQ-R) [21]				**X** *
			Bayley Scales of Infant Development (Bayley-III; clinician-rated) [22]				**X**
**PARENT-INFANT INTERACTION**	Mother-infant interaction	Maternal sensitivity	Emotional Availability Scale (EAS, clinician-rated) [23]				**X**
		Bonding	Mother-to-Infant-Bonding Scale (MIBS) [24]		**X**	**X**	**X**
	Partner-infant interaction	Bonding	Father-Infant-Bonding Scale (FIBS) [24]		**X**	**X**	**X**

Note: * = completed by mothers and partners

For peer review only

Table 2. Other Outcomes and a brief description of the measures used for each outcome. Participant groups for whom the outcome is relevant are highlighted in bold.

Domain	Instruments	Description
**Maternal and paternal psychological vulnerability**	Morningness-Eveningness Questionnaire (MEQ) [16]	19 multiple-choice questions self-assessment questionnaire investigating the **mother and partner**'s circadian rhythm. Scores are summed to create a total and a higher score indicates a more 'morning' person.
	Modified Medical Outcomes Study Social Support Survey (MOS-8) [12] [25]	8-item self-report questionnaire assessing **mother and partner** social support across four functional support scales: emotional/ informative, tangible, affectionate, and positive social interaction. Items are answered on a 6 point scale (0= *Never*, 5 = *Always*).
	Revised Dyadic Adjustment Scale (RDAS) [13] [26]	14-item self-report questionnaire measuring the **mother and the partner** relationship satisfaction. Three categories are evaluated: consensus, satisfaction and cohesion.
	Parenting Stress Index - Short Form (PSI-SF)[27] [28]	Self-report questionnaire measuring the **mother and partner** parenting stress. It consists of three subscales: parental distress, parent-child dysfunctional interactions, and child difficulties. 36 items are answered on a 1-point Likert scale *(1 = Strongly agree, 5 = Strongly disagree).*
	The Post-delivery Perceived Stress Inventory (PDPSI) [2]	16-item self-report questionnaire assessing **mother's** perceived stress linked to delivery. Each item is a potential stressor the

For peer review only - http://bmjopen.bmj.com/site/about/guidelines.xhtml

	mother may have experienced during or after delivery. Mothers are asked whether they found the items more or less stressful using a 5-point Likert scale *(1=Never, 5=Very often)*.
Postnatal Perceived Stress Inventory (PPSI)[5]	19-item self-report questionnaire assessing **maternal** postpartum perceived stress. Each item relates to a potential stressor they may encounter within the postnatal period. Mothers are asked to indicate whether they were more or less stressed on a 5 point scale (1 = Not at all, 5 = Extremely).
Life Events Questionnaire[4, 29]	15-item self-report scale where **mother and partners** are asked to indicate if they have experienced different major life events and how stressful they found these events. One item allows participants to identify an event not mentioned and one asks if a major life event occurred within the first trimester of pregnancy.
Trauma-related intrusive memories diary [1]	Completed by **partners**; see Measures section for more details.
Clinician-administered PTSD scale (CAPS) [7]	Completed by **partners**; see Measures section for more details.
PTSD Checklist (PCL-5) [8]	Completed by **partners**; see Measures section for more details.
Acute Stress Disorder Scale (ASDS) [9]	Completed by **partners**; see Measures section for more details.

For peer review only - http://bmjopen.bmj.com/site/about/guidelines.xhtml

	Edinburgh Postnatal Depression Scale (EPDS) [11]	Completed by **partners**; see Measures section for more details.
	Anxiety subscale of Hospital Anxiety and Depression Scale (HADS) [10]	Completed by **partners**; see Measures section for more details.
	Pittsburgh Sleep Quality Index (PSQI) [15, 17]	Completed by **partners; s**ee Measures section for more details.
	Pittsburgh Sleep Quality Index Addendum for posttraumatic stress disorder (PSQI-A) [15]	Completed by **partners**; see Measures section for more details.
	Perception of ECS-related trauma	**Partners** complete the 4 screening questions of the Posttraumatic Diagnostic Scale [30]
**Partner physiological regulation**	Cortisol daily profile	Completed by **partners**; see Measures section for more details.
**Partner-infant interaction**	Mother-to-Infant-Bonding Scale (MIBS) [24]	Adapted for partners, **s**ee Measures section for more details
**Demographic Information**	Partner demographics	Adapted for partners, **s**ee Measures section for more details
	Infant demographics	**Mothers** report on their child's weight and height.
**Medical data**	Breastfeeding diary and questions	**Mothers** report on breastfeeding in a 2-day daily diary including breastfeeding length and type (exclusive or mixed).
**Acceptability and expectancy of the intervention**	Self-report questionnaire of satisfaction and acceptability of the intervention	On completing the intervention activity, **mothers** complete 7 items to assess intervention fidelity, satisfaction and acceptability of the intervention.
**Infant neurodevelopmental vulnerability**	Dubowitz neurologic examination [19]	Examination of the **infant** on 34 items subdivided into 6 categories (tone, tone patterns, reflexes, movements, abnormal signs,

For peer review only - http://bmjopen.bmj.com/site/about/guidelines.xhtml

		and behavior). Full examination by a pediatrician follows standardised instructions and takes 10-15 minutes.

For peer review only

For peer review only - http://bmjopen.bmj.com/site/about/guidelines.xhtml

1. James, E.L., et al., *Computer Game Play Reduces Intrusive Memories of Experimental Trauma via Reconsolidation-Update Mechanisms.* Psychol Sci, 2015. **26**(8): p. 1201-15.
2. Razurel, C., et al., *Validation of the post-delivery perceived stress inventory.* Psychology, health & medicine, 2014. **19**(1): p. 70-82.
3. Koch, F.S., A. Sepa, and J. Ludvigsson, *Psychological stress and obesity.* J Pediatr, 2008. **153**(6): p. 839-44.
4. Obel, C., et al., *Stress and salivary cortisol during pregnancy.* Psychoneuroendocrinology, 2005. **30**(7): p. 647-656.
5. Razurel, C., et al., *Validation of the Postnatal Perceived Stress Inventory in a French Speaking Population of Primiparous Women.* Journal of Obstetric, Gynecologic, & Neonatal Nursing, 2013. **42**(6): p. 685-696.
6. Abidin, R.R., *Parenting Stress Index : Professional Manual*. 3rd ed. 1995, Odessa, FL: Psychological Assessment Resources, Inc.
7. Blake, D.D., et al., *The development of a clinician-administered PTSD scale.* Journal of Traumatic Stress, 1995. **8**: p. 75-90.
8. Weathers, F., et al., *The PTSD checklist (PCL): reliability, validity, and diagnostic utility*, in *Annual Meeting of the International Society for Traumatic Stress Studies*. 1993.
9. Bryant, R.A., M.L. Moulds, and R.M. Guthrie, *Acute Stress Disorder Scale: a self-report measure of acute stress disorder.* Psychological Assessment, 2000. **12**(1): p. 61-68.
10. Zigmond, A.S. and R.P. Snaith, *The hospital anxiety and depression scale.* Acta psychiatrica scandinavica, 1983. **67**(6): p. 361-370.
11. Cox, J.L., J.M. Holden, and R. Sagovsky, *Detection of postnatal depression. Development of the 10-item Edinburgh Postnatal Depression Scale.* Br J Psychiatry, 1987. **150**: p. 782-6.
12. Moser, A., et al., *The eight-item modified Medical Outcomes Study Social Support Survey: psychometric evaluation showed excellent performance.* Journal of Clinical Epidemiology, 2012. **65**(10): p. 1107-1116.
13. Busby, D.M., et al., *A REVISION OF THE DYADIC ADJUSTMENT SCALE FOR USE WITH DISTRESSED AND NONDISTRESSED COUPLES: CONSTRUCT HIERARCHY AND MULTIDIMENSIONAL SCALES.* Journal of Marital and Family Therapy, 1995. **21**(3): p. 289-308.
14. Crane, D.R., K.C. Middleton, and R.A. Bean, *Establishing Criterion Scores for the Kansas Marital Satisfaction Scale and the Revised Dyadic Adjustment Scale.* The American Journal of Family Therapy, 2000. **28**(1): p. 53-60.
15. Ait-Aoudia, M., et al., *Validation of the French version of the Pittsburgh Sleep Quality Index Addendum for posttraumatic stress disorder.* Eur J Psychotraumatol, 2013. **4**.
16. Horne, J.A. and O. Ostberg, *A self-assessment questionnaire to determine morningness-eveningness in human circadian rhythms.* International Journal of Chronobiology, 1976. **4**(2): p. 97-110.
17. Buysse, D.J., et al., *The Pittsburgh sleep quality index: A new instrument for psychiatric practice and research.* Psychiatry Research, 1989. **28**(2): p. 193-213.
18. Foa, E.B., et al., *The validation of a self-report measure of posttraumatic stress disorder: The Posttraumatic Diagnostic Scale.* Psychological Assessment, 1997. **9**(4): p. 445-451.

For peer review only - http://bmjopen.bmj.com/site/about/guidelines.xhtml

19. Dubowitz, L., E. Mercuri, and V. Dubowitz, *An optimality score for the neurologic examination of the term newborn.* The Journal of pediatrics, 1998. **133**(3): p. 406-416.
20. Brazelton, T.B., *Neonatal Behavioral Assessment Scale*. 1st ed. 1973, London: Spastics International Medical Publications.
21. Putnam, S.P., et al., *Development and Assessment of Short and Very Short Forms of the Infant Behavior Questionnaire–Revised.* Journal of Personality Assessment, 2014. **96**(4): p. 445-458.
22. Bayley, N. and G. Reuner, *Bayley scales of infant and toddler development: Bayley-III*. Vol. 7. 2006: Harcourt Assessment, Psych. Corporation San Antonio, Tex, USA.
23. Biringen, Z., J.L. Robinson, and R.N. Emde, *Appendix B: The emotional availability scales (; an abridged infancy/early childhood version).* Attachment & human development, 2000. **2**(2): p. 256-270.
24. Taylor, A., et al., *A new Mother-to-Infant Bonding Scale: links with early maternal mood.* Archives of women's mental health, 2005. **8**(1): p. 45-51.
25. Bienfait, M., et al., *Traduction française de l'autoquestionnaire MIBS (Mother to Infant Bonding Scale) et validation comme évaluation du lien mère-nouveau-né en maternité.* . Devenir, 2017. **29**(4): p. 233-253.
26. Crane, D.R., K.C. Middleton, and R.A. Bean, *Establishing criterion scores for the Kansas marital satisfaction scale and the revised dyadic adjustment scale.* American Journal of Family Therapy, 2000. **28**(1): p. 53-60.
27. Haskett, M.E., et al., *Factor structure and validity of the parenting stress index-short form.* Journal of Clinical Child & Adolescent Psychology, 2006. **35**(2): p. 302-312.
28. Abidin, R.R., *Parenting stress index-short form*. 1990: Pediatric Psychology Press Charlottesville, VA.
29. Koch, F.-S., A. Sepa, and J. Ludvigsson, *Psychological stress and obesity.* The Journal Of Pediatrics, 2008. **153**(6): p. 839-844.
30. Foa, E.B., et al., *The validation of a self-report measure of posttraumatic stress disorder: the Posttraumatic Diagnostic Scale.* Psychological assessment, 1997. **9**(4): p. 445.

For peer review only

For peer review only - http://bmjopen.bmj.com/site/about/guidelines.xhtml

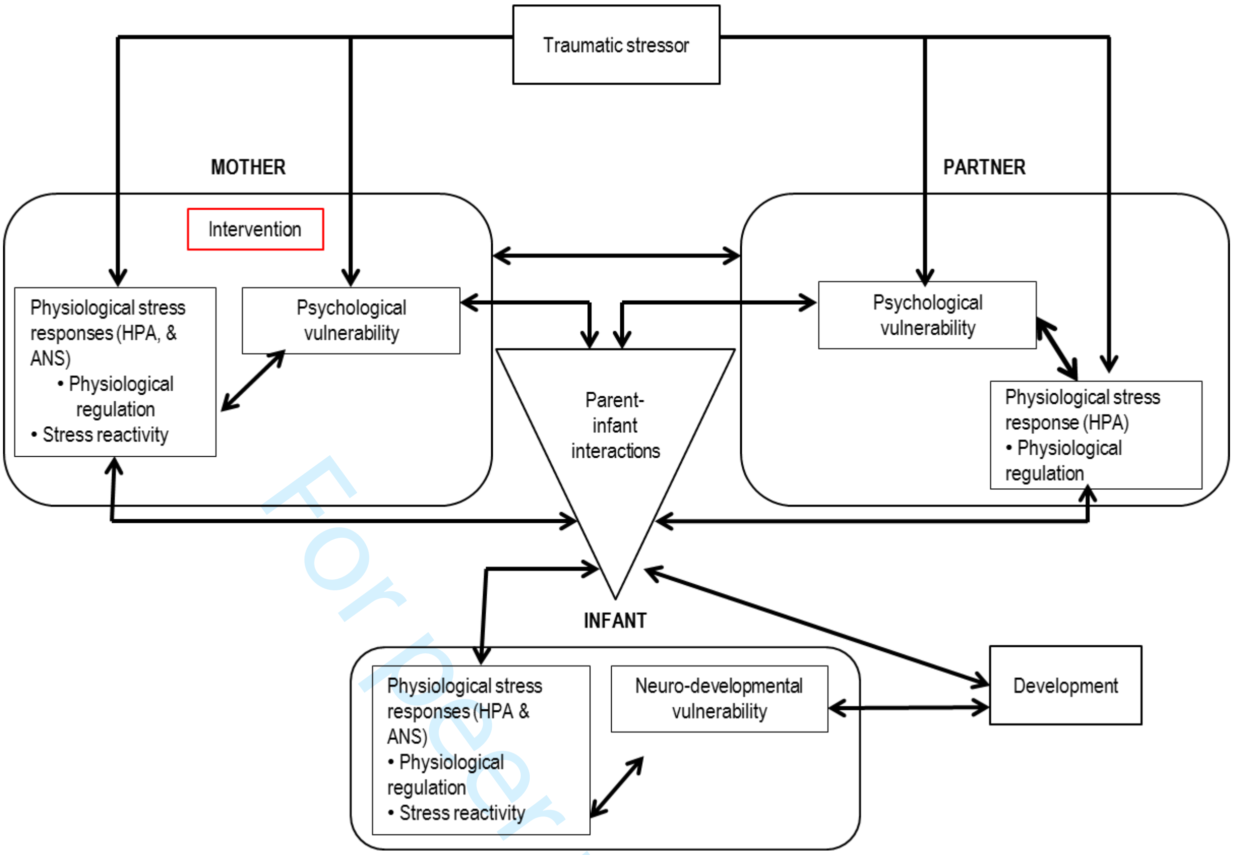

For peer review only

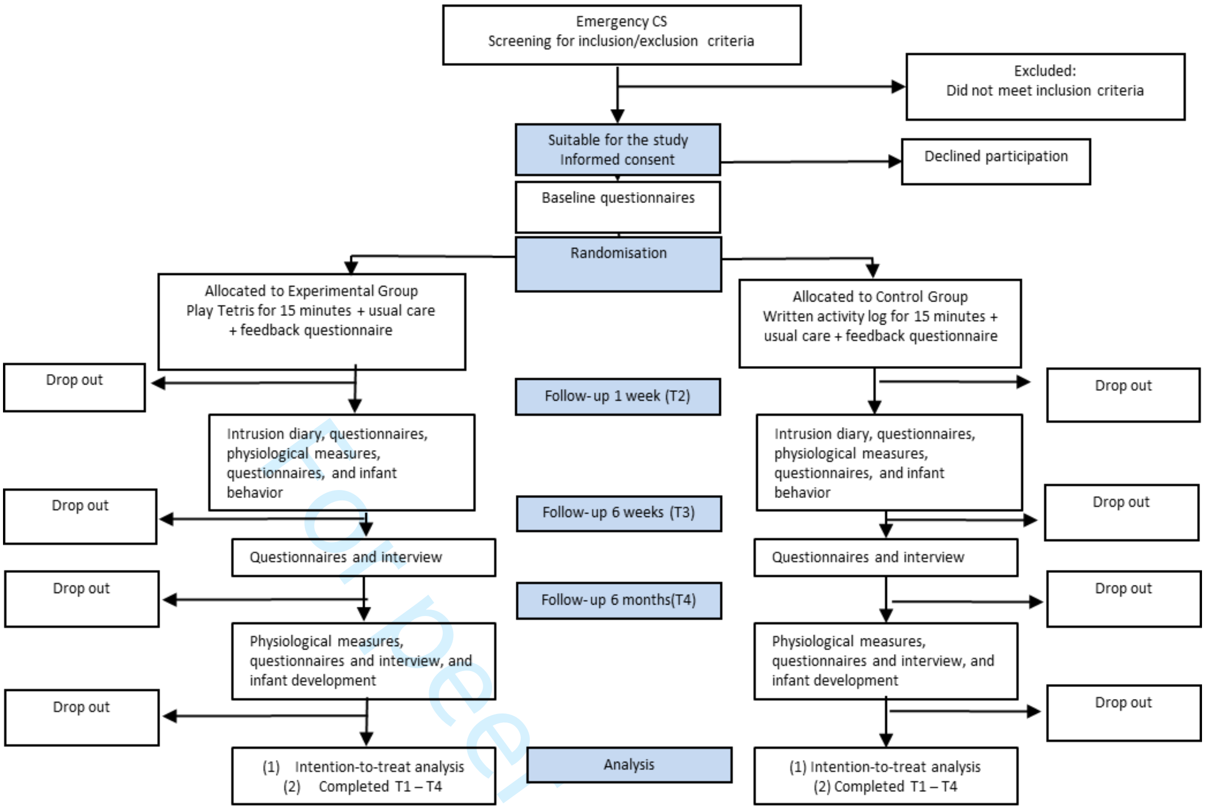
Emergency CS
Screening for inclusion/exclusion criteria
Excluded:
Did not meet inclusion criteria
Suitable for the study
Informed consent
Declined participation
Baseline questionnaires
Randomisation
Allocated to Experimental Group
Play Tetris for 15 minutes + usual care + feedback questionnaire
Allocated to Control Group
Written activity log for 15 minutes + usual care + feedback questionnaire
Drop out
Follow-up 1 week (T2)
Drop out
Intrusion diary, questionnaires, physiological measures, questionnaires, and infant behavior
Intrusion diary, questionnaires, physiological measures, questionnaires, and infant behavior
Drop out
Follow-up 6 weeks (T3)
Drop out
Questionnaires and interview
Questionnaires and interview
Drop out
Follow-up 6 months(T4)
Drop out
Physiological measures, questionnaires and interview, and infant development
Physiological measures, questionnaires and interview, and infant development
Drop out
Drop out
(1) Intention-to-treat analysis
(2) Completed T1 – T4
Analysis
(1) Intention-to-treat analysis
(2) Completed T1 – T4

STANDARD PROTOCOL ITEMS: RECOMMENDATIONS FOR INTERVENTIONAL TRIALS

SPIRIT 2013 Checklist: Recommended items to address in a clinical trial protocol and related documents*

Section/item	Item No	Description	Addressed on page number
**Administrative information**			
Title	1	Descriptive title identifying the study design, population, interventions, and, if applicable, trial acronym	1
Trial registration	2a	Trial identifier and registry name. If not yet registered, name of intended registry	6
	2b	All items from the World Health Organization Trial Registration Data Set	n/a
Protocol version	3	Date and version identifier	see original protocol submitted to ethics committee
Funding	4	Sources and types of financial, material, and other support	28
Roles and responsibilities	5a	Names, affiliations, and roles of protocol contributors	27
	5b	Name and contact information for the trial sponsor	1
	5c	Role of study sponsor and funders, if any, in study design; collection, management, analysis, and interpretation of data; writing of the report; and the decision to submit the report for publication, including whether they will have ultimate authority over any of these activities	27

For peer review only - http://bmjopen.bmj.com/site/about/guidelines.xhtml

	5d	Composition, roles, and responsibilities of the coordinating centre, steering committee, endpoint adjudication committee, data management team, and other individuals or groups overseeing the trial, if applicable (see Item 21a for data monitoring committee)	see original protocol submitted to ethics committee
**Introduction**			
Background and rationale	6a	Description of research question and justification for undertaking the trial, including summary of relevant studies (published and unpublished) examining benefits and harms for each intervention	8-14
	6b	Explanation for choice of comparators	16-17
Objectives	7	Specific objectives or hypotheses	14
Trial design	8	Description of trial design including type of trial (eg, parallel group, crossover, factorial, single group), allocation ratio, and framework (eg, superiority, equivalence, noninferiority, exploratory)	14
**Methods: Participants, interventions, and outcomes**			
Study setting	9	Description of study settings (eg, community clinic, academic hospital) and list of countries where data will be collected. Reference to where list of study sites can be obtained	14-15
Eligibility criteria	10	Inclusion and exclusion criteria for participants. If applicable, eligibility criteria for study centres and individuals who will perform the interventions (eg, surgeons, psychotherapists)	14-15
Interventions	11a	Interventions for each group with sufficient detail to allow replication, including how and when they will be administered	16-17
	11b	Criteria for discontinuing or modifying allocated interventions for a given trial participant (eg, drug dose change in response to harms, participant request, or improving/worsening disease)	n/a
	11c	Strategies to improve adherence to intervention protocols, and any procedures for monitoring adherence (eg, drug tablet return, laboratory tests)	see original protocol submitted to ethics committee

For peer review only - http://bmjopen.bmj.com/site/about/guidelines.xhtml

	11d	Relevant concomitant care and interventions that are permitted or prohibited during the trial	15
Outcomes	12	Primary, secondary, and other outcomes, including the specific measurement variable (eg, systolic blood pressure), analysis metric (eg, change from baseline, final value, time to event), method of aggregation (eg, median, proportion), and time point for each outcome. Explanation of the clinical relevance of chosen efficacy and harm outcomes is strongly recommended	17-22
Participant timeline	13	Time schedule of enrolment, interventions (including any run-ins and washouts), assessments, and visits for participants. A schematic diagram is highly recommended (see Figure)	Table 1<br>Figure 2
Sample size	14	Estimated number of participants needed to achieve study objectives and how it was determined, including clinical and statistical assumptions supporting any sample size calculations	23
Recruitment	15	Strategies for achieving adequate participant enrolment to reach target sample size	14-15
**Methods: Assignment of interventions (for controlled trials)**			
Allocation:			
Sequence generation	16a	Method of generating the allocation sequence (eg, computer-generated random numbers), and list of any factors for stratification. To reduce predictability of a random sequence, details of any planned restriction (eg, blocking) should be provided in a separate document that is unavailable to those who enrol participants or assign interventions	15
Allocation concealment mechanism	16b	Mechanism of implementing the allocation sequence (eg, central telephone; sequentially numbered, opaque, sealed envelopes), describing any steps to conceal the sequence until interventions are assigned	15
Implementation	16c	Who will generate the allocation sequence, who will enrol participants, and who will assign participants to interventions	15-16
Blinding (masking)	17a	Who will be blinded after assignment to interventions (eg, trial participants, care providers, outcome assessors, data analysts), and how	16
	17b	If blinded, circumstances under which unblinding is permissible, and procedure for revealing a participant’s allocated intervention during the trial	n/a

For peer review only - http://bmjopen.bmj.com/site/about/guidelines.xhtml

Methods: Data collection, management, and analysis

Data collection methods	18a	Plans for assessment and collection of outcome, baseline, and other trial data, including any related processes to promote data quality (eg, duplicate measurements, training of assessors) and a description of study instruments (eg, questionnaires, laboratory tests) along with their reliability and validity, if known. Reference to where data collection forms can be found, if not in the protocol	18-22
	18b	Plans to promote participant retention and complete follow-up, including list of any outcome data to be collected for participants who discontinue or deviate from intervention protocols	n/a
Data management	19	Plans for data entry, coding, security, and storage, including any related processes to promote data quality (eg, double data entry; range checks for data values). Reference to where details of data management procedures can be found, if not in the protocol	see original protocol submitted to ethics committee
Statistical methods	20a	Statistical methods for analysing primary and secondary outcomes. Reference to where other details of the statistical analysis plan can be found, if not in the protocol	24-25
	20b	Methods for any additional analyses (eg, subgroup and adjusted analyses)	23-24
	20c	Definition of analysis population relating to protocol non-adherence (eg, as randomised analysis), and any statistical methods to handle missing data (eg, multiple imputation)	23-24

Methods: Monitoring

Data monitoring	21a	Composition of data monitoring committee (DMC); summary of its role and reporting structure; statement of whether it is independent from the sponsor and competing interests; and reference to where further details about its charter can be found, if not in the protocol. Alternatively, an explanation of why a DMC is not needed	see original protocol submitted to ethics committee
	21b	Description of any interim analyses and stopping guidelines, including who will have access to these interim results and make the final decision to terminate the trial	n/a

Harms	22	Plans for collecting, assessing, reporting, and managing solicited and spontaneously reported adverse events and other unintended effects of trial interventions or trial conduct	see original protocol submitted to ethics committee
Auditing	23	Frequency and procedures for auditing trial conduct, if any, and whether the process will be independent from investigators and the sponsor	n/a
**Ethics and dissemination**			
Research ethics approval	24	Plans for seeking research ethics committee/institutional review board (REC/IRB) approval	24
Protocol amendments	25	Plans for communicating important protocol modifications (eg, changes to eligibility criteria, outcomes, analyses) to relevant parties (eg, investigators, REC/IRBs, trial participants, trial registries, journals, regulators)	see original protocol submitted to ethics committee
Consent or assent	26a	Who will obtain informed consent or assent from potential trial participants or authorised surrogates, and how (see Item 32)	24, see original protocol submitted to ethics committee
	26b	Additional consent provisions for collection and use of participant data and biological specimens in ancillary studies, if applicable	see original protocol submitted to ethics committee
Confidentiality	27	How personal information about potential and enrolled participants will be collected, shared, and maintained in order to protect confidentiality before, during, and after the trial	16, see original protocol submitted to ethics committee
Declaration of interests	28	Financial and other competing interests for principal investigators for the overall trial and each study site	28

For peer review only

Access to data	29	Statement of who will have access to the final trial dataset, and disclosure of contractual agreements that limit such access for investigators	see original protocol submitted to ethics committee
Ancillary and post-trial care	30	Provisions, if any, for ancillary and post-trial care, and for compensation to those who suffer harm from trial participation	n/a
Dissemination policy	31a	Plans for investigators and sponsor to communicate trial results to participants, healthcare professionals, the public, and other relevant groups (eg, via publication, reporting in results databases, or other data sharing arrangements), including any publication restrictions	24
	31b	Authorship eligibility guidelines and any intended use of professional writers	n/a
	31c	Plans, if any, for granting public access to the full protocol, participant-level dataset, and statistical code	n/a
**Appendices**			
Informed consent materials	32	Model consent form and other related documentation given to participants and authorised surrogates	upon request
Biological specimens	33	Plans for collection, laboratory evaluation, and storage of biological specimens for genetic or molecular analysis in the current trial and for future use in ancillary studies, if applicable	n/a

*It is strongly recommended that this checklist be read in conjunction with the SPIRIT 2013 Explanation & Elaboration for important clarification on the items. Amendments to the protocol should be tracked and dated. The SPIRIT checklist is copyrighted by the SPIRIT Group under the Creative Commons "Attribution-NonCommercial-NoDerivs 3.0 Unported" license.

For peer review only

BMJ Open

Improving mental health and physiological stress responses in mothers following traumatic childbirth and in their infants: Study protocol for the Swiss TrAumatic biRth Trial (START)

Journal:	*BMJ Open*
Manuscript ID	bmjopen-2019-032469.R1
Article Type:	Protocol
Date Submitted by the Author:	14-Oct-2019
Complete List of Authors:	Sandoz, Vania; University of Lausanne Faculty of Biology and Medicine, Institute of Higher Education and Research in Healthcare<br>Deforges, Camille; University of Lausanne Faculty of Biology and Medicine, Institute of Higher Education and Research in Healthcare<br>Stuijfzand, Suzannah; University of Lausanne Faculty of Biology and Medicine, Institute of Higher Education and Research in Healthcare<br>Epiney, Manuella; Geneva University Hospitals, Department Woman-Child-Adolescent<br>Vial, Yvan; University Hospital of Lausanne, 3Obstetrics and Gynecology Service, Woman-Mother-Child Department<br>Sekarski, Nicole; University Hospital of Lausanne, Paediatric Cardiology Unit, Woman-Mother-Child Department<br>Messerli, Nadine; University of Fribourg, Clinical Child Psychology & Biological Psychology<br>Ehlert, U; University of Zurich, Department of Clinical Psychology and Psychotherapy<br>Bickle-Graz, Myriam; University Hospital of Lausanne, Neonatology Service, Woman-Mother-Child Department<br>Morisod Harari, Mathilde; University Hospital of Lausanne, Service of Child and Adolescent Psychiatry,<br>Porcheret, Kate; Monash University, Turner Institute for Brain and Mental Health<br>Schechter, Daniel; University Hospital of Lausanne, Service of Child and Adolescent Psychiatry; University of Geneva Faculty of Medicine, Department of Psychiatry<br>Ayers, Susan; INTO City University London, Centre for Maternal and Child Health Research, School of Health Sciences<br>Holmes, Emily; Uppsala University, Department of Psychology<br>Horsch, Antje; University of Lausanne Faculty of Biology and Medicine, Institute of Higher Education and Research in Healthcare; University Hospital of Lausanne, Neonatology Service, Woman-Mother-Child Department,
**Primary Subject Heading**:	Mental health
Secondary Subject Heading:	Obstetrics and gynaecology, Evidence based practice, Reproductive medicine

Keywords:	early intervention, PTSD, maternal mental health, infant development, cortisol, HRV

SCHOLARONE™ Manuscripts

Improving mental health and physiological stress responses in mothers following traumatic childbirth and in their infants: Study protocol for the Swiss TrAumatic biRth Trial (START)

Vania Sandoz[1], Camille Deforges[1], Suzannah Stuijfzand[1], Manuella Epiney[2], Yvan Vial[3], Nicole Sekarski[4], Nadine Messerli, U Ehlert[6], Myriam Bickle-Graz[7], Mathilde Morisod Harari[8], Kate Porcheret[9], Daniel S. Schechter[10,11], Susan Ayers[12], Emily A. Holmes[13], & Antje Horsch[1,7*] on behalf of the *START Research Consortium*

[1]Institute of Higher Education and Research in Healthcare (IUFRS), University of Lausanne, Lausanne, Switzerland

[2]Department Woman-Child-Adolescent, Geneva University Hospital and University of Geneva, Geneva, Switzerland.

[3]Obstetrics and Gynecology Service, Woman-Mother-Child Department, Lausanne University Hospital and University of Lausanne, Lausanne, Switzerland

[4]Paediatric Cardiology Unit, Woman-Mother-Child Department, Lausanne University Hospital and University of Lausanne, Lausanne, Switzerland

[5]Clinical Child Psychology & Biological Psychology, University of Fribourg, Fribourg, Switzerland

[6]Department of Clinical Psychology and Psychotherapy, University of Zurich, Zurich, Switzerland

[7]Neonatology Service, Woman-Mother-Child Department, Lausanne University Hospital and University of Lausanne, Lausanne, Switzerland

[8]Service of Child and Adolescent Psychiatry, Lausanne University Hospital and University of Lausanne, Lausanne, Switzerland

For peer review only - http://bmjopen.bmj.com/site/about/guidelines.xhtml

[9]Turner Institute for Brain and Mental Health, Monash University, Australia

[10]University Hospital of Lausanne, Service of Child and Adolescent Psychiatry Lausanne, Lausanne, Switzerland

[11]Department of Psychiatry, University of Geneva Faculty of Medicine, Geneva, Switzerland

[12]Centre for Maternal and Child Health Research, School of Health Sciences, City, University of London, London, UK

[13]Department of Psychology, Uppsala University, Uppsala, Sweden

Email addresses:

Vania.Sandoz@bluewin.ch

Camille.Deforges@chuv.ch

Suzannah.Ravenscroft@chuv.ch

Manuella.Epiney@hcuge.ch

Yvan.Vial@chuv.ch

Nicole.Sekarski@chuv.ch

Nadine.Messerli@unifr.ch

U.Ehlert@psychologie.uzh.ch

Myriam.Bickle-Graz@chuv.ch

Mathilde.Morisod@chuv.ch

Kate.Porcheret@monash.edu

Daniel.Schechter@chuv.ch

Susan.Ayers.1@city.ac.uk

emily.holmes@psyk.uu.se

Antje.Horsch@chuv.ch

For peer review only

***Corresponding author:**

Prof. Antje Horsch, Institute of Higher Education and Research in Healthcare (IUFRS), University of Lausanne, SV-A, Route de la Corniche 10, 1010 Lausanne.

Phone: 00 41 21 314 85 71. Antje.Horsch@chuv.ch

KEYWORDS

early intervention, PTSD, maternal mental health, infant development, HRV, cortisol, sleep, Bayley-III, trauma, mental imagery, intrusive memories

For peer review only

ABSTRACT

Introduction

Emergency caesarean section (ECS) qualifies as a psychological trauma, which may result in postnatal posttraumatic stress disorder (PTSD). Maternal PTSD may not only have a significant negative impact on mother-infant interactions, but also on long-term infant development. The partner's mental health may also affect infant development. Evidence-based early interventions to prevent the development of postpartum PTSD in mothers are lacking. Immediately after a traumatic event, memory formation is vulnerable to interference. There is accumulating evidence that a brief behavioural intervention including a visuospatial task may result in a reduction in intrusive memories of the trauma.

Methods and analysis

This study protocol describes a double-blind multi-centre randomised controlled phase III trial testing an early brief maternal intervention including the computer game "Tetris" on intrusive memories of the ECS trauma (1 week) and PTSD symptoms (6 weeks, primary outcome) of 144 women following an ECS. The intervention group will carry out a brief behavioural procedure including playing Tetris. The attention-placebo control group will complete a brief written activity log. Both simple cognitive tasks will be completed within the first 6 hours following traumatic childbirth. The intervention is delivered by midwives/nurses in the maternity unit.

The primary outcome will be differences in the presence and severity of maternal PTSD symptoms between the intervention and the attention-placebo control group at 6 weeks postpartum. Secondary outcomes will be physiological stress and psychological vulnerability, mother-infant interaction, and infant developmental outcomes. Other outcomes will be

For peer review only - http://bmjopen.bmj.com/site/about/guidelines.xhtml

psychological vulnerability and physiological regulation of the partner and their bonding with the infant, as well as the number of intrusive memories of the event.

Ethics and dissemination

Ethical approval was granted by the Human Research Ethics Committee of the Canton de Vaud (study number 2017-02142). Dissemination of results will occur via national and international conferences, in peer-reviewed journals, public conferences, and social media.

Trial registration number

clinicaltrials.gov (NCT 03576586)

ARTICLE SUMMARY

Strengths and limitations of this study

- This multi-centre RCT will test the effects of an early brief behavioural intervention carried out by midwives/nurses on the labour ward.
- The primary outcome measure will be the presence and the severity of maternal PTSD symptoms at 6 weeks.
- The secondary outcomes will be the intrusive memories of the index traumatic event, maternal psychological vulnerability, maternal and child physiology, mother-baby interactions, and child developmental outcomes.
- The early brief intervention, conducted in the same hospital where the traumatic event occurred, includes a visuospatial task aimed at competing with sensory aspects of the traumatic memory before it has been fully consolidated.
- Methodological rigour, including a double-blind design, an active control group, concealment of random allocation, regular monitoring, and prospective trial registration, limits risk of bias.

For peer review only - http://bmjopen.bmj.com/site/about/guidelines.xhtml

ABBREVIATIONS

ANS: Autonomic Nervous System / ASD: Acute Stress Disorder / ASDS: Acute Stress Disorder Scale / BSID-III: Bayley Scales of Infant Development, 3rd Edition / CAPS: Clinician-Administered PTSD Scale for DSM-5 / CAR: Cortisol Awakening Response / CRIB: Clinical Risk Index for Babies / EAS: Emotional Availability Scales / ECG: Electrocardiography / ECS: Emergency Caesarean Section / EPDS: Edinburgh Postnatal Depression Scale / FFSF: Face-to-Face Still-Face paradigm / HADS: Hospital Anxiety and Depression Scale / HPA: Hypothalamic-Pituitary-Adrenal / HRV: Heart Rate Variability / IBIs: Interbeat Intervals / IBQ-R: Infant Behavior Questionnaire-Revised / MEQ: Morningness-Eveningness Questionnaire / MIBS: Mother-to-Infant Bonding Scale / MOS-8: Modified Medical Outcomes Study Social Support Survey / NBAS: Neonatal Behavioural Assessment Scale / PCL-5: PTSD Checklist for DSM-5 / PDPSI: Post-delivery Perceived Stress Inventory / PPSI: Postnatal Perceived Stress Inventory / PSI-SF: Parenting Stress Index - Short Form / PSQI: Pittsburgh Sleep Quality Index / PSQI-A: Pittsburgh Sleep Quality Index Addendum / PTSD: Post-Traumatic Stress Disorder / RCT: Randomised Controlled Trial / RDAS: Revised Dyadic Adjustment Scale / START: Swiss Traumatic Birth Trial

For peer review only

For peer review only - http://bmjopen.bmj.com/site/about/guidelines.xhtml

INTRODUCTION

Childbirth and PTSD

Though childbirth is a common and often fulfilling event, one third of mothers rate their childbirth as traumatic[1]. Childbirth can meet diagnostic criteria for a traumatic event, if women perceived their life and/or the life of their baby to be in danger[2]. Posttraumatic stress disorder (PTSD) related to childbirth is diagnosable in around 3-4% of women[3 4]. PTSD consists of four symptom clusters (intrusions, avoidance, hyperarousal, and negative cognitions and mood) and can be diagnosed at least one month after the traumatic stressor occurred[2]. Comparing different modes of childbirth, obstetric complications, such as emergency caesarean section (ECS) produce higher rates of postnatal PTSD (19-39%)[3]. ECS is a relatively frequent event, and there is thus a need to better identify and support women who are vulnerable to developing PTSD following an ECS.

Postnatal PTSD can significantly influence the experience of subsequent pregnancies, with increased risk of maternal stress and its associated risks of intrauterine growth retardation, premature birth and low birth weight[5-7]. It can lead to a fear of subsequent pregnancy and childbirth, sexual problems, and avoidance of medical care[8 9]. Postnatal PTSD can also have important negative consequences for breastfeeding, the attachment relationship with the baby and mother-infant interactions, with a subsequent detrimental impact on the development of the child, as well as for the couple relationship[7 10-13]. PTSD is also highly co-morbid with depression, for which there is substantial evidence of long-term negative effects on child development and behaviour[14-16]. Estimated economic costs of perinatal mental health problems are about £8.1 billion for each one-year cohort of UK births, of which 72% relate to adverse impacts on the child rather than the mother[17]. In Switzerland, 16.7% of women in the perinatal period used mental health services[18]. New and innovative evidence-based

For peer review only - http://bmjopen.bmj.com/site/about/guidelines.xhtml

interventions are therefore needed to reduce those costs by preventing the development of postnatal PTSD[19].

To date, research investigating PTSD symptoms in partners following ECS is missing. Most studies on partners so far have focused on postnatal depression reporting that 1-8% experience depression symptoms in the first 6 weeks and 5-6% at 3-6 months following childbirth without complications[20-22], with increased risk following high risk situations[23]. In one study, 5% of partners reported severe intrusions and avoidance symptoms at 9 weeks postpartum[24]. Although the influence of partner mental health is understudied, it also seems to negatively impact child outcomes[25-27]. Thus, partner mental health needs to be better understood not only in order to help the partner but also to support family and child outcomes.

Physiological stress responses associated with PTSD

Traumatic exposure activates the hypothalamic-pituitary-adrenal (HPA) axis, a cascade-like hormonal system resulting in the release of cortisol from adrenal cortex cells in body fluids. In parallel, the organism activates the more rapidly mobilizing autonomic nervous system (ANS) resulting in the release of norepinephrine from nerve terminals of the sympathetic nervous system as well as epinephrine and norepinephrine from the adrenal medulla[28]. While the HPA axis shows the above stress-related reactivity, it also shows a basal activity with circadian variations in the respective hormones. For instance, cortisol peaks 30-45 minutes after awakening (the so-called CAR: cortisol awakening response) and gradually declines throughout the day with lowest levels early during sleep[29 30].

Specific patterns of HPA axis functioning have been shown in PTSD[31] although this has so far not been studied after traumatic childbirth. While studies indicate that individuals with PTSD show different patterns of HPA axis functioning to those without PTSD, there is little consistency in the specificity of these patterns[32-38]. A meta-analysis examining diurnal

For peer review only - http://bmjopen.bmj.com/site/about/guidelines.xhtml

cortisol levels in adults with PTSD showed that low cortisol levels were not related to PTSD in general, but rather to trauma exposure and co-morbidities[39]. Finally, a recent study in a postnatal population found a negative association between symptoms of re-experiencing and diurnal cortisol slopes in mothers of preterm children[40]. Concerning cortisol reactivity to a subsequent stressor, again, results are discrepant[41 42].

Although HPA axis reactivity following stress or trauma is thought to be adaptive, acute or chronic exposure to stress has been shown to have deleterious effects[43 44]. It may not only result in dysfunctions of HPA re-activity, but also in health-relevant changes in the basal activation of this system[45]. Overall, these studies show the strong implication of the HPA axis dysregulation in the development and maintenance of PTSD, although studies in postnatal populations are scarce.

Reduced heart rate variability (HRV), an indicator of autonomic flexibility, has been found to be related to psychopathological processes[46]. Individuals with PTSD show lower levels of HRV in comparison with trauma-exposed individuals without PTSD or healthy controls[47 48]. However, the relationship of PTSD and HRV has so far not been studied in a postnatal population.

Sleep in PTSD is also disrupted. Sleep disturbance (i.e., difficulty falling or staying asleep) and recurrent distressing dreams are both diagnostic criteria for PTSD[2], with 70-91% of patients with PTSD suffering from subjective sleep disturbances and 19-71% reporting nightmares[49]. Findings from experimental research indicate that sleep on the first night after trauma may be important for the development of subsequent intrusive memories of the index trauma. One study found that totally sleep deprived participants reported *fewer* intrusive memories after a laboratory stressor compared to those who slept[50], though findings are mixed[51-53]. Thus, it is important to assess sleep over time following a real world traumatic

For peer review only

event, such as following an ECS. To date, no studies to our knowledge have examined sleep in postpartum PTSD.

Maternal PTSD and infant physiological stress responses

Maternal PTSD and its associated dysfunction of the HPA axis can also impact on the stress regulation of the offspring[54-63], such as the infant's HPA secretion patterns[64-66]. A growing body of neuroendocrine research supports the notion that an altered maternal HPA axis functioning plays a role in the intergenerational transmission of stress-related psychopathology from parents[67 68]. Overall, findings suggest that PTSD symptoms and cortisol levels in mothers are important to assess, prevent and/or treat as they may affect the relationship with the infant[69-72] and impact the child's later regulative abilities[54]. Some authors have suggested low maternal cortisol as a possible mechanism contributing to the mother's difficulty in sensitively attuning to her infant's cues, which in turn impacts on the infant's reactivity to and recovery from a stressor[73-78].

In contrast, studies assessing the role of ANS in the intergenerational transmission of stress in the postpartum period are so far scarce. Lifetime maternal psychopathology and maternal postnatal psychopathology have been found to be related with reduced HRV of their infants[79]. Furthermore, mothers with anxiety symptoms during pregnancy and their infants showed lower HRV[80] and there was a higher sympathetic activation in children of mothers with abuse histories[81]. However, to our knowledge none of the previous research has investigated the autonomic functioning in offspring of mothers with PTSD.

Developing an early intervention inspired by behavioural and cognitive neuroscience

To date, there is a lack of evidence-based early interventions for women following a traumatic childbirth[82]. At the heart of PTSD are intrusive memories of the traumatic event, in

For peer review only - http://bmjopen.bmj.com/site/about/guidelines.xhtml

which the person re-experiences aspects of the traumatic event, inflicting significant distress[2]. They have also been indicated as a precursor to the disorder[83]. Intrusive memories of trauma comprise sensory-perceptual images that are proposed to occur due to excessive perceptual (sensory) processing during a trauma[84].

The way in which individuals process a traumatic event influences their later intrusive memories of the trauma. Evidence from lab-based experiments have demonstrated that a brief behavioural intervention including a reminder cue, mental rotation and a visuospatial task (Tetris) can significantly reduce the frequency of intrusive images following exposure to traumatic film material[85 86]. One study showed that individuals who were instructed to engage in conceptually-driven processing, relative to those engaged in sensory-based, data-driven processing, reported more intrusive memories to a traumatic film[87]. It has been hypothesised that tasks which interfere with data-driven processing, such as sensory-perceptual, visuospatial tasks, may reduce the occurrence of intrusive memories of an index event[85 86]. Visuospatial cognitive tasks, such as the computer game Tetris, are thought to compete for resources with visuospatial images[88]. Studies of memory consolidation have shown that human memories are likely to still be malleable within 10 min to 6 hours, at which point the memory is thought to stabilise consolidation, making it more resistant to interference from a competing memory[89-91]. This indicates that such a working memory task may be most beneficial if delivered within approximately the first 6 hours following a traumatic event (see[92] for a review).

Two recent translational studies presented preliminary evidence for the efficacy of a brief intervention (including Tetris) in reducing the number of traumatic intrusive memories (over 1 week post-trauma) in patients arriving at an accident and emergency department (vs. attention placebo)[93] or in women in the first hours following ECS, the latter when compared with a treatment-as-usual control group[4]. In the latter study, per protocol analyses also showed

For peer review only - http://bmjopen.bmj.com/site/about/guidelines.xhtml

significantly lower re-experiencing symptoms at 1 week and lower rates of PTSD diagnosis at 1 month following ECS (secondary outcomes)[4].

Aims of the present study

The objectives of the present study are to investigate the effects of an early brief, behavioural intervention (including the computer game Tetris) delivered in the hospital context within 6 hours of the trauma, on maternal mental health and infant development after a traumatic event (ECS). The primary outcome measure will be the presence and the severity of maternal PTSD symptoms at 6 weeks. Secondary objectives will be to measure the impact of this intervention on intrusive memories of the trauma, on stress exposure and perception, on other indicators of maternal psychological vulnerability (including acute stress disorder (ASD), PTSD, anxiety, depression, and sleep), on physiological stress reactivity, on physiological regulation, on mother-infant interactions, and on infant development. The START study also aims to investigate additional maternal and partner psychological vulnerability, partner physiological regulation, partner-infant bonding, and measures related to the acceptability and expectancy of the intervention.

METHODS

Study Design

We will conduct a multi-centre double-blind randomised controlled trial (RCT) with minimal risk testing the effect of an early brief behavioural intervention including computer game play for women at risk of PTSD in the hospital soon following an ECS and their infant, compared with an attention-placebo control task.

For peer review only - http://bmjopen.bmj.com/site/about/guidelines.xhtml

Study population, recruitment, group allocation, and blinding

All women who have an ECS ≥34 weeks gestation, give birth to a live baby, and give written consent are eligible to participate. In addition, they have to answer with a score of ≥2 separately for at least two out of four screening questions regarding perceived threat[94]. All screening questions are answered on a 7-point Likert scale (*1 = not at all, 7 = extremely*): Did you think that your life was in danger? Did you think that your baby's life was in danger? Did you feel frightened during the birth? Did you feel helpless during the birth?

Exclusion criteria include: established intellectual disability or psychotic illness, insufficient French-speaking level to participate in assessments, severe illness of mother or infant (e.g., cancer, cardiovascular disease, severe neurodevelopmental difficulties, malformations) or if the infant requires intensive care, and alcohol abuse and/or illegal drug use during pregnancy.

Following an ECS and once the mother has sufficiently recovered, the mother's midwife/nurse will assess eligibility and if eligible, will inform the mother about the study. After providing written and informed consent to be screened, participants will be screened immediately for perceived threat of the mother and/or child with four screening questions. If participants score ≥2 separately for at least two out of the four questions, they will be randomly assigned to either the intervention or attention-placebo control group. We aim to recruit 144 women and their infants (see sample size calculation).

If the woman agrees, then the partner will also be informed about the study. Inclusion criteria for the partners are that they were present at the childbirth and give written consent. Partners are excluded if they do not speak French sufficiently well to participate in assessments. All participants will be reimbursed for their time and effort.

The allocation ratio of randomisation is 1:1. The randomization sequence will be generated using a computer-generated block randomisation (StataCorp. 2017. *Stata Statistical*

For peer review only - http://bmjopen.bmj.com/site/about/guidelines.xhtml

Software: Release 15. College Station, TX) using blocks of sizes 2, 4 and 6 over 144 participants per stratum (stratified by research centre). Opaque envelopes will be prepared in advance, numbered sequentially by alternating between stratums by block. After conducting the baseline assessment, the clinical midwife/nurse will open the envelope and announce the cognitive task to be carried out (i.e., the cognitive visuospatial task or the attention-placebo control task) to the participant[95]. All members of the research group as well as participants are blind to group allocation. All participant data will be coded to ensure confidentiality. After completing the 15-minute cognitive tasks, both women of the control and intervention group will complete the same assessments, as shown in Table 1; see Figure 1 for an overview of the study variables.

Intervention group

Mothers in the intervention group will be instructed to engage in a cognitive visuospatial task, the computer game Tetris, for 15 minutes continuously, on a handheld gaming device (Nintendo DS) (see[4]). They are given a 3-min training in how to play the game and how to actively use mental rotation as they play the game. The intervention is delivered in the same context as that in which the trauma occurred (e.g,, wake-up room) so additional memory reminder cue to the trauma is not used.

The intervention will take place within the first six hours after ECS whilst participants are still in their hospital bed. Procedures are managed not to interfere with important routine care procedures. An unblinded independent researcher will check during the days following the intervention that the intervention protocol was followed correctly via a 4-item survey completed by the participant. Study information and materials refer to 'simple tasks' in both conditions for credibility.

For peer review only - http://bmjopen.bmj.com/site/about/guidelines.xhtml

Attention-placebo control group

Mothers assigned to the control group will be asked to engage in a written activity log for 15 minutes. The activity log was selected to control for nonspecific confounding factors, while minimizing the potential for harmful effects, as to date, no preventive treatment in the immediate aftermath of trauma exists that could be used as a control condition[96-99]. This control condition was matched with the intervention condition for nonspecific factors including length of the task, contact with the midwife/nurse, location of treatment procedure and engagement in a structured task[93]. The attention-placebo control task will take place within the six hours after ECS, whilst participants are still in their hospital bed and will not interfere with important routine care procedures. An unblinded independent researcher will check during the days following childbirth that the attention-placebo control task protocol was followed correctly via a 2-item survey completed by the participant.

Study information and materials refer to 'simple tasks' in both conditions for credibility.

Primary outcome

The primary outcomes are differences in the presence and severity of maternal PTSD symptoms between the intervention and the attention-placebo control group at 6 weeks postpartum measured with the Clinician-administered PTSD Scale[100] and the PTSD Checklist[101].

Secondary outcomes

The following outcomes will be assessed via validated assessments at different time-points (i.e., ≥6 hours following ECS, ≤1 and 6 weeks, and 6 months follow-up).

Maternal outcomes

For peer review only - http://bmjopen.bmj.com/site/about/guidelines.xhtml

The maternal mental health outcomes will be compared between the two groups at all time-points, including number of intrusive memories of the index trauma and indicators of maternal psychological vulnerability namely: symptoms of ASD, PTSD, anxiety, depression, sleep and physical activity (see Table 1). Additional physiological outcomes will be collected in reactivity to stress and as regulation indicators. Finally, maternal bonding and sensitivity in mother-infant interaction will also be measured.

Child outcomes

As shown in Table 1, infant development will be assessed at 6 months postpartum. Additionally, physiological outcomes will be assessed in response to stress and as regulation indicators.

Other outcomes

Additional measures of maternal and partner psychological vulnerability, partner infant interaction, infant neurodevelopmental vulnerability, medical outcomes, and measures related to the acceptability and expectancy of the intervention are described in Table 2.

Data collection and visits

Figure 2 summarises study procedures and Table 1 indicates the measures collected at each time point.

Measures

Measures of the primary and secondary outcomes can be found below, measures relating to the 'other outcomes' in the study can be found in Table 2. The time points of when all measures are taken can be seen in Table 1.

For peer review only - http://bmjopen.bmj.com/site/about/guidelines.xhtml

Psychological Vulnerability

Clinician-administered PTSD Scale (CAPS-5)[100]. The CAPS-5 assesses presence and severity of PTSD symptoms and diagnosis. This gold standard instrument contains 20 items referring to the four symptom clusters, as well as 10 items referring to symptoms duration, distress or impairment, global ratings, and dissociative subtype. Each diagnostic criterion is rated from *0=absent* to *4=extreme/incapacity* in function of symptoms intensity and frequency with a diagnostic cut-off equal to 2. The CAPS-5 has demonstrated good psychometric proprieties[100]. In the absence of a French version at the time when the study was designed, a forward-back method was executed to realise a translation and cultural adaptation[102].

The PTSD Checklist (PCL-5)[101]. This 20-item self-report questionnaire measures symptoms of PTSD over the past month and is used to assess frequency of PTSD symptoms. The PCL-5 refers to the four symptoms clusters of PTSD and scales on a 5-point scale with *0=not at all* and *4=extremely*. Scores are summed to create a total symptom severity score [103]. The French version of the PCL-5 demonstrated strong reliability and validity[104]**.**

Trauma-related intrusive memories diary[4]. Intrusive memories of birth-related trauma experienced during the seven days following ECS are reported in a daily diary, adapted from previous work[4], to assess the frequency of intrusive memories of the trauma . For each intrusion, the time, content and type (intrusive memory, nightmare or other) are recorded, as well as the level of distress on a 5-point scale with ratings of *0=not at all* to *5=extremely.*

Acute Stress Disorder Scale (ASDS)[105]. This self-assessment instrument measures frequency of ASD symptoms over the last week and is based on DSM 5[106]. Each of the 19 items is scored using a scale from *1=not at all* to *5=extremely*, with a higher score indicating

For peer review only - http://bmjopen.bmj.com/site/about/guidelines.xhtml

higher ASD symptoms. Good sensitivity and specificity has been reported[105]. The forward-back method was executed to realise a French version translation and cultural adaptation[102].

Anxiety subscale of Hospital Anxiety and Depression Scale (HADS)[107]. This self-report questionnaire measures severity of anxiety symptoms during the last week. The anxiety subscale consists of 7 items scored on a 4-point scale (*0=never*, *3=most of the time*). Higher scores indicate higher distress. Good psychometric properties have been reported for the French version[108].

Edinburgh Postnatal Depression Scale (EPDS)[109]. This self-assessment examines postnatal depression symptoms over the previous week[109]. The 10 items are scored on a 4-point scale and scores range from 0 to 30. Higher scores indicate higher distress. The French version has demonstrated good psychometric proprieties[110]. A clinical cut-off score of 10.5 has been reported for the use of the French validated version[110].

Physical and sleep activity. Frequency and duration of maternal sleep and physical activity of the 5 days following childbirth is measured using an accelerometer watch GENEActiv®[111].

Sleep diary. Mothers record their hours of sleep during the week following ECS in a daily sleep diary.

Pittsburgh Sleep Quality Index (PSQI)[112]. This 19-item questionnaire measuring sleep during the past month is composed of seven sleep quality-related subscales. The overall score assessing sleep quality is scored by summing the subscales; scores range from 0 to 21. Higher results indicate poor sleep quality while a score of >5 distinguishes good and poor sleepers. The validated French version of the PSQI has shown good psychometrics proprieties[113].

We also included the 10 items of the PSQI-A[114] to assess PTSD-specific sleep disturbances over the past month, answered on a four-point Likert scale (0=*not during the past month*; 3=*three or more times a week*), that assess the frequency of different kinds of

For peer review only - http://bmjopen.bmj.com/site/about/guidelines.xhtml

trauma-related sleep disturbance. The items are summed to create the total score, where the higher the score the more disturbed the sleep. The validated French version of the PSQI-A has shown good psychometrics[114].

Maternal and infant physiological stress responses.

Physiological regulation. Resting heart rate of the mother and infant are assessed using resting HRV measured by Firstbeat Bodyguard 2 devices providing a continuous measure of cardiac activity. Resting heart rate will be assessed during the 15 minutes resting period before the stress paradigms (see *Physiological stress reactivity* for stress paradigms) at ≤1week and 6 months. Baseline salivary cortisol and cortisol daily profile will be established for the mother and her infant in the two days after leaving the maternity ward (usually the 6th and 7th day postpartum) and for two days at 6 months through salivary sample; 5 saliva samples are taken per day, including CAR. Maternal salivary cortisol will be collected using Salivettes© Sarstedt (item number: 51.1534.500) and SalivaBio Infant's Swab (Salimetrics, item number: 5001.08 50) for infants.

Physiological stress reactivity. Maternal and infant stress reactivity will be assessed via salivary cortisol and HRV using Firstbeat Bodyguard 2 devices during the stress phases of their respective stress paradigms at 6 months for the mother and at ≤1week and at 6 months for the infant. The stress paradigm for the mothers is the Trier Social Stress Test (TSST)[115] at 6 months. Maternal salivary cortisol will be measured 7 times before, during, and after the stress paradigms and heart rate throughout the stress paradigm. Stress paradigms for the infants involve the Neonatal Behavioural Assessment Scale (NBAS)[116] at ≤1 week and the double-exposure Face-to-Face Still-Face paradigm (FFSF)[117-120] at 6 months. Salivary cortisol will be measured 3 times, once before and twice after the stressor, and HRV will be measured throughout the stress paradigms. Maternal cortisol will also be collected during the FFSF.

For peer review only - http://bmjopen.bmj.com/site/about/guidelines.xhtml

Maternal salivary cortisol will be collected using Salivettes© Sarstedt and SalivaBio Infant's Swab (Salimetrics) for infants. Participation in maternal stress reactivity assessments at ≤1week is optional (involving an additional consent obtained before hospital discharge).

Mother-infant interaction

Mother-to-Infant Bonding Scale (MIBS)[121]. This 8-item questionnaire assesses the mothers' feelings towards her new baby in the first few weeks after birth. Eight adjectives are rated on a scale from *0=very much* to *5=not at all*. Scores are summed to create a total score, with a higher score indicating worse mother-to infant bonding. The MIBS has shown good initial psychometrics[121], and has been validated in French[122].

Emotional Availability Scale (EAS)[123 124]. Maternal sensitivity and responsiveness will be investigated during a free-play session of mothers with their six-months old. Interactions are coded on six dimensions: sensitivity, structuration, intrusion, hostility toward the infant, reactivity to the mother, and maternal involvement[123 124]. Higher scores on each scale indicate better performance. Two trained psychologists blind to the condition will rate each interaction and inter-observer reliability will be calculated. The EAS shows good psychometric properties[123 124].

Infant developmental outcomes.

Infant Behaviour Questionnaire – Revised (IBQ-R) Very Short Form[125]. This is a parent-report questionnaire consisting of 36 items answered on a 7-point Likert-scale (*1=never* to *7=always*). The items assess the frequency of infants' behaviours during the previous two weeks to measure child temperament. The very short form has shown good psychometric properties[125]. There was no available validation French translation. Therefore,

For peer review only - http://bmjopen.bmj.com/site/about/guidelines.xhtml

the forward-back method was completed to realise a French version translation and cultural adaptation[102]

Bayley Scales of Infant Development (Bayley-lll)[126]. The cognitive, language and motor subscales of the Bayley Scales of Infant Development assess the developmental functioning of the infant. The scales are administered by a trained psychologist or paediatrician through a standard set of play tasks following a standardized protocol. The composite scores for the subscales are age-standardized with a mean score of 100[127].

Sociodemographic, obstetric and neonatal characteristics

Mothers will report demographic information, including marital status, nationality, profession, level of education[128], and previous and current psychiatric disease as well as any trauma history via a self-report questionnaire. Mothers will also report their height, weight (before pregnancy and current), menstrual cycle, smoking behaviours, and alcohol/drug use. Obstetric data will be extracted from the hospital medical record, such as pregnancy-, labour- and birth-related information, gravidity, parity, mode of previous childbirths, history of miscarriage, stillbirth, and prematurity, pain, medication, birth control, sexual activity, and psychological support. Neonatal characteristics will be collected from the medical record on severity of morbidity (gestational age and weight at birth, Apgar score, neonatal complications), as well as the Clinical Risk Index for Babies (CRIB II)[129], which represents neonatal morbidity severity.

Sample size calculation

Based on a previous proof-of-principle RCT[4], a sample size of n=120 participants is necessary to have 80% power (α=0.05) to detect a between-group difference of d=0.30 of the primary outcome (PTSD symptoms at 6 weeks). Furthermore, we calculated the sample sizes

For peer review only - http://bmjopen.bmj.com/site/about/guidelines.xhtml

necessary to obtain significant group differences with 80% power (α=0.05) based on small effect sizes (d<0.30) for maternal secondary outcomes (PTSD diagnosis, ASD symptoms and physiological stress reactivity) and infant secondary outcomes (physiological stress reactivity) based on small effect sizes (d<0.30) with 80% power (α=0.05), which range between a total of n=56 and n=84. Predicting a 20% drop-out rate, we aim to recruit 144 women.

PATIENT AND PUBLIC INVOLVEMENT

The acceptability of the intervention was assessed by participants during the previous proof-of-principle RCT[4]. A questionnaire of satisfaction and acceptability examined the burden of the intervention. An individual feedback and debriefing session will be offered to each participant. Results will be disseminated in written form to the participants and distributed to the public via social media and public events. They will also be discussed with clinical professionals involved in this project.

DATA MANAGEMENT AND STATISTICAL ANALYSES

All study data will be entered by research staff. Study data will be managed using REDCap electronic data capture tools hosted at Lausanne University Hospital[130 131]. REDCap (Research Electronic Data Capture) is a secure, web-based software platform designed to support data capture for research studies. Double data entry will be done for the primary outcomes. For the rest of the data, a random 5% will be double-checked. Access to the full final trial dataset will be restricted to the principal investigator.

For the primary analyses, group differences regarding the mean subscale and total scores of the PCL-5 and CAPS-5 at 6 weeks will be analysed using linear regression analysis. Analyses will be adjusted for recruitment centre and the respective baseline values. Associations between primary outcomes and potential confounders, such as maternal and

For peer review only - http://bmjopen.bmj.com/site/about/guidelines.xhtml

gestational age, will first be assessed applying univariate tests. Subsequent analyses will be adjusted for significant covariates. For secondary aims, the analyses will be performed for differences in changes between groups and differences between groups at different time points in maternal and infant outcomes. Proportion of participants meeting the diagnostic criteria for PTSD at 6 weeks and 6 months between the two groups will also be compared using logistic regression analyses. The same procedure for identifying significant covariates described above will be applied here. Furthermore, post-hoc exploratory analyses will be conducted but described as such in publications. All regression analyses regarding cortisol data will be adjusted for potential covariates, such as co-sleeping, breastfeeding, menstrual cycle, and infant and maternal medication. For HRV analyses interbeat intervals (IBIs) from baseline, stress task and recovery will be analysed by the extraction from ECG recordings. Statistical parameters of HRV[132] will be calculated using Kubios HRV Analysis2.2 software[133]. Time domain measures and spectral frequency measures will be used for calculations. Multiple imputation methods will be used to manage missing data, if appropriate.

ETHICAL APPROVAL

This study protocol was approved by the Human Research Ethics Committee of the Canton de Vaud (Switzerland, study number 2017-02142, protocol version 5 of September 13rd 2019) and is registered in *clinicaltrials.gov* (NCT03576586). Substantial amendments will only be implemented after approval of the ethics committee, and all non-substantial amendments are communicated to the ethics committee within the Annual Safety Report. Participating mothers and, where included, partners will provide their written informed consent before their enrolment in the study by the clinical and research staff. The written informed consent contains an optional part on the re-use of data in ancillary studies.

For peer review only - http://bmjopen.bmj.com/site/about/guidelines.xhtml

Participation will not interfere with typical care provided to patients after an ECS. The scientific conduct of the study is overseen by an interdisciplinary steering committee of national and international experts. Confidentiality will be ensured by utilising subject identification code numbers to correspond to treatment data in the computer files.

ACKNOWLEDGMENTS

We thank Pierluigi Ballabeni for his statistical advice and the generation of the randomization sequence. We also thank our interns for help with study co-ordination and data collection: Lara Schütz, Anna Favero, Yasmina Lotfi, Mathilde Duroux, Cassie Pernet, Daniela Barros Rodrigues, Mélanie Hausser, and Annabelle Reuse. For institutional support, access to the study population, help with recruitment, data collection, and administrative support, we acknowledge Laetitia Accrombessi, Danièle Losguardi, Pascale Gerdy, Maria-Teresa Adjaho, Lauretta Monney, Nadia Carota, Annette Schauenberg, Laetitia Favre, Antonina Chilin, Céline Favrod, and all clinical teams of the labor and postpartum wards at the Lausanne and Geneva University Hospitals.

COLLABORATORS

The following are members of the *START Research Consortium* (including the authors of the present article), listed in alphabetical order:

- Valérie Avignon (Woman-Mother-Child Department, Lausanne University Hospital and University of Lausanne, Lausanne, Switzerland; valerie.avignon@chuv.ch),
- Susan Ayers (Centre for Maternal and Child Health Research, School of Health Sciences, City, University of London, London, UK; susan.ayers.1@city.ac.uk),

For peer review only - http://bmjopen.bmj.com/site/about/guidelines.xhtml

- Myriam Bickle-Graz (Neonatology Service, Woman-Mother-Child Department, Lausanne University Hospital and University of Lausanne, Lausanne, Switzerland; myriam.bickle-graz@chuv.ch),
- Cristina Borradori Tolsa (Department of Pediatrics, Geneva University Hospitals, Geneva, Switzerland; cristina.borradoritolsa@hcuge.ch),
- Julie Bourdin (Woman-Mother-Child Department, Lausanne University Hospital and University of Lausanne, Lausanne, Switzerland; julie.bourdin@chuv.ch),
- Camille Deforges (Institute of Higher Education and Research in Healthcare (IUFRS), University of Lausanne, Lausanne, Switzerland; camille.deforges@chuv.ch),
- Dominique Delecraz (Department of Woman, Child and Adolescent, Geneva University Hospitals, Geneva, Switzerland; dominique.delecraz@hcuge.ch),
- David Desseauve (Woman-Mother-Child Department, Lausanne University Hospital and University of Lausanne, Lausanne, Switzerland; david.desseauve@chuv.ch),
- Ulrike Ehlert (Department of Clinical Psychology and Psychotherapy, University of Zurich, Zurich, Switzerland; u.ehlert@psychologie.uzh.ch),
- Manuella Epiney (Department of Woman, Child and Adolescent, Geneva University Hospitals, Geneva, Switzerland; manuella.epiney@hcuge.ch),
- Isabelle Eragne (Department of Woman, Child and Adolescent, Geneva University Hospitals, Geneva, Switzerland; Isabelle.Eragne@hcuge.ch),
- Emily A. Holmes (Department of Psychology, Uppsala University, Uppsala, Sweden; emily.holmes@psyk.uu.se),
- Antje Horsch (Institute of Higher Education and Research in Healthcare (IUFRS), University of Lausanne and Neonatology Service, Woman-Mother-Child Department, Lausanne University Hospital and University of Lausanne, Lausanne, Switzerland; antje.horsch@chuv.ch),

For peer review only

For peer review only - http://bmjopen.bmj.com/site/about/guidelines.xhtml

- Justine Imbert (Woman-Mother-Child Department, Lausanne University Hospital and University of Lausanne, Lausanne, Switzerland; justine.imbert@chuv.ch),
- Nadine Messerli-Bürgy (Clinical Child Psychology & Biological Psychology, University of Fribourg, Fribourg, Switzerland; nadine.messerli@unifr.ch),
- Micah M. Murray (Faculty of Biology and Medicine, University of Lausanne, Lausanne, Switzerland; Micah.Murray@chuv.ch),
- Mathilde Morisod Harari (Service of Child and Adolescent Psychiatry, Lausanne University Hospital and University of Lausanne, Lausanne, Switzerland; mathilde.morisod@chuv.ch),
- Kate Porcheret (Turner Institute for Brain and Mental Health, Monash University, Monash University, Melbourne, Australia; kate.porcheret@monash.edu),
- Jardena J. Puder (Woman-Mother-Child Department, Lausanne University Hospital and University of Lausanne, Lausanne, Switzerland; jardena.puder@chuv.ch),
- Véronique Othenin-Girard (Department of Woman, Child and Adolescent, Geneva University Hospitals, Geneva, Switzerland; veronique.othenin-girard@hcuge.ch),
- Suzannah Stuijfzand (Institute of Higher Education and Research in Healthcare (IUFRS), University of Lausanne, Lausanne, Switzerland; suzannah.ravenscroft@chuv.ch),
- Vania Sandoz, M.Sc. (Institute of Higher Education and Research in Healthcare (IUFRS), University of Lausanne, Lausanne, Switzerland; vania.sandoz@chuv.ch),
- Daniel S. Schechter (Service of Child and Adolescent Psychiatry, Lausanne University Hospital and University of Lausanne, Lausanne, Switzerland, and Department of Psychiatry, University of Geneva Faculty of Medicine, Geneva, daniel.schechter@hcuge.ch),

For peer review only - http://bmjopen.bmj.com/site/about/guidelines.xhtml

- Nicole Sekarski (Paediatric Cardiology Unit, Woman-Mother-Child Department, Lausanne University Hospital and University of Lausanne, Lausanne, Switzerland; nicole.sekarski@chuv.ch),
- Jean-François Tolsa (Department of Woman-Mother-Child, Clinic of Neonatology, Lausanne University Hospital and University of Lausanne, Lausanne, Switzerland; jean-francois.tolsa@chuv.ch),
- Yvan Vial (Obstetrics and Gynecology Service, Woman-Mother-Child Department, Lausanne University Hospital and University of Lausanne, Lausanne, Switzerland; yvan.vial@chuv.ch).

AUTHOR CONTRIBUTIONS

AH designed the study with input from all co-authors and members of the consortium. VS, ME, YV, NS, NMB, UE, MBG, MMH, KP, DS, SA and EH participated in the design of the study. AH, VS, CD and SR drafted the manuscript. VS, CD, ME, NMB, MBG, SR, EH and AH significantly contributed to the establishment and refinement of study procedures. All authors critically revised the manuscript and approved the final version of the manuscript.

FUNDING

The START study is funded by a project grant from the Swiss National Science Foundation (SNF 32003B_172982/1).

COMPETING INTERESTS

None declared.

ETHICAL APPROVAL

For peer review only - http://bmjopen.bmj.com/site/about/guidelines.xhtml

Ethical approval was granted by the Human Research Ethics Committee of the Canton de Vaud (study number 2017-02142).

REFERENCES

1. Soet JE, Brack GA, DiIorio C. Prevalence and Predictors of Women's Experience of Psychological Trauma During Childbirth. *Birth* 2003;30(1):36-46. doi: 10.1046/j.1523-536X.2003.00215.x
2. American Psychiatric Association. Diagnostic and statistical manual of mental disorders. 5 ed. Arlington, VA: American Psychiatric Publishing 2013.
3. Yildiz PD, Ayers S, Phillips L. The prevalence of posttraumatic stress disorder in pregnancy and after birth: A systematic review and meta-analysis. *Journal of Affective Disorders* 2017;208:634-45. doi: http://dx.doi.org/10.1016/j.jad.2016.10.009
4. Horsch A, Vial Y, Favrod C, et al. Reducing intrusive traumatic memories after emergency caesarean section: A proof-of-principle randomized controlled study. *Behaviour Research and Therapy* 2017;94:36-47. doi: http://doi.org/10.1016/j.brat.2017.03.018
5. Rogal SS, Poschman K, Belanger K, et al. Effects of posttraumatic stress disorder on pregnancy outcomes. *Journal of affective disorders* 2007;102(1-3):137-43. doi: https://doi.org/10.1016/j.jad.2007.01.003
6. Seng JS, Oakley DJ, Sampselle CM, et al. Posttraumatic stress disorder and pregnancy complications. *Obstetrics & Gynecology* 2001;97(1):17-22. doi: https://doi.org/10.1097/00006250-200101000-00004
7. Cook N, Ayers S, Horsch A. Maternal posttraumatic stress disorder during the perinatal period and child outcomes: A systematic review. *Journal of affective disorders* 2018;225:18-31. doi: https://doi.org/10.1016/j.jad.2017.07.045
8. Hofberg K, Brockington I. Tokophobia: an unreasoning dread of childbirth. *The British journal of psychiatry : the journal of mental science* 2000;176(1):83-85. doi: https://doi.org/10.1192/bjp.176.1.83 [published Online First: 2000/05/02]
9. Morland L, Goebert D, Onoye J, et al. Posttraumatic stress disorder and pregnancy health: preliminary update and implications. *Psychosomatics* 2007;48(4):304-8. doi: 10.1176/appi.psy.48.4.304 [published Online First: 2007/06/30]
10. Garthus-Niegel S, Horsch A, Ayers S, et al. The influence of postpartum PTSD on breastfeeding: A longitudinal population-based study. *Birth* 2018;45(2):193-201. doi: https://doi.org/10.1111/birt.12328
11. Garthus-Niegel S, Horsch A, Bickle Graz M, et al. The prospective relationship between postpartum PTSD and child sleep: A 2-year follow-up study. *Journal of affective disorders* 2018;241:71-79. doi: https://doi.org/10.1016/j.jad.2018.07.067
12. Garthus-Niegel S, Horsch A, Handtke E, et al. The Impact of Postpartum Posttraumatic Stress and Depression Symptoms on Couples' Relationship Satisfaction: A Population-Based Prospective Study. *Frontiers in Psychology* 2018;9:1728. doi: https://doi.org/10.3389/fpsyg.2018.01728
13. Pisoni C, Spairani S, Fauci F, et al. Effect of maternal psychopathology on neurodevelopmental outcome and quality of the dyadic relationship in preterm infants: an explorative study. *The Journal of Maternal-Fetal & Neonatal Medicine* 2018:1-10. doi: https://doi.org/10.1080/14767058.2018.1487935
14. Grace SL, Evindar A, Stewart D. The effect of postpartum depression on child cognitive development and behavior: a review and critical analysis of the literature. *Archives of*

For peer review only - http://bmjopen.bmj.com/site/about/guidelines.xhtml

Women's Mental Health 2003;6(4):263-74. doi: https://doi.org/10.1007/s00737-003-0024-6
15. Pearson RM, Bornstein MH, Cordero M, et al. Maternal perinatal mental health and offspring academic achievement at age 16: the mediating role of childhood executive function. *Journal of Child Psychology and Psychiatry* 2016;57(4):491-501. doi: https://doi.org/10.1111/jcpp.12483
16. Sandoz V, Bickle-Graz M, Camos V, et al. Maternal postpartum depression symptoms are negatively associated with emotion regulation of children born very preterm. *Acta paediatrica* 2019;108(5):869-970. doi: https://doi.org/10.1111/apa.14712
17. Bauer A, Parsonage M, Knapp M, et al. The costs of perinatal mental health problems. London: Centre for Mental Health and London School of Economics, 2014.
18. Berger A, Bachmann N, Signorell A, et al. Perinatal mental disorders in Switzerland: prevalence estimates and use of mental-health services. *Swiss Medical Weekly* 2017;147(0910):w14417. doi: https://doi.org/10.4414/smw.2017.14417
19. Horsch A. Post traumatic stress disorder following childbirth and pregnancy loss. In: Beinard H, Kennedy P, S. L, eds. Clinical Psychology in Practice. Oxford: Blackwell, BPS 2009:274-87.
20. Bradley R, Slade P. A review of mental health problems in fathers following the birth of a child. *Journal of reproductive and infant psychology* 2011;29(1):19-42. doi: https://doi.org/10.1080/02646838.2010.513047
21. Bradley R, Slade P, Leviston A. Low rates of PTSD in men attending childbirth: A preliminary study. *British Journal of Clinical Psychology* 2008;47(3):295-302. doi: 10.1348/014466508x279495
22. Lane A, Keville R, Morris M, et al. Postnatal depression and elation among mothers and their partners: prevalence and predictors. *British Journal of Psychiatry* 1997;171(6):550-55. doi: https://doi.org/10.1192/bjp.171.6.550
23. Horsch A, Jacobs I, Gilbert L, et al. Impact of perinatal asphyxia on parental mental health and bonding with the infant: a questionnaire survey of Swiss parents. *BMJ Paediatrics Open* 2017;1(1):e000059. doi: https://doi.org/10.1136/bmjpo-2017-000059
24. Ayers S, Wright DB, Wells N. Symptoms of post-traumatic stress disorder in couples after birth: association with the couple's relationship and parent–baby bond. *Journal of Reproductive and Infant Psychology* 2007;25(1):40-50. doi: https://doi.org/10.1080/02646830601117175
25. Lamb ME. The role of the father in child development. 4th ed. Hoboken, N.J.: John Wiley & Sons 2004.
26. Paulson JF, Keefe HA, Leiferman JA. Early parental depression and child language development. *Journal of Child Psychology and Psychiatry* 2009;50(3):254-62. doi: https://doi.org/10.1111/j.1469-7610.2008.01973.x
27. Ramchandani P, Psychogiou L. Paternal psychiatric disorders and children's psychosocial development. *The Lancet* 2009;374(9690):646-53. doi: https://doi.org/10.1016/s0140-6736(09)60238-5
28. de Kloet ER, Joels M, Holsboer F. Stress and the brain: from adaptation to disease. *Nature reviews Neuroscience* 2005;6(6):463-75. doi: 10.1038/nrn1683
29. Rohleder N, Nater UM, Wolf JM, et al. Psychosocial stress-induced activation if salivary alpha-amylase. An indicator of sympathetic activity? *Annals of the New York Academy of Sciences* 2004;1032(1):258-63. doi: https://doi.org/10.1196/annals.1314.033

For peer review only - http://bmjopen.bmj.com/site/about/guidelines.xhtml

30. Nater UM, Rohleder N, Scholtz W, et al. Determinants of the diurnal course of salivary alpha-amylase. *Psychoneuroendocrinology* 2007;32(4):392-401. doi: https://doi.org/10.1016/j.psyneuen.2007.02.007
31. Ehlert U, Gaab J, Heinrichs M. Psychoneuroendocrinological contributions to the etiology of depression, posttraumatic stress disorder, and stress-related bodily disorders: the role of the hypothalamus–pituitary–adrenal axis. *Biological psychology* 2001;57(1-3):141-52. doi: https://doi.org/10.1016/s0301-0511(01)00092-8
32. De Kloet ER, Vermetten E, Heijnen CJ, et al. Enhanced cortisol suppression in response to dexamethasone administration in traumatized veterans with and without posttraumatic stress disorder. *Psychoneuroendocrinology* 2007;32(3):215-26. doi: https://doi.org/10.1016/j.psyneuen.2006.12.009
33. Neylan TC, Brunet A, Pole N, et al. PTSD symptoms predict waking salivary cortisol levels in police officers. *Psychoneuroendocrinology* 2005;30(4):373-81. doi: https://doi.org/10.1016/j.psyneuen.2004.10.005
34. Wessa M, Rohleder N, Kirschbaum C, et al. Altered cortisol awakening response in posttraumatic stress disorder. *Psychoneuroendocrinology* 2006;31(2):209-15. doi: https://doi.org/10.1016/j.psyneuen.2005.06.010
35. Yehuda R, Golier JA, Kaufman S. Circadian rhythm of salivary cortisol in Holocaust survivors with and without PTSD. *American Journal of Psychiatry* 2005;162(5):998-1000. doi: https://doi.org/10.1176/appi.ajp.162.5.998
36. Pinto RJ, Correia-Santos P, Costa-Leite J, et al. Cortisol awakening response among women exposed to intimate partner violence. *Psychoneuroendocrinology* 2016;74:57-64. doi: http://dx.doi.org/10.1016/j.psyneuen.2016.08.024
37. Young EA, Breslau N. Cortisol and catecholamines in posttraumatic stress disorder: an epidemiologic community study. *Archives of General Psychiatry* 2004;61(4):394-401. doi: https://doi.org/10.1001/archpsyc.61.4.394
38. Shalev AY, Videlock EJ, Peleg T, et al. Stress hormones and posttraumatic stress disorder in civilian trauma victims: a longitudinal study. Part I: HPA axis responses. *The International Journal of Neuropsychopharmacology* 2008;11(3):365-72. doi: https://doi.org/10.1017/s1461145707008127
39. Meewisse ML, Reitsma JB, De Vries G-J, et al. Cortisol and post-traumatic stress disorder in adults. *British Journal of Psychiatry* 2007;191(5):387-92. doi: https://doi.org/10.1192/bjp.bp.106.024877
40. Habersaat S, Borghini A, Nessi J, et al. Posttraumatic stress symptoms and cortisol regulation in mothers of very preterm infants. *Stress and Health* 2014;30(2):134-41. doi: https://doi.org/10.1002/smi.2503
41. Santa Ana EJ, Saladin ME, Back SE, et al. PTSD and the HPA axis: Differences in response to the cold pressor task among individual with child vs. adult trauma. *Psychoneuroendocrinology* 2006;31(4):501-09. doi: https://doi.org/10.1016/j.psyneuen.2005.11.009
42. Bremner JD, Vythilingam M, Vermetten E, et al. Cortisol response to a cognitive stress challenge in posttraumatic stress disorder (PTSD) related to childhood abuse. *Psychoneuroendocrinology* 2003;28(6):733-50. doi: https://doi.org/10.1016/s0306-4530(02)00067-7
43. Vyas A, Bernal S, Chattarji S. Effects of chronic stress on dendritic arborization in the central and extended amygdala. *Brain Research* 2003;965(1-2):290-94. doi: https://doi.org/10.1016/s0006-8993(02)04162-8

For peer review only - http://bmjopen.bmj.com/site/about/guidelines.xhtml

44. Fries E, Hesse J, Hellhammer J, et al. A new view on hypocortisolism. *Psychoneuroendocrinology* 2005;30(10):1010-16. doi: https://doi.org/10.1016/j.psyneuen.2005.04.006
45. Chrousos GP. Stress and disorders of the stress system. *Nature reviews endocrinology* 2009;5(7):374-81. doi: https://doi.org/10.1038/nrendo.2009.106
46. Beauchaine TP, Thayer JF. Heart rate variability as a transdiagnostic biomarker of psychopathology. *International Journal of Psychophysiology* 2015;98(2):338-50. doi: https://doi.org/10.1016/j.ijpsycho.2015.08.004
47. Nagpal M, Gleichauf K, Ginsberg J. Meta-analysis of heart rate variability as a psychophysiological indicator of posttraumatic stress disorder. *Trauma & Treatment* 2013;3(1) doi: https://doi.org/10.4172/2167-1222.1000182
48. Norte CE, Souza GG, Vilete L, et al. They know their trauma by heart: an assessment of psychophysiological failure to recover in PTSD. *Journal of affective disorders* 2013;150(1):136-41. doi: https://doi.org/10.1016/j.jad.2012.11.039
49. Maher MJ, Rego SA, Asnis GM. Sleep disturbances in patients with post-traumatic stress disorder. *CNS Drugs* 2006;20(7):567-90. doi: https://doi.org/10.2165/00023210-200620070-00003
50. Porcheret K, Holmes EA, Goodwin GM, et al. Psychological impact of an analogue traumatic event reduced by sleep deprivation. *Sleep* 2015;38(7):1017-25. doi: http://dx.doi.org/10.5665/sleep.4802
51. Kleim B, Wysokowsky J, Schmid N, et al. Effects of sleep after experimental trauma on intrusive emotional memories. *Sleep* 2016;39(12):2125-32. doi: https://doi.org/10.5665/sleep.6310
52. Woud ML, Cwik JC, Blackwell SE, et al. Does napping enhance the effects of Cognitive Bias Modification-Appraisal training? An experimental study. *PloS one* 2018;13(2):e0192837. doi: https://doi.org/10.1371/journal.pone.0192837
53. Porcheret K, van Heugten–van der Kloet D, Goodwin GM, et al. Investigation of the impact of total sleep deprivation at home on the number of intrusive memories to an analogue trauma. *Translational psychiatry* 2019;9(1) doi: https://doi.org/10.1038/s41398-019-0403-z
54. Feldman R. Parent-Infant synchrony and the construction of shared timing: psychological precursors, developmental outcomes, and risk conditions *Journal of Child Psychology and Psychiatry* 2007;48(3/4):329-54. doi: https://doi.org/10.1111/j.1469-7610.2006.01701.x
55. Treyvaud K, Anderson VA, Lee KJ, et al. Parental mental health and early social-emotional development of children born very preterm. *Journal of pediatric psychology* 2010;35(7):768-77. doi: https://doi.org/10.1093/jpepsy/jsp109
56. Blandon AY, Calkins SD, Keane SP, et al. Individual differences in trajectories of emotion regulation processes: the effects of maternal depressive symptomatology and children's physiological regulation. *Developmental Psychology* 2008;44(4):1110-23. doi: https://doi.org/10.1037/0012-1649.44.4.1110
57. Eisenberg N, Valiente C, Morris AS, et al. Longitudinal relations among parental emotional expressivity, children's regulation, and quality of socio-emotional functioning *Developmental Psychology* 2003;39(1):3-19. doi: https://doi.org/10.1037/0012-1649.39.1.3
58. Feng X, Shaw DS, Kovacs M, et al. Emotion regulation in preschoolers: the roles of behavioral inhibition, maternal affective behavior, and maternal depression. *Journal*

For peer review only - http://bmjopen.bmj.com/site/about/guidelines.xhtml

of *Child Psychology and Psychiatry* 2008;49(2):132-41. doi: https://doi.org/10.1111/j.1469-7610.2007.01828.x
59. Francis DD, Meaney MJ. Maternal care and the development of stress responses. *Current Opinion in Neurobiology* 1999;9(1):128-34. doi: https://doi.org/10.1016/s0959-4388(99)80016-6
60. Liu D, Diorio J, Tannenbaum B, et al. Maternal care, hippocampal glucocorticoid receptors, and hypothalamic-pituitary-adrenal responses to stress. *Science* 1997;277(5332):1659-62. doi: https://doi.org/10.1126/science.277.5332.1659
61. Gunzenhauser N. Infant stimulation: for whom, what kind, when, and how much? Skillman, NJ: Johnson and Johnson 1987.
62. Schore AN. Affect regulation and the origin of the self. Hillsdale, NJ: Lawrence Erlbaum Associates, Inc 1994.
63. Liu K, Ruggero CJ, Goldstein B, et al. Elevated cortisol in healthy female adolescent offspring of mothers with posttraumatic stress disorder. *Journal of Anxiety Disorders* 2016;40:37-43. doi: http://dx.doi.org/10.1016/j.janxdis.2016.04.003
64. Essex MJ, Klein MH, Cho E, et al. Maternal stress begining in infancy may sensitize children to later stress exposure : Effects on cortisol and behavior. *Biological Psychiatry* 2002;52(8):776 - 84. doi: https://doi.org/10.1016/s0006-3223(02)01553-6
65. Lupien SJ, King S, Meaney MJ, et al. Child's stress hormone levels correlate with mother's socio-economic status and depressive state. *Biological Psychiatry* 2000;48(10):976 - 80. doi: https://doi.org/10.1016/s0006-3223(00)00965-3
66. Warren SL, Gunnar MR, Kagan J, et al. Maternal panic disorder: infant temperament, neurophysiology and parentiong behaviors. *Journal of the American Academy of Child and Adolescent Psychiatry* 2003;42(7):814 - 25 doi: https://doi.org/10.1097/01.chi.0000046872.56865.02
67. Yehuda R, Halligan SL, Bierer LM. Cortisol levels in adult offspring of Holocaust survivors: relation to PTSD symptom severity in the parent and child. *Psychoneuroendocrinology* 2002;27(1-2):171-80. doi: https://doi.org/10.1016/s0306-4530(01)00043-9
68. Ehlert U. Understanding the trans-generational consequences of prenatal stress. *Journal of psychosomatic research* 2013;75(4):297-98. doi: https://doi.org/10.1016/j.jpsychores.2013.09.002
69. Muller-Nix C, Forcada-Guex M, Pierrehumbert B, et al. Prematurity, maternal stress and mother-child interactions. *Early Human Development* 2004;79(2):145-58. doi: https://doi.org/10.1016/j.earlhumdev.2004.05.002
70. Schmücker G, Brisch K-H, Köhntop B, et al. The influence of prematurity, maternal anxiety, and infants' neurobiological risk on mother - infant interactions. *Infant Mental Health Journal* 2005;26(5):423 - 41. doi: https://doi.org/10.1002/imhj.20066
71. Zelkowitz P, Papageorgiou A. Maternal anxiety: an emerging prognostic factor in neonatology. *Acta Paediatrica* 2005;94(12):1704 - 05 doi: https://doi.org/10.1080/08035250500355030
72. Korja R, Savonlahti E, Ahlqvist-Bjorkroth S, et al. Maternal depression is associated with mother-infant interaction in preterm infants. *Acta paediatrica* 2008;97(6):724-30. doi: https://doi.org/10.1111/j.1651-2227.2008.00733.x
73. Crockett EE, Holmes BM, Granger DA, et al. Maternal Disrupted Communication During Face-to-Face Interaction at 4 months: Relation to Maternal and Infant Cortisol Among at-Risk Families. *Infancy* 2013;18(6):1111-34. doi: https://doi.org/10.1111/infa.12015

For peer review only - http://bmjopen.bmj.com/site/about/guidelines.xhtml

74. Ahnert L, Gunnar MR, Lamb ME, et al. Transition to Child Care: Associations with Infant–Mother Attachment, Infant Negative Emotion, and Cortisol Elevations. *Child Development* 2004;75(3):639-50. doi: https://doi.org/10.1111/j.1467-8624.2004.00698.x
75. Domes G, Heinrichs M, Reichwald U, et al. Hypothalamic-pituitary-adrenal axis reactivity to psychological stress and memory in middle-aged women: high responders exhibit enhanced declarative memory performance. *Psychoneuroendocrinology* 2002;27(7):843 - 53. doi: https://doi.org/10.1016/s0306-4530(01)00085-3
76. Nachmias M, Gunnar M, Mangelsdorf S, et al. Behavioral inhibition and stress reactivity: the moderating role of attachment security. *Child development* 1996;67(2):508-22. doi: https://doi.org/10.1111/j.1467-8624.1996.tb01748.x
77. Spangler G, Scheubeck R. Behavioral organization in newborns and its relation to adrenocortical and cardiac activity. *Child Development* 1993;64:622-33.
78. Cordero MI, Moser DA, Manini A, et al. Effects of interpersonal violence-related post-traumatic stress disorder (PTSD) on mother and child diurnal cortisol rhythm and cortisol reactivity to a laboratory stressor involving separation. *Hormones and behavior* 2017;90:15-24. doi: https://doi.org/10.1016/j.yhbeh.2017.02.007
79. Dierckx B, Tulen JH, van den Berg MP, et al. Maternal psychopathology influences infant heart rate variability: Generation R Study. *Psychosomatic Medicine* 2009;71(3):313-21. doi: https://doi.org/10.1097/psy.0b013e318198a82c
80. Braeken MA, Kemp AH, Outhred T, et al. Pregnant mothers with resolved anxiety disorders and their offspring have reduced heart rate variability: implications for the health of children. *PloS one* 2013;8(12):e83186. doi: https://doi.org/10.1371/journal.pone.0083186
81. Jovanovic T, Smith A, Kamkwalala A, et al. Physiological markers of anxiety are increased in children of abused mothers. *Journal of Child Psychology and Psychiatry* 2011;52(8):844-52. doi: https://doi.org/10.1111/j.1469-7610.2011.02410.x
82. Bastos MH, Furuta M, Small R, et al. Debriefing interventions for the prevention of psychological trauma in women following childbirth. *Cochrane Database of Systematic Reviews* 2015 doi: https://doi.org/10.1002/14651858.cd007194.pub2
83. O'Donnell ML, Elliott P, Lau W, et al. PTSD symptom trajectories: From early to chronic response. *Behaviour research and therapy* 2007;45(3):601-06. doi: https://doi.org/10.1016/j.brat.2006.03.015
84. Brewin CR. Episodic memory, perceptual memory, and their interaction: foundations for a theory of posttraumatic stress disorder. *Psychol Bulletin* 2014;140(1):69-97. doi: https://doi.org/10.1037/a0033722
85. Holmes EA, James EL, Coode-Bate T, et al. Can playing the computer game "Tetris" reduce the build-up of flashbacks for trauma? A proposal from cognitive science. *PLoS ONE v* 2009;4(1):e4153. doi: https://doi.org/10.1371/journal.pone.0004153
86. Holmes EA, James EL, Kilford EJ, et al. Key steps in developing a cognitive vaccine against traumatic flashbacks: visuospatial Tetris versus verbal Pub Quiz. *PloS one* 2010;5(11):e13706. doi: https://doi.org/10.1371/journal.pone.0013706
87. Kindt M, van den Hout M, Arntz A, et al. The influence of data-driven versus conceptually-driven processing on the development of PTSD-like symptoms. *Journal of behavior therapy and experimental psychiatry* 2008;39(4):546-57. doi: https://doi.org/10.1016/j.jbtep.2007.12.003

For peer review only - http://bmjopen.bmj.com/site/about/guidelines.xhtml

88. Deeprose C, Zhang S, Dejong H, et al. Imagery in the aftermath of viewing a traumatic film: Using cognitive tasks to modulate the development of involuntary memory. *Journal of behavior therapy and experimental psychiatry* 2012;43(2):758-64. doi: https://doi.org/10.1016/j.jbtep.2011.10.008
89. Walker MP, Brakefield T, Hobson JA, et al. Dissociable stages of human memory consolidation and reconsolidation. *Nature* 2003;425(6958):616-20. doi: https://doi.org/10.1038/nature01930
90. McGaugh JL. Memory--a Century of Consolidation. *Science* 2000;287(5451):248-51. doi: 10.1126/science.287.5451.248
91. Nader K, Schafe GE, LeDoux JE. The labile nature of consolidation theory. *Nature Reviews Neuroscience* 2000;1(3):216-19. doi: https://doi.org/10.1038/35044580
92. Iyadurai L, Visser RM, Lau-Zhu A, et al. Intrusive memories of trauma: A target for research bridging cognitive science and its clinical application. *Clinical psychology review* 2018;69:67-82. doi: https://doi.org/10.1016/j.cpr.2018.08.005
93. Iyadurai L, Blackwell SE, Meiser-Stedman R, et al. Preventing intrusive memories after trauma via a brief intervention involving Tetris computer game play in the emergency department: a proof-of-concept randomized controlled trial. *Molecular psychiatry* 2018;23(3):674-82. doi: https://doi.org/10.1038/mp.2017.23
94. Foa EB, Cashman L, Jaycox L, et al. The validation of a self-report measure of posttraumatic stress disorder: the Posttraumatic Diagnostic Scale. *Psychological assessment* 1997;9(4):445-51. doi: https://doi.org/10.1037/1040-3590.9.4.445
95. Kim J, Shin W. How to Do Random Allocation (Randomization). *Clinics in Orthopedic Surgery* 2014;6(1):103-09. doi: https://doi.org/10.4055/cios.2014.6.1.103
96. Furuta M, Horsch A, Ng ES, et al. Effectiveness of trauma-focused psychological therapies for treating post-traumatic stress disorder symptoms in women following childbirth: a systematic review and meta-analysis. *Frontiers in psychiatry* 2018;9 doi: https://doi.org/10.3389/fpsyt.2018.00591
97. Roberts NP, Kitchiner NJ, Kenardy J, et al. Multiple session early psychological interventions for the prevention of post-traumatic stress disorder. *Cochrane Database of Systematic Reviews* 2009 doi: https://doi.org/10.1002/14651858.cd006869.pub2
98. Sijbrandij M, Kleiboer A, Bisson JI, et al. Pharmacological prevention of post-traumatic stress disorder and acute stress disorder: a systematic review and meta-analysis. *The Lancet Psychiatry* 2015;2(5):413-21. doi: https://doi.org/10.1016/s2215-0366(14)00121-7
99. Rose SC, Bisson J, Churchill R, et al. Psychological debriefing for preventing post traumatic stress disorder (PTSD). *Cochrane Database of Systematic Reviews* 2002 doi: https://doi.org/10.1002/14651858.cd000560
100. Weathers FW, Bovin MJ, Lee DJ, et al. The Clinician-Administered PTSD Scale for DSM–5 (CAPS-5): Development and Initial Psychometric Evaluation in Military Veterans. *Psychological Assessment* 2018;30(3):383-95. doi: https://doi.org/10.1037/pas0000486
101. Blevins CA, Weathers FW, Davis MT, et al. The posttraumatic stress disorder checklist for DSM-5 (PCL-5): Development and initial psychometric evaluation. *Journal of Traumatic Stress* 2015;28(6):489-98. doi: https://doi.org/10.1002/jts.22059
102. Wild D, Grove A, Martin M, et al. Principles of Good Practice for the Translation and Cultural Adaptation Process for Patient-Reported Outcomes (PRO) Measures: report

For peer review only - http://bmjopen.bmj.com/site/about/guidelines.xhtml

of the ISPOR Task Force for Translation and Cultural Adaptation. *Value Health* 2005;8(2):94-104. doi: https://doi.org/10.1111/j.1524-4733.2005.04054.x
103. Bovin MJ, Marx BP, Weathers FW, et al. Psychometric properties of the PTSD checklist for diagnostic and statistical manual of mental disorders–fifth edition (PCL-5) in veterans. *Psychological Assessment* 2016;28(11):1379-91. doi: https://doi.org/10.1037/pas0000254
104. Ashbaugh AR, Houle-Johnson S, Herbert C, et al. Psychometric Validation of the English and French Versions of the Posttraumatic Stress Disorder Checklist for DSM-5 (PCL-5). *PloS one* 2016;11(10):e0161645. doi: https://doi.org/10.1371/journal.pone.0161645
105. Bryant RA, Moulds ML, Guthrie RM. Acute Stress Disorder Scale: a self-report measure of acute stress disorder. *Psychological Assessment* 2000;12(1):61-68. doi: https://doi.org/10.1037//1040-3590.12.1.61
106. American Psychiatric Association. Diagnostic and statistical manual of mental disorders. 4th ed., text rev. ed. Washington, DC: American Psychiatric Publishing 2000.
107. Zigmond AS, Snaith RP. The hospital anxiety and depression scale. *Acta psychiatrica scandinavica* 1983;67(6):361-70. doi: https://doi.org/10.1111/j.1600-0447.1983.tb09716.x
108. Bocerean C, Dupret E. A validation study of the Hospital Anxiety and Depression Scale (HADS) in a large sample of French employees. *BMC Psychiatry* 2014;14(1) doi: https://doi.org/10.1186/s12888-014-0354-0 [published Online First: 2014/12/17]
109. Cox JL, Holden JM, Sagovsky R. Detection of postnatal depression. Development of the 10-item Edinburgh Postnatal Depression Scale. *British Journal of Psychiatry* 1987;150(6):782-86. doi: https://doi.org/10.1192/bjp.150.6.782
110. Guedeney N, Fermanian J. Validation study of the French version of the Edinburgh Postnatal Depression Scale (EPDS): new results about use and psychometric properties. *Eur Psychiatry* 1998;13(2):83-89. doi: https://doi.org/10.1016/s0924-9338(98)80023-0
111. Esliger DW, Rowlands AV, Hurst TL, et al. Validation of the GENEA Accelerometer. *Medicine & Science in Sports & Exercise* 2011;43(6):1085-93. doi: 10.1249/MSS.0b013e31820513be
112. Buysse DJ, Reynolds CF, Monk TH, et al. The Pittsburgh sleep quality index: A new instrument for psychiatric practice and research. *Psychiatry research* 1989;28(2):193-213. doi: https://doi.org/10.1016/0165-1781(89)90047-4
113. Blais F, Gendron L, Mimeault V, et al. Evaluation de l'insomnie: Validation de trois questionnaires. *L'Encéphale: Revue de psychiatrie clinique biologique et thérapeutique* 1997;23(6):447-53.
114. Ait-Aoudia M, Levy PP, Bui E, et al. Validation of the French version of the Pittsburgh Sleep Quality Index Addendum for posttraumatic stress disorder. *European journal of psychotraumatology* 2013;4(1):19298. doi: https://doi.org/10.3402/ejpt.v4i0.19298
115. Kirschbaum C, Pirke K-M, Hellhammer DH. The 'Trier Social Stress Test'–a tool for investigating psychobiological stress responses in a laboratory setting. *Neuropsychobiology* 1993;28(1-2):76-81. doi: https://doi.org/10.1159/000119004
116. Brazelton TB, Nugent JK. The Neontal Behavioral Assessment Scale. 4th ed. London: Mac Keith Press 2011.
117. Tronick E, Als H, Adamson L, et al. The infant's response to entrapment between contradictory messages in face-to-face interaction. *Journal of the American Academy*

For peer review only - http://bmjopen.bmj.com/site/about/guidelines.xhtml

of Child Psychiatry 1978;17(1):1-13. doi: https://doi.org/10.1016/s0002-7138(09)62273-1
118. Provenzi L, Giusti L, Montirosso R. Do infants exhibit significant cortisol reactivity to the Face-to-Face Still-Face paradigm? A narrative review and meta-analysis. *Developmental Review* 2016;42:34-55. doi: https://doi.org/10.1016/j.dr.2016.07.001
119. Haley DW, Stansbury K. Infant stress and parent responsiveness: regulation of physiology and behavior during still-face and reunion. *Child development* 2003;74(5):1534-46. doi: https://doi.org/10.1111/1467-8624.00621
120. DiCorcia JA, Snidman N, Sravish AV, et al. Evaluating the Nature of the Still-Face Effect in the Double Face-to-Face Still-Face Paradigm Using Different Comparison Groups. *Infancy* 2016;21(3):332-52. doi: https://doi.org/10.1111/infa.12123
121. Taylor A, Atkins R, Kumar R, et al. A new Mother-to-Infant Bonding Scale: links with early maternal mood. *Archives of women's mental health* 2005;8(1):45-51. doi: https://doi.org/10.1007/s00737-005-0074-z
122. Bienfait M, Haquet A, Maury M, et al. Traduction française de l'autoquestionnaire MIBS (Mother to Infant Bonding Scale) et validation comme évaluation du lien mère-nouveau-né en maternité. *Devenir* 2017;29(4):233-53. doi: https://doi.org/10.3917/dev.174.0233
123. Biringen Z. The Emotional Availability (EA) Scales and EA Zones Evaluation: Infancy/early childhood version; middle childhood/youth versions; therapist/interventionist/professional manual; couple relationship manual. 4 ed. Colorado: Boulder 2008.
124. Biringen Z, Derscheid D, Vliegen N, et al. Emotional Availability (EA): Theoretical background, empirical research using the EA Scales and clinical implications. . *Developmental Review* 2014;34:114-67. doi: https://doi.org/10.1016/j.dr.2014.01.002
125. Putnam SP, Helbig AL, Gartstein MA, et al. Development and Assessment of Short and Very Short Forms of the Infant Behavior Questionnaire–Revised. *Journal of Personality Assessment* 2014;96(4):445-58. doi: https://doi.org/10.1080/00223891.2013.841171
126. Bayley N, Reuner G. Bayley scales of infant and toddler development: Bayley-III: Harcourt Assessment, Psych. Corporation San Antonio, Tex, USA 2006.
127. Albers EM, Marianne Riksen-Walraven J, Sweep FC, et al. Maternal behavior predicts infant cortisol recovery from a mild everyday stressor. *Journal of Child Psychology and Psychiatry* 2008;49(1):97-103. doi: https://doi.org/10.1111/j.1469-7610.2007.01818.x
128. Largo RH, Pfister D, Molinari L, et al. Significance of prenatal, perinatal and postnatal factors in the development of AGA preterm infants at five to seven years. *Developmental Medicine & Child Neurology* 1989;31(4):440-56. doi: https://doi.org/10.1111/j.1469-8749.1989.tb04022.x
129. Parry G, Tucker J, Tarnow-Mordi W, et al. CRIB II: an update of the clinical risk index for babies score. *Lancet* 2003;361(9371):1789-91. doi: https://doi.org/10.1016/s0140-6736(03)13397-1
130. Harris PA, Taylor R, Minor BL, et al. The REDCap consortium: Building an international community of software platform partners. *Journal of Biomedical Informatics* 2019;95:103208. doi: https://doi.org/10.1016/j.jbi.2019.103208

For peer review only

For peer review only - http://bmjopen.bmj.com/site/about/guidelines.xhtml

131. Harris PA, Taylor R, Thielke R, et al. Research electronic data capture (REDCap)—a metadata-driven methodology and workflow process for providing translational research informatics support. *Journal of Biomedical Informatics* 2009;42(2):377-81. doi: https://doi.org/10.1016/j.jbi.2008.08.010
132. Camm AJ, Malik M, Bigger J, et al. Heart rate variability: standards of measurement, physiological interpretation and clinical use. . *European Heart Journal* 1996;17(3):354-81. doi: https://doi.org/10.1093/oxfordjournals.eurheartj.a014868
133. Tarvainen MP, Niskanen JP, Lipponen JA, et al. Kubios HRV--heart rate variability analysis software. *Computer methods and programs in biomedicine* 2014;113(1):210-20. doi: https://doi.org/10.1016/j.cmpb.2013.07.024 [published Online First: 2013/09/24]
134. Razurel C, Kaiser B, Dupuis M, et al. Validation of the post-delivery perceived stress inventory. *Psychology, Health & Medicine* 2014;19(1):70-82. doi: https://doi.org/10.1080/13548506.2013.774431
135. Koch FS, Sepa A, Ludvigsson J. Psychological stress and obesity. *The Journal of Pediatrics* 2008;153(6):839-44. doi: https://doi.org/10.1016/j.jpeds.2008.06.016
136. Obel C, Hedegaard M, Henriksen TB, et al. Stress and salivary cortisol during pregnancy. *Psychoneuroendocrinology* 2005;30(7):647-56.
137. Razurel C, Kaiser B, Dupuis M, et al. Validation of the Postnatal Perceived Stress Inventory in a French Speaking Population of Primiparous Women. *Journal of Obstetric, Gynecologic, & Neonatal Nursing* 2013;42(6):685-96. doi: https://doi.org/10.1111/1552-6909.12251
138. Abidin RR. Parenting stress index-short form: Pediatric Psychology Press Charlottesville, VA 1990.
139. Haskett ME, Ahern LS, Ward CS, et al. Factor structure and validity of the parenting stress index-short form. *Journal of Clinical Child & Adolescent Psychology* 2006;35(2):302-12. doi: https://doi.org/10.1207/s15374424jccp3502_14
140. Moser A, Stuck AE, Silliman RA, et al. The eight-item modified Medical Outcomes Study Social Support Survey: psychometric evaluation showed excellent performance. *Journal of clinical epidemiology* 2012;65(10):1107-16. doi: https://doi.org/10.1016/j.jclinepi.2012.04.007
141. Busby DM, Christensen C, Crane DR, et al. A revision of the Dyadic Adjustment Scale for use with distressed and nondistressed couples: Construct hierarchy and multidimensional scales. *Journal of Marital and family Therapy* 1995;21(3):289-308. doi: https://doi.org/10.1111/j.1752-0606.1995.tb00163.x
142. Crane DR, Middleton KC, Bean RA. Establishing criterion scores for the Kansas marital satisfaction scale and the revised dyadic adjustment scale. *American Journal of Family Therapy* 2000;28(1):53-60. doi: https://doi.org/10.1080/019261800261815
143. Horne JA, Ostberg O. A self-assessment questionnaire to determine morningness-eveningness in human circadian rhythms. *International Journal of Chronobiology* 1976;4:97-110.
144. Esliger DW, Rowlands AV, Hurst TL, et al. Validation of the GENEA Accelerometer. 2011
145. Blake DD, Weathers FW, Nagy LM, et al. The development of a clinician-administered PTSD scale. *Journal of Traumatic Stress* 1995;8(1):75-90. doi: https://doi.org/10.1007/bf02105408

For peer review only - http://bmjopen.bmj.com/site/about/guidelines.xhtml

146. Dubowitz L, Mercuri E, Dubowitz V. An optimality score for the neurologic examination of the term newborn. *The Journal of pediatrics* 1998;133(3):406-16. doi: https://doi.org/10.1016/s0022-3476(98)70279-3

For peer review only

For peer review only - http://bmjopen.bmj.com/site/about/guidelines.xhtml

Figure legends

Figure 1. Study variables: Processes in mothers, partner, infants, and their interactions following traumatic childbirth

Figure 2. Flowchart of study procedures

For peer review only

For peer review only - http://bmjopen.bmj.com/site/about/guidelines.xhtml

Table 1. Overview of primary, secondary and other outcomes, measures, and time points

	DOMAIN	VARIABLES	INSTRUMENTS	TIMING<br>6h after ECS (T1)	≤ 1 week (T2)	6 weeks (T3)	6 months (T4)
**MOTHER**	Sociodemographic and medical data	Sociodemographic variables	Demographic questionnaire		X	X	X
		Medical data	Obstetric data and pregnancy outcomes from medical records		X		X
			Menstrual cycle (if applicable)				X
			Breastfeeding diary/questions		X	X	X
	Acceptability and expectancy of the intervention	Satisfaction and expectancy	Feedback questionnaire	X			
	Maternal psychological vulnerability	Intrusive trauma-related memories	Trauma-related intrusive memories diary [4]		X		
		Stress exposure and perception	Post-delivery Perceived Stress Inventory (PDPSI) [134]		X		
			Major life events [135 136]		X		X
			Postnatal Perceived Stress Inventory (PPSI) [137]				X
			Parenting Stress Index – Short Form (PSI-SF) [138 139]				X
		PTSD	Clinician-administered PTSD scale (CAPS) [102]			X	X
			PTSD Checklist (PCL-5) [101]			X	X
		ASD	Acute Stress Disorder Scale (ASDS) [105]	X	X		

For peer review only

	Anxiety	Hospital Anxiety and Depression Scale: anxiety subscale (HADS) [107]	X	X	X	X
	Depression	Edinburgh Postnatal Depression Scale (EPDS) [109] [109]	X	X	X	X
	Social support	Modified Medical Outcomes Study Social Support Survey (MOS-8) [140]		X	X	X
		Revised Dyadic Adjustment Scale (RDAS) [141] [142]		X	X	X
	Sleep	Pittsburgh Sleep Quality Index Addendum for posttraumatic stress disorder (PSQI-A) [114]			X	X
		Morningness-Eveningness questionnaire (MEQ) [143]		X		
		Pittsburgh Sleep Quality Index (PSQI) [112]		X	X	X
		Sleep diary		X		
		Overnight accelerometer assessments (GENEActiv®)[144]		X		X
Maternal physiological stress responses	Physiological regulation	Baseline cortisol and cortisol daily profile (saliva)		X		X
		Resting heart rate and heart rate variability (Firstbeat Bodyguard 2)				X
	Physiological stress reactivity	Cortisol (saliva)				X
		Heart rate variability (Firstbeat Bodyguard 2)				X

For peer review only

**PARTNER**	Sociodemographic data	Sociodemographic variables	Demographic questionnaire		**X**	**X**	**X**
	Paternal psychological vulnerability	Intrusive traumatic memories	Traumatic intrusions diary [4]		**X**		
		Stress exposure and perception	Major life events [135 136]		**X**		**X**
			Parenting Stress Index – Short Form (PSI-SF) [138 139]				**X**
		PTSD	Clinician-administered PTSD scale (CAPS) [102 145]			**X**	**X**
			PTSD Checklist (PCL-5) [101]			**X**	**X**
		ASD	Acute Stress Disorder Scale (ASDS) [105]		**X**		
		Anxiety	Hospital Anxiety and Depression Scale: anxiety subscale (HADS) [107]		**X**	**X**	**X**
		Depression	Edinburgh Postnatal Depression Scale (EPDS) [109]		**X**	**X**	**X**
		Social support	Modified Medical Outcomes Study Social Support Survey (MOS-8) [140]		**X**	**X**	**X**
			Revised Dyadic Adjustment Scale (RDAS) [142 141]		**X**	**X**	**X**
		Perception of ECS-related trauma	Screening questions of the Posttraumatic Diagnostic Scale [94]		**X**		
		Sleep	Pittsburgh Sleep Quality Index (PSQI) [112]		**X**	**X**	**X**
			Pittsburgh Sleep Quality Index Addendum for posttraumatic stress disorder (PSQI-A)[114]			**X**	**X**

For peer review only - http://bmjopen.bmj.com/site/about/guidelines.xhtml

			Morningness-Eveningness questionnaire (MEQ) [143]		**X**		
	Partner physiological responses	Physiological regulation	Cortisol daily profile (saliva)		**X**		**X**
**INFANT**	Sociodemographic and medical data	Sociodemographic variables	Demographic questionnaire (completed by mother)		**X**	**X**	**X**
		Medical data	Neonatal outcomes from medical records		**X**		
	Infant neurodevelopmental vulnerability	Infant irritability	Dubowitz neurologic examination [146]		**X**		
	Infant physiological stress responses	Physiological regulation	Baseline cortisol and cortisol daily profile (saliva)		**X**		**X**
			Resting heart rate and heart rate variability by (Firstbeat Bodyguard 2)		**X**		**X**
		Physiological stress reactivity	Cortisol (saliva)		**X**		**X**
			Heart rate variability (Firstbeat Bodyguard 2)		**X**		**X**
	Developmental outcomes	Neonatal behaviour	Neonatal Behavioural Assessment Scale (NBAS) [116]		**X**		
		Infant development	Infant Behaviour Questionnaire-revised (IBQ-R) [125]				**X***
			Bayley Scales of Infant Development (Bayley-III; clinician-rated) [126]				**X**

For peer review only - http://bmjopen.bmj.com/site/about/guidelines.xhtml

**PARENT-INFANT INTERACTION**	Mother-infant interaction	Maternal sensitivity	Emotional Availability Scale (EAS, clinician-rated) [123 124]				**X**
		Bonding	Mother-to-Infant-Bonding Scale (MIBS) [121]		**X**	**X**	**X**
	Partner-infant interaction	Bonding	Mother-Infant-Bonding Scale (MIBS) [121]		**X**	**X**	**X**

Note: * = completed by mothers and partners

For peer review only

Table 2. Other Outcomes and a brief description of the measures used for each outcome. Participant groups for whom the outcome is relevant are highlighted in bold.

Domain	Instruments	Description
**Maternal and paternal psychological vulnerability**	Morningness-Eveningness Questionnaire (MEQ) [143]	19 multiple-choice questions self-assessment questionnaire investigating the **mother and partner**'s circadian rhythm. Scores are summed to create a total and a higher score indicates a more 'morning' person.
	Modified Medical Outcomes Study Social Support Survey (MOS-8) [140 122]	8-item self-report questionnaire assessing **mother and partner** social support across four functional support scales: emotional/ informative, tangible, affectionate, and positive social interaction. Items are answered on a 6 point scale (0= *Never*, 5 = *Always*).
	Revised Dyadic Adjustment Scale (RDAS) [141 142]	14-item self-report questionnaire measuring the **mother and the partner** relationship satisfaction. Three categories are evaluated: consensus, satisfaction and cohesion.
	Parenting Stress Index - Short Form (PSI-SF)[139 138]	Self-report questionnaire measuring the **mother and partner** parenting stress. It consists of three subscales: parental distress, parent-child dysfunctional interactions, and child difficulties. 36 items are answered on a 1-point Likert scale *(1 = Strongly agree, 5 = Strongly disagree).*
	The Post-delivery Perceived Stress Inventory (PDPSI) [134]	16-item self-report questionnaire assessing **mother's** perceived stress linked to delivery. Each item is a potential stressor the mother may have

		experienced during or after delivery. Mothers are asked whether they found the items more or less stressful using a 5-point Likert scale *(1=Never, 5=Very often).*
	Postnatal Perceived Stress Inventory (PPSI)[137]	19-item self-report questionnaire assessing **maternal** postpartum perceived stress. Each item relates to a potential stressor they may encounter within the postnatal period. Mothers are asked to indicate whether they were more or less stressed on a 5 point scale (1 = Not at all, 5 = Extremely).
	Life Events Questionnaire [135 136]	15-item self-report scale where **mother and partners** are asked to indicate if they have experienced different major life events and how stressful they found these events. One item allows participants to identify an event not mentioned and one asks if a major life event occurred within the first trimester of pregnancy.
	Trauma-related intrusive memories diary [4]	Completed by **partners**; see Measures section for more details.
	Clinician-administered PTSD scale (CAPS) [100]	Completed by **partners**; see Measures section for more details.
	PTSD Checklist (PCL-5) [101]	Completed by **partners**; see Measures section for more details.
	Acute Stress Disorder Scale (ASDS) [105]	Completed by **partners**; see Measures section for more details.
	Edinburgh Postnatal Depression Scale (EPDS) [109]	Completed by **partners**; see Measures section for more details.

For peer review only - http://bmjopen.bmj.com/site/about/guidelines.xhtml

	Anxiety subscale of Hospital Anxiety and Depression Scale (HADS) [107]	Completed by **partners**; see Measures section for more details.
	Pittsburgh Sleep Quality Index (PSQI) [112]	Completed by **partners; s**ee Measures section for more details.
	Pittsburgh Sleep Quality Index Addendum for posttraumatic stress disorder (PSQI-A) [114]	Completed by **partners**; see Measures section for more details.
	Perception of ECS-related trauma	**Partners** complete the 4 screening questions of the Posttraumatic Diagnostic Scale [94]
**Partner physiological regulation**	Cortisol daily profile	Completed by **partners**; see Measures section for more details.
**Partner-infant interaction**	Mother-to-Infant-Bonding Scale (MIBS) [121]	Adapted for partners, **s**ee Measures section for more details
**Demographic Information**	Partner demographics	Adapted for partners, **s**ee Measures section for more details
	Infant demographics	**Mothers** report on their child's weight and height.
**Medical data**	Breastfeeding diary and questions	**Mothers** report on breastfeeding in a 2-day daily diary including breastfeeding length and type (exclusive or mixed).
**Acceptability and expectancy of the intervention**	Self-report questionnaire of satisfaction and acceptability of the intervention	On completing the intervention activity, **mothers** complete 7 items to assess intervention fidelity, satisfaction and acceptability of the intervention.
**Infant neurodevelopmental vulnerability**	Dubowitz neurologic examination [146]	Examination of the **infant** on 34 items subdivided into 6 categories (tone, tone patterns, reflexes, movements, abnormal signs, and behavior). Full examination by a pediatrician follows

For peer review only - http://bmjopen.bmj.com/site/about/guidelines.xhtml

		standardised instructions and takes 10-15 minutes.

For peer review only

For peer review only - http://bmjopen.bmj.com/site/about/guidelines.xhtml

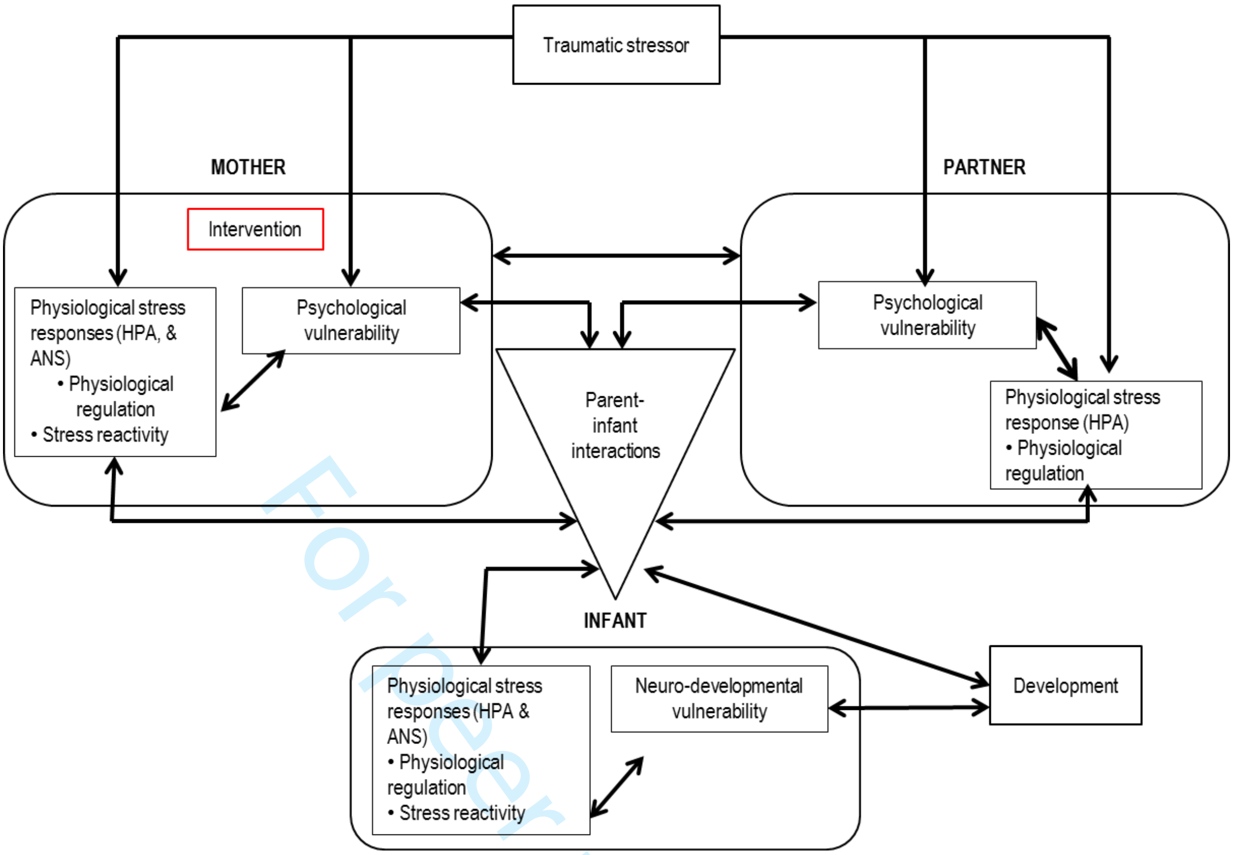

For peer review only - http://bmjopen.bmj.com/site/about/guidelines.xhtml

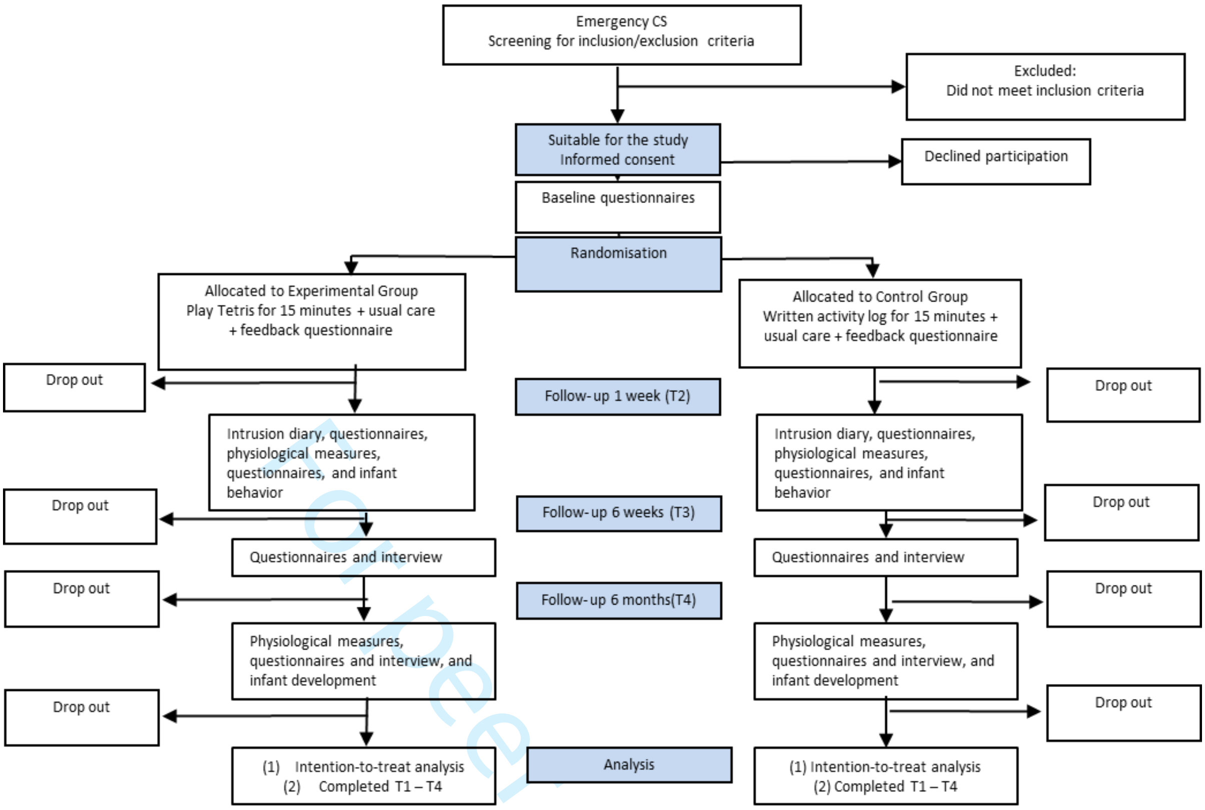

For peer review only

STANDARD PROTOCOL ITEMS: RECOMMENDATIONS FOR INTERVENTIONAL TRIALS

SPIRIT 2013 Checklist: Recommended items to address in a clinical trial protocol and related documents*

Section/item	Item No	Description	Addressed on page number
**Administrative information**			
Title	1	Descriptive title identifying the study design, population, interventions, and, if applicable, trial acronym	1
Trial registration	2a	Trial identifier and registry name. If not yet registered, name of intended registry	6
	2b	All items from the World Health Organization Trial Registration Data Set	n/a
Protocol version	3	Date and version identifier	23
Funding	4	Sources and types of financial, material, and other support	28
Roles and responsibilities	5a	Names, affiliations, and roles of protocol contributors	27
	5b	Name and contact information for the trial sponsor	1
	5c	Role of study sponsor and funders, if any, in study design; collection, management, analysis, and interpretation of data; writing of the report; and the decision to submit the report for publication, including whether they will have ultimate authority over any of these activities	27
	5d	Composition, roles, and responsibilities of the coordinating centre, steering committee, endpoint adjudication committee, data management team, and other individuals or groups overseeing the trial, if applicable (see Item 21a for data monitoring committee)	24
**Introduction**			

For peer review only

Background and rationale	6a	Description of research question and justification for undertaking the trial, including summary of relevant studies (published and unpublished) examining benefits and harms for each intervention	8-14
	6b	Explanation for choice of comparators	16-17
Objectives	7	Specific objectives or hypotheses	14
Trial design	8	Description of trial design including type of trial (eg, parallel group, crossover, factorial, single group), allocation ratio, and framework (eg, superiority, equivalence, noninferiority, exploratory)	14

Methods: Participants, interventions, and outcomes

Study setting	9	Description of study settings (eg, community clinic, academic hospital) and list of countries where data will be collected. Reference to where list of study sites can be obtained	14-15
Eligibility criteria	10	Inclusion and exclusion criteria for participants. If applicable, eligibility criteria for study centres and individuals who will perform the interventions (eg, surgeons, psychotherapists)	14-15
Interventions	11a	Interventions for each group with sufficient detail to allow replication, including how and when they will be administered	16-17
	11b	Criteria for discontinuing or modifying allocated interventions for a given trial participant (eg, drug dose change in response to harms, participant request, or improving/worsening disease)	n/a
	11c	Strategies to improve adherence to intervention protocols, and any procedures for monitoring adherence (eg, drug tablet return, laboratory tests)	14
	11d	Relevant concomitant care and interventions that are permitted or prohibited during the trial	15
Outcomes	12	Primary, secondary, and other outcomes, including the specific measurement variable (eg, systolic blood pressure), analysis metric (eg, change from baseline, final value, time to event), method of aggregation (eg, median, proportion), and time point for each outcome. Explanation of the clinical relevance of chosen efficacy and harm outcomes is strongly recommended	17-22
Participant timeline	13	Time schedule of enrolment, interventions (including any run-ins and washouts), assessments, and visits for participants. A schematic diagram is highly recommended (see Figure)	Table 1<br>Figure 2

For peer review only - http://bmjopen.bmj.com/site/about/guidelines.xhtml

Sample size	14	Estimated number of participants needed to achieve study objectives and how it was determined, including clinical and statistical assumptions supporting any sample size calculations	23
Recruitment	15	Strategies for achieving adequate participant enrolment to reach target sample size	14-15

Methods: Assignment of interventions (for controlled trials)

Allocation:

Sequence generation	16a	Method of generating the allocation sequence (eg, computer-generated random numbers), and list of any factors for stratification. To reduce predictability of a random sequence, details of any planned restriction (eg, blocking) should be provided in a separate document that is unavailable to those who enrol participants or assign interventions	15
Allocation concealment mechanism	16b	Mechanism of implementing the allocation sequence (eg, central telephone; sequentially numbered, opaque, sealed envelopes), describing any steps to conceal the sequence until interventions are assigned	15
Implementation	16c	Who will generate the allocation sequence, who will enrol participants, and who will assign participants to interventions	15-16
Blinding (masking)	17a	Who will be blinded after assignment to interventions (eg, trial participants, care providers, outcome assessors, data analysts), and how	16
	17b	If blinded, circumstances under which unblinding is permissible, and procedure for revealing a participant's allocated intervention during the trial	n/a

Methods: Data collection, management, and analysis

Data collection methods	18a	Plans for assessment and collection of outcome, baseline, and other trial data, including any related processes to promote data quality (eg, duplicate measurements, training of assessors) and a description of study instruments (eg, questionnaires, laboratory tests) along with their reliability and validity, if known. Reference to where data collection forms can be found, if not in the protocol	18-22
	18b	Plans to promote participant retention and complete follow-up, including list of any outcome data to be collected for participants who discontinue or deviate from intervention protocols	n/a

Data management	19	Plans for data entry, coding, security, and storage, including any related processes to promote data quality (eg, double data entry; range checks for data values). Reference to where details of data management procedures can be found, if not in the protocol	22-23
Statistical methods	20a	Statistical methods for analysing primary and secondary outcomes. Reference to where other details of the statistical analysis plan can be found, if not in the protocol	24-25
	20b	Methods for any additional analyses (eg, subgroup and adjusted analyses)	23-24
	20c	Definition of analysis population relating to protocol non-adherence (eg, as randomised analysis), and any statistical methods to handle missing data (eg, multiple imputation)	23-24
**Methods: Monitoring**			
Data monitoring	21a	Composition of data monitoring committee (DMC); summary of its role and reporting structure; statement of whether it is independent from the sponsor and competing interests; and reference to where further details about its charter can be found, if not in the protocol. Alternatively, an explanation of why a DMC is not needed	n/a
	21b	Description of any interim analyses and stopping guidelines, including who will have access to these interim results and make the final decision to terminate the trial	n/a
Harms	22	Plans for collecting, assessing, reporting, and managing solicited and spontaneously reported adverse events and other unintended effects of trial interventions or trial conduct	n/a
Auditing	23	Frequency and procedures for auditing trial conduct, if any, and whether the process will be independent from investigators and the sponsor	n/a
**Ethics and dissemination**			
Research ethics approval	24	Plans for seeking research ethics committee/institutional review board (REC/IRB) approval	24
Protocol amendments	25	Plans for communicating important protocol modifications (eg, changes to eligibility criteria, outcomes, analyses) to relevant parties (eg, investigators, REC/IRBs, trial participants, trial registries, journals, regulators)	23-24

For peer review only - http://bmjopen.bmj.com/site/about/guidelines.xhtml

Consent or assent	26a	Who will obtain informed consent or assent from potential trial participants or authorised surrogates, and how (see Item 32)	24
	26b	Additional consent provisions for collection and use of participant data and biological specimens in ancillary studies, if applicable	24
Confidentiality	27	How personal information about potential and enrolled participants will be collected, shared, and maintained in order to protect confidentiality before, during, and after the trial	24
Declaration of interests	28	Financial and other competing interests for principal investigators for the overall trial and each study site	28
Access to data	29	Statement of who will have access to the final trial dataset, and disclosure of contractual agreements that limit such access for investigators	23
Ancillary and post-trial care	30	Provisions, if any, for ancillary and post-trial care, and for compensation to those who suffer harm from trial participation	n/a
Dissemination policy	31a	Plans for investigators and sponsor to communicate trial results to participants, healthcare professionals, the public, and other relevant groups (eg, via publication, reporting in results databases, or other data sharing arrangements), including any publication restrictions	24
	31b	Authorship eligibility guidelines and any intended use of professional writers	n/a
	31c	Plans, if any, for granting public access to the full protocol, participant-level dataset, and statistical code	n/a
**Appendices**			
Informed consent materials	32	Model consent form and other related documentation given to participants and authorised surrogates	upon request
Biological specimens	33	Plans for collection, laboratory evaluation, and storage of biological specimens for genetic or molecular analysis in the current trial and for future use in ancillary studies, if applicable	n/a

*It is strongly recommended that this checklist be read in conjunction with the SPIRIT 2013 Explanation & Elaboration for important clarification on the items. Amendments to the protocol should be tracked and dated. The SPIRIT checklist is copyrighted by the SPIRIT Group under the Creative Commons "Attribution-NonCommercial-NoDerivs 3.0 Unported" license.

BMJ Open

Improving mental health and physiological stress responses in mothers following traumatic childbirth and in their infants: Study protocol for the Swiss TrAumatic biRth Trial (START)

Journal:	*BMJ Open*
Manuscript ID	bmjopen-2019-032469.R2
Article Type:	Protocol
Date Submitted by the Author:	20-Nov-2019
Complete List of Authors:	Sandoz, Vania; University of Lausanne Faculty of Biology and Medicine, Institute of Higher Education and Research in Healthcare<br>Deforges, Camille; University of Lausanne Faculty of Biology and Medicine, Institute of Higher Education and Research in Healthcare<br>Stuijfzand, Suzannah; University of Lausanne Faculty of Biology and Medicine, Institute of Higher Education and Research in Healthcare<br>Epiney, Manuella; Geneva University Hospitals, Department Woman-Child-Adolescent<br>Vial, Yvan; University Hospital of Lausanne, 3Obstetrics and Gynecology Service, Woman-Mother-Child Department<br>Sekarski, Nicole; University Hospital of Lausanne, Paediatric Cardiology Unit, Woman-Mother-Child Department<br>Messerli, Nadine; University of Fribourg, Clinical Child Psychology & Biological Psychology<br>Ehlert, U; University of Zurich, Department of Clinical Psychology and Psychotherapy<br>Bickle-Graz, Myriam; University Hospital of Lausanne, Neonatology Service, Woman-Mother-Child Department<br>Morisod Harari, Mathilde; University Hospital of Lausanne, Service of Child and Adolescent Psychiatry,<br>Porcheret, Kate; Monash University, Turner Institute for Brain and Mental Health<br>Schechter, Daniel; University Hospital of Lausanne, Service of Child and Adolescent Psychiatry; University of Geneva Faculty of Medicine, Department of Psychiatry<br>Ayers, Susan; INTO City University London, Centre for Maternal and Child Health Research, School of Health Sciences<br>Holmes, Emily; Uppsala University, Department of Psychology<br>Horsch, Antje; University of Lausanne Faculty of Biology and Medicine, Institute of Higher Education and Research in Healthcare; University Hospital of Lausanne, Neonatology Service, Woman-Mother-Child Department,
**Primary Subject Heading**:	Mental health
Secondary Subject Heading:	Obstetrics and gynaecology, Evidence based practice, Reproductive medicine

Keywords:	early intervention, PTSD, maternal mental health, infant development, cortisol, HRV

SCHOLARONE™
Manuscripts

Improving mental health and physiological stress responses in mothers following traumatic childbirth and in their infants: Study protocol for the Swiss TrAumatic biRth Trial (START)

Vania Sandoz[1], Camille Deforges[1], Suzannah Stuijfzand[1], Manuella Epiney[2], Yvan Vial[3], Nicole Sekarski[4], Nadine Messerli, U Ehlert[6], Myriam Bickle-Graz[7], Mathilde Morisod Harari[8], Kate Porcheret[9], Daniel S. Schechter[10,11], Susan Ayers[12], Emily A. Holmes[13], & Antje Horsch[1,7]* on behalf of the *START Research Consortium*

[1]Institute of Higher Education and Research in Healthcare (IUFRS), University of Lausanne, Lausanne, Switzerland

[2]Department Woman-Child-Adolescent, Geneva University Hospital and University of Geneva, Geneva, Switzerland.

[3]Obstetrics and Gynecology Service, Woman-Mother-Child Department, Lausanne University Hospital and University of Lausanne, Lausanne, Switzerland

[4]Paediatric Cardiology Unit, Woman-Mother-Child Department, Lausanne University Hospital and University of Lausanne, Lausanne, Switzerland

[5]Clinical Child Psychology & Biological Psychology, University of Fribourg, Fribourg, Switzerland

[6]Department of Clinical Psychology and Psychotherapy, University of Zurich, Zurich, Switzerland

[7]Neonatology Service, Woman-Mother-Child Department, Lausanne University Hospital and University of Lausanne, Lausanne, Switzerland

[8]Service of Child and Adolescent Psychiatry, Lausanne University Hospital and University of Lausanne, Lausanne, Switzerland

For peer review only - http://bmjopen.bmj.com/site/about/guidelines.xhtml

[9]Turner Institute for Brain and Mental Health, Monash University, Australia

[10]University Hospital of Lausanne, Service of Child and Adolescent Psychiatry Lausanne, Lausanne, Switzerland

[11]Department of Psychiatry, University of Geneva Faculty of Medicine, Geneva, Switzerland

[12]Centre for Maternal and Child Health Research, School of Health Sciences, City, University of London, London, UK

[13]Department of Psychology, Uppsala University, Uppsala, Sweden

Email addresses:

Vania.Sandoz@bluewin.ch

Camille.Deforges@chuv.ch

Suzannah.Ravenscroft@chuv.ch

Manuella.Epiney@hcuge.ch

Yvan.Vial@chuv.ch

Nicole.Sekarski@chuv.ch

Nadine.Messerli@unifr.ch

U.Ehlert@psychologie.uzh.ch

Myriam.Bickle-Graz@chuv.ch

Mathilde.Morisod@chuv.ch

Kate.Porcheret@monash.edu

Daniel.Schechter@chuv.ch

Susan.Ayers.1@city.ac.uk

emily.holmes@psyk.uu.se

Antje.Horsch@chuv.ch

For peer review only

***Corresponding author:**

Prof. Antje Horsch, Institute of Higher Education and Research in Healthcare (IUFRS), University of Lausanne, SV-A, Route de la Corniche 10, 1010 Lausanne.

Phone: 00 41 21 314 85 71. Antje.Horsch@chuv.ch

KEYWORDS

early intervention, PTSD, maternal mental health, infant development, HRV, cortisol, sleep, Bayley-III, trauma, mental imagery, intrusive memories

For peer review only

ABSTRACT

Introduction

Emergency caesarean section (ECS) qualifies as a psychological trauma, which may result in postnatal posttraumatic stress disorder (PTSD). Maternal PTSD may not only have a significant negative impact on mother-infant interactions, but also on long-term infant development. The partner's mental health may also affect infant development. Evidence-based early interventions to prevent the development of postpartum PTSD in mothers are lacking. Immediately after a traumatic event, memory formation is vulnerable to interference. There is accumulating evidence that a brief behavioural intervention including a visuospatial task may result in a reduction in intrusive memories of the trauma.

Methods and analysis

This study protocol describes a double-blind multi-centre randomised controlled phase III trial testing an early brief maternal intervention including the computer game "Tetris" on intrusive memories of the ECS trauma (1 week) and PTSD symptoms (6 weeks, primary outcome) of 144 women following an ECS. The intervention group will carry out a brief behavioural procedure including playing Tetris. The attention-placebo control group will complete a brief written activity log. Both simple cognitive tasks will be completed within the first 6 hours following traumatic childbirth. The intervention is delivered by midwives/nurses in the maternity unit.

The primary outcome will be differences in the presence and severity of maternal PTSD symptoms between the intervention and the attention-placebo control group at 6 weeks postpartum. Secondary outcomes will be physiological stress and psychological vulnerability, mother-infant interaction, and infant developmental outcomes. Other outcomes will be

For peer review only - http://bmjopen.bmj.com/site/about/guidelines.xhtml

psychological vulnerability and physiological regulation of the partner and their bonding with the infant, as well as the number of intrusive memories of the event.

Ethics and dissemination

Ethical approval was granted by the Human Research Ethics Committee of the Canton de Vaud (study number 2017-02142). Dissemination of results will occur via national and international conferences, in peer-reviewed journals, public conferences, and social media.

Trial registration number

clinicaltrials.gov (NCT 03576586)

For peer review only

ARTICLE SUMMARY

Strengths and limitations of this study

- This multi-centre RCT will test the effects of an early brief behavioural intervention carried out by midwives/nurses on the labour ward.
- The primary outcome will be the presence and severity of maternal PTSD symptoms at 6 weeks.
- The early brief intervention, conducted in the same hospital where the traumatic event occurred, includes a visuospatial task aimed at competing with sensory aspects of the traumatic memory before it has been fully consolidated.
- Methodological rigour, including a double-blind design, an active control group, concealment of random allocation, regular monitoring, and prospective trial registration, limits risk of bias.
- Some outcomes are measured with self-report questionnaires, which may induce a social desirability bias.

For peer review only - http://bmjopen.bmj.com/site/about/guidelines.xhtml

ABBREVIATIONS

ANS: Autonomic Nervous System / ASD: Acute Stress Disorder / ASDS: Acute Stress Disorder Scale / BSID-III: Bayley Scales of Infant Development, 3rd Edition / CAPS: Clinician-Administered PTSD Scale for DSM-5 / CAR: Cortisol Awakening Response / CRIB: Clinical Risk Index for Babies / EAS: Emotional Availability Scales / ECG: Electrocardiography / ECS: Emergency Caesarean Section / EPDS: Edinburgh Postnatal Depression Scale / FFSF: Face-to-Face Still-Face paradigm / HADS: Hospital Anxiety and Depression Scale / HPA: Hypothalamic-Pituitary-Adrenal / HRV: Heart Rate Variability / IBIs: Interbeat Intervals / IBQ-R: Infant Behavior Questionnaire-Revised / MEQ: Morningness-Eveningness Questionnaire / MIBS: Mother-to-Infant Bonding Scale / MOS-8: Modified Medical Outcomes Study Social Support Survey / NBAS: Neonatal Behavioural Assessment Scale / PCL-5: PTSD Checklist for DSM-5 / PDPSI: Post-delivery Perceived Stress Inventory / PPSI: Postnatal Perceived Stress Inventory / PSI-SF: Parenting Stress Index - Short Form / PSQI: Pittsburgh Sleep Quality Index / PSQI-A: Pittsburgh Sleep Quality Index Addendum / PTSD: Post-Traumatic Stress Disorder / RCT: Randomised Controlled Trial / RDAS: Revised Dyadic Adjustment Scale / START: Swiss Traumatic Birth Trial

For peer review only

For peer review only - http://bmjopen.bmj.com/site/about/guidelines.xhtml

INTRODUCTION

Childbirth and PTSD

Though childbirth is a common and often fulfilling event, one third of mothers rate their childbirth as traumatic[1]. Childbirth can meet diagnostic criteria for a traumatic event, if women perceived their life and/or the life of their baby to be in danger[2]. Posttraumatic stress disorder (PTSD) related to childbirth is diagnosable in around 3-4% of women[3 4]. PTSD consists of four symptom clusters (intrusions, avoidance, hyperarousal, and negative cognitions and mood) and can be diagnosed at least one month after the traumatic stressor occurred[2]. Comparing different modes of childbirth, obstetric complications, such as emergency caesarean section (ECS) produce higher rates of postnatal PTSD (19-39%)[3]. ECS is a relatively frequent event, and there is thus a need to better identify and support women who are vulnerable to developing PTSD following an ECS.

Postnatal PTSD can significantly influence the experience of subsequent pregnancies, with increased risk of maternal stress and its associated risks of intrauterine growth retardation, premature birth and low birth weight[5-7]. It can lead to a fear of subsequent pregnancy and childbirth, sexual problems, and avoidance of medical care[8 9]. Postnatal PTSD can also have important negative consequences for breastfeeding, the attachment relationship with the baby and mother-infant interactions, with a subsequent detrimental impact on the development of the child, as well as for the couple relationship[7 10-13]. PTSD is also highly co-morbid with depression, for which there is substantial evidence of long-term negative effects on child development and behaviour[14-16]. Estimated economic costs of perinatal mental health problems are about £8.1 billion for each one-year cohort of UK births, of which 72% relate to adverse impacts on the child rather than the mother[17]. In Switzerland, 16.7% of women in the perinatal period used mental health services[18]. New and innovative evidence-based

For peer review only - http://bmjopen.bmj.com/site/about/guidelines.xhtml

interventions are therefore needed to reduce those costs by preventing the development of postnatal PTSD[19].

To date, research investigating PTSD symptoms in partners following ECS is missing. Most studies on partners so far have focused on postnatal depression reporting that 1-8% experience depression symptoms in the first 6 weeks and 5-6% at 3-6 months following childbirth without complications[20-22], with increased risk following high risk situations[23]. In one study, 5% of partners reported severe intrusions and avoidance symptoms at 9 weeks postpartum[24]. Although the influence of partner mental health is understudied, it also seems to negatively impact child outcomes[25-27]. Thus, partner mental health needs to be better understood not only in order to help the partner but also to support family and child outcomes.

Physiological stress responses associated with PTSD

Traumatic exposure activates the hypothalamic-pituitary-adrenal (HPA) axis, a cascade-like hormonal system resulting in the release of cortisol from adrenal cortex cells in body fluids. In parallel, the organism activates the more rapidly mobilizing autonomic nervous system (ANS) resulting in the release of norepinephrine from nerve terminals of the sympathetic nervous system as well as epinephrine and norepinephrine from the adrenal medulla[28]. While the HPA axis shows the above stress-related reactivity, it also shows a basal activity with circadian variations in the respective hormones. For instance, cortisol peaks 30-45 minutes after awakening (the so-called CAR: cortisol awakening response) and gradually declines throughout the day with lowest levels early during sleep[29 30].

Specific patterns of HPA axis functioning have been shown in PTSD[31] although this has so far not been studied after traumatic childbirth. While studies indicate that individuals with PTSD show different patterns of HPA axis functioning to those without PTSD, there is little consistency in the specificity of these patterns[32-38]. A meta-analysis examining diurnal

For peer review only - http://bmjopen.bmj.com/site/about/guidelines.xhtml

cortisol levels in adults with PTSD showed that low cortisol levels were not related to PTSD in general, but rather to trauma exposure and co-morbidities[39]. Finally, a recent study in a postnatal population found a negative association between symptoms of re-experiencing and diurnal cortisol slopes in mothers of preterm children[40]. Concerning cortisol reactivity to a subsequent stressor, again, results are discrepant[41 42].

Although HPA axis reactivity following stress or trauma is thought to be adaptive, acute or chronic exposure to stress has been shown to have deleterious effects[43 44]. It may not only result in dysfunctions of HPA re-activity, but also in health-relevant changes in the basal activation of this system[45]. Overall, these studies show the strong implication of the HPA axis dysregulation in the development and maintenance of PTSD, although studies in postnatal populations are scarce.

Reduced heart rate variability (HRV), an indicator of autonomic flexibility, has been found to be related to psychopathological processes[46]. Individuals with PTSD show lower levels of HRV in comparison with trauma-exposed individuals without PTSD or healthy controls[47 48]. However, the relationship of PTSD and HRV has so far not been studied in a postnatal population.

Sleep in PTSD is also disrupted. Sleep disturbance (i.e., difficulty falling or staying asleep) and recurrent distressing dreams are both diagnostic criteria for PTSD[2], with 70-91% of patients with PTSD suffering from subjective sleep disturbances and 19-71% reporting nightmares[49]. Findings from experimental research indicate that sleep on the first night after trauma may be important for the development of subsequent intrusive memories of the index trauma. One study found that totally sleep deprived participants reported *fewer* intrusive memories after a laboratory stressor compared to those who slept[50], though findings are mixed[51-53]. Thus, it is important to assess sleep over time following a real world traumatic

For peer review only - http://bmjopen.bmj.com/site/about/guidelines.xhtml

event, such as following an ECS. To date, no studies to our knowledge have examined sleep in postpartum PTSD.

Maternal PTSD and infant physiological stress responses

Maternal PTSD and its associated dysfunction of the HPA axis can also impact on the stress regulation of the offspring[54-63], such as the infant's HPA secretion patterns[64-66]. A growing body of neuroendocrine research supports the notion that an altered maternal HPA axis functioning plays a role in the intergenerational transmission of stress-related psychopathology from parents[67 68]. Overall, findings suggest that PTSD symptoms and cortisol levels in mothers are important to assess, prevent and/or treat as they may affect the relationship with the infant[69-72] and impact the child's later regulative abilities[54]. Some authors have suggested low maternal cortisol as a possible mechanism contributing to the mother's difficulty in sensitively attuning to her infant's cues, which in turn impacts on the infant's reactivity to and recovery from a stressor[73-78].

In contrast, studies assessing the role of ANS in the intergenerational transmission of stress in the postpartum period are so far scarce. Lifetime maternal psychopathology and maternal postnatal psychopathology have been found to be related with reduced HRV of their infants[79]. Furthermore, mothers with anxiety symptoms during pregnancy and their infants showed lower HRV[80] and there was a higher sympathetic activation in children of mothers with abuse histories[81]. However, to our knowledge none of the previous research has investigated the autonomic functioning in offspring of mothers with PTSD.

Developing an early intervention inspired by behavioural and cognitive neuroscience

To date, there is a lack of evidence-based early interventions for women following a traumatic childbirth[82]. At the heart of PTSD are intrusive memories of the traumatic event, in

For peer review only - http://bmjopen.bmj.com/site/about/guidelines.xhtml

which the person re-experiences aspects of the traumatic event, inflicting significant distress[2]. They have also been indicated as a precursor to the disorder[83]. Intrusive memories of trauma comprise sensory-perceptual images that are proposed to occur due to excessive perceptual (sensory) processing during a trauma[84].

The way in which individuals process a traumatic event influences their later intrusive memories of the trauma. Evidence from lab-based experiments have demonstrated that a brief behavioural intervention including a reminder cue, mental rotation and a visuospatial task (Tetris) can significantly reduce the frequency of intrusive images following exposure to traumatic film material[85 86]. One study showed that individuals who were instructed to engage in conceptually-driven processing, relative to those engaged in sensory-based, data-driven processing, reported more intrusive memories to a traumatic film[87]. It has been hypothesised that tasks which interfere with data-driven processing, such as sensory-perceptual, visuospatial tasks, may reduce the occurrence of intrusive memories of an index event[85 86]. Visuospatial cognitive tasks, such as the computer game Tetris, are thought to compete for resources with visuospatial images[88]. Studies of memory consolidation have shown that human memories are likely to still be malleable within 10 min to 6 hours, at which point the memory is thought to stabilise consolidation, making it more resistant to interference from a competing memory[89-91]. This indicates that such a working memory task may be most beneficial if delivered within approximately the first 6 hours following a traumatic event (see[92] for a review).

Two recent translational studies presented preliminary evidence for the efficacy of a brief intervention (including Tetris) in reducing the number of traumatic intrusive memories (over 1 week post-trauma) in patients arriving at an accident and emergency department (vs. attention placebo)[93] or in women in the first hours following ECS, the latter when compared with a treatment-as-usual control group[4]. In the latter study, per protocol analyses also showed

For peer review only - http://bmjopen.bmj.com/site/about/guidelines.xhtml

significantly lower re-experiencing symptoms at 1 week and lower rates of PTSD diagnosis at 1 month following ECS (secondary outcomes)[4].

Aims of the present study

The objectives of the present study are to investigate the effects of an early brief, behavioural intervention (including the computer game Tetris) delivered in the hospital context within 6 hours of the trauma, on maternal mental health and infant development after a traumatic event (ECS). The primary outcome measure will be the presence and the severity of maternal PTSD symptoms at 6 weeks. Secondary objectives will be to measure the impact of this intervention on intrusive memories of the trauma, on stress exposure and perception, on other indicators of maternal psychological vulnerability (including acute stress disorder (ASD), PTSD, anxiety, depression, and sleep), on physiological stress reactivity, on physiological regulation, on mother-infant interactions, and on infant development. The START study also aims to investigate additional maternal and partner psychological vulnerability, partner physiological regulation, partner-infant bonding, and measures related to the acceptability and expectancy of the intervention.

METHODS

Study Design

We will conduct a multi-centre double-blind randomised controlled trial (RCT) with minimal risk testing the effect of an early brief behavioural intervention including computer game play for women at risk of PTSD in the hospital soon following an ECS and their infant, compared with an attention-placebo control task.

For peer review only - http://bmjopen.bmj.com/site/about/guidelines.xhtml

Study population, recruitment, group allocation, and blinding

All women who have an ECS ≥34 weeks gestation, give birth to a live baby, and give written consent are eligible to participate. In addition, they have to answer with a score of ≥2 separately for at least two out of four screening questions regarding perceived threat[94]. All screening questions are answered on a 7-point Likert scale (*1 = not at all, 7 = extremely*): Did you think that your life was in danger? Did you think that your baby's life was in danger? Did you feel frightened during the birth? Did you feel helpless during the birth?

Exclusion criteria include: established intellectual disability or psychotic illness, insufficient French-speaking level to participate in assessments, severe illness of mother or infant (e.g., cancer, cardiovascular disease, severe neurodevelopmental difficulties, malformations) or if the infant requires intensive care, and alcohol abuse and/or illegal drug use during pregnancy.

Following an ECS and once the mother has sufficiently recovered, the mother's midwife/nurse will assess eligibility and if eligible, will inform the mother about the study. After providing written and informed consent to be screened, participants will be screened immediately for perceived threat of the mother and/or child with four screening questions. If participants score ≥2 separately for at least two out of the four questions, they will be randomly assigned to either the intervention or attention-placebo control group. We aim to recruit 144 women and their infants (see sample size calculation).

If the woman agrees, then the partner will also be informed about the study. Inclusion criteria for the partners are that they were present at the childbirth and give written consent. Partners are excluded if they do not speak French sufficiently well to participate in assessments. All participants will be reimbursed for their time and effort.

The allocation ratio of randomisation is 1:1. The randomization sequence will be generated using a computer-generated block randomisation (StataCorp. 2017. *Stata Statistical*

For peer review only - http://bmjopen.bmj.com/site/about/guidelines.xhtml

Software: Release 15. College Station, TX) using blocks of sizes 2, 4 and 6 over 144 participants per stratum (stratified by research centre). Opaque envelopes will be prepared in advance, numbered sequentially by alternating between stratums by block. After conducting the baseline assessment, the clinical midwife/nurse will open the envelope and announce the cognitive task to be carried out (i.e., the cognitive visuospatial task or the attention-placebo control task) to the participant[95]. All members of the research group as well as participants are blind to group allocation. All participant data will be coded to ensure confidentiality. After completing the 15-minute cognitive tasks, both women of the control and intervention group will complete the same assessments, as shown in Table 1; see Figure 1 for an overview of the study variables.

Intervention group

Mothers in the intervention group will be instructed to engage in a cognitive visuospatial task, the computer game Tetris, for 15 minutes continuously, on a handheld gaming device (Nintendo DS) (see[4]). They are given a 3-min training in how to play the game and how to actively use mental rotation as they play the game. The intervention is delivered in the same context as that in which the trauma occurred (e.g,, wake-up room) so additional memory reminder cue to the trauma is not used.

The intervention will take place within the first six hours after ECS whilst participants are still in their hospital bed. Procedures are managed not to interfere with important routine care procedures. An unblinded independent researcher will check during the days following the intervention that the intervention protocol was followed correctly via a 4-item survey completed by the participant. Study information and materials refer to 'simple tasks' in both conditions for credibility.

For peer review only - http://bmjopen.bmj.com/site/about/guidelines.xhtml

Attention-placebo control group

Mothers assigned to the control group will be asked to engage in a written activity log for 15 minutes (based on previous research[93]). They are instructed to write down very briefly nature and duration of the activities (e.g., "*being with baby for 10 min*", "*phone call for 5 min"*) and they are instructed not to sleep. The activity log was selected to control for nonspecific confounding factors, while minimizing the potential for harmful effects, as to date, no preventive treatment in the immediate aftermath of trauma exists that could be used as a control condition[96-99]. This control condition was matched with the intervention condition for nonspecific factors including length of the task, contact with the midwife/nurse, location of treatment procedure and engagement in a structured task[93]. The attention-placebo control task will take place within the six hours after ECS, whilst participants are still in their hospital bed and will not interfere with important routine care procedures. An unblinded independent researcher will check during the days following childbirth that the attention-placebo control task protocol was followed correctly via a 2-item survey completed by the participant. Study information and materials refer to 'simple tasks' in both conditions for credibility.

Primary outcome

The primary outcomes are differences in the presence and severity of maternal PTSD symptoms between the intervention and the attention-placebo control group at 6 weeks postpartum measured with the Clinician-administered PTSD Scale[100] and the PTSD Checklist[101].

Secondary outcomes

The following outcomes will be assessed via validated assessments at different time-points (i.e., ≥6 hours following ECS, ≤1 and 6 weeks, and 6 months follow-up).

For peer review only - http://bmjopen.bmj.com/site/about/guidelines.xhtml

Maternal outcomes

The maternal mental health outcomes will be compared between the two groups at all time-points, including number of intrusive memories of the index trauma (at ≤1 week follow-up) and indicators of maternal psychological vulnerability namely: symptoms of ASD, PTSD, anxiety, depression, sleep and physical activity (at ≥6 hours following ECS, ≤1 and 6 weeks, and 6 months follow-up) (see Table 1). Additional physiological outcomes will be collected in reactivity to stress and as regulation indicators (at 6 weeks and 6 months follow-up). Finally, maternal bonding and sensitivity in mother-infant interaction will also be measured (at ≤1 and 6 weeks, and 6 months follow-up).

Child outcomes

As shown in Table 1, infant development will be assessed at 6 months postpartum. Additionally, physiological outcomes will be assessed in response to stress and as regulation indicators (at 6 weeks and 6 months follow-up).

Other outcomes

Additional measures of maternal and partner psychological vulnerability (at ≤1 and 6 weeks, and 6 months follow-up), partner infant interaction (at ≤1 and 6 weeks, and 6 months follow-up), infant neurodevelopmental vulnerability (at ≤1 week), medical outcomes (at ≤1 and 6 weeks, and 6 months follow-up), and measures related to the acceptability and expectancy of the intervention (at ≥6 hours following ECS) are described in Table 2.

Data collection and visits

For peer review only - http://bmjopen.bmj.com/site/about/guidelines.xhtml

Figure 2 summarises study procedures and Table 1 indicates the measures collected at each time point.

Measures

Measures of the primary and secondary outcomes can be found below, measures relating to the ‘other outcomes’ in the study can be found in Table 2. The time points of when all measures are taken can be seen in Table 1.

For peer review only

Psychological Vulnerability

Clinician-administered PTSD Scale (CAPS-5)[100]. The CAPS-5 assesses presence and severity of PTSD symptoms and diagnosis. This gold standard instrument contains 20 items referring to the four symptom clusters, as well as 10 items referring to symptoms duration, distress or impairment, global ratings, and dissociative subtype. Each diagnostic criterion is rated from *0=absent* to *4=extreme/incapacity* in function of symptoms intensity and frequency with a diagnostic cut-off equal to 2. The CAPS-5 has demonstrated good psychometric proprieties[100]. In the absence of a French version at the time when the study was designed, a forward-back method was executed to realise a translation and cultural adaptation[102].

The PTSD Checklist (PCL-5)[101]. This 20-item self-report questionnaire measures symptoms of PTSD over the past month and is used to assess frequency of PTSD symptoms. The PCL-5 refers to the four symptoms clusters of PTSD and scales on a 5-point scale with *0=not at all* and *4=extremely*. Scores are summed to create a total symptom severity score [103]. The French version of the PCL-5 demonstrated strong reliability and validity[104].

Trauma-related intrusive memories diary[4]. Intrusive memories of birth-related trauma experienced during the seven days following ECS are reported in a daily diary, adapted from

For peer review only - http://bmjopen.bmj.com/site/about/guidelines.xhtml

previous work[4], to assess the frequency of intrusive memories of the trauma . For each intrusion, the time, content and type (intrusive memory, nightmare or other) are recorded, as well as the level of distress on a 5-point scale with ratings of *0=not at all* to *5=extremely.*

Acute Stress Disorder Scale (ASDS)[105]. This self-assessment instrument measures frequency of ASD symptoms over the last week and is based on DSM 5[106]. Each of the 19 items is scored using a scale from *1=not at all* to *5=extremely*, with a higher score indicating higher ASD symptoms. Good sensitivity and specificity has been reported[105]. The forward-back method was executed to realise a French version translation and cultural adaptation[102].

Anxiety subscale of Hospital Anxiety and Depression Scale (HADS)[107]. This self-report questionnaire measures severity of anxiety symptoms during the last week. The anxiety subscale consists of 7 items scored on a 4-point scale (*0=never*, *3=most of the time*). Higher scores indicate higher distress. Good psychometric properties have been reported for the French version[108].

Edinburgh Postnatal Depression Scale (EPDS)[109]. This self-assessment examines postnatal depression symptoms over the previous week[109]. The 10 items are scored on a 4-point scale and scores range from 0 to 30. Higher scores indicate higher distress. The French version has demonstrated good psychometric proprieties[110]. A clinical cut-off score of 10.5 has been reported for the use of the French validated version[110].

Physical and sleep activity. Frequency and duration of maternal sleep and physical activity of the 5 days following childbirth is measured using an accelerometer watch GENEActiv®[111].

Sleep diary. Mothers record their hours of sleep during the week following ECS in a daily sleep diary.

Pittsburgh Sleep Quality Index (PSQI)[112]. This 19-item questionnaire measuring sleep during the past month is composed of seven sleep quality-related subscales. The overall score

For peer review only

assessing sleep quality is scored by summing the subscales; scores range from 0 to 21. Higher results indicate poor sleep quality while a score of >5 distinguishes good and poor sleepers. The validated French version of the PSQI has shown good psychometrics proprieties[113].

We also included the 10 items of the PSQI-A[114] to assess PTSD-specific sleep disturbances over the past month, answered on a four-point Likert scale (0=*not during the past month*; 3=*three or more times a week*), that assess the frequency of different kinds of trauma-related sleep disturbance. The items are summed to create the total score, where the higher the score the more disturbed the sleep. The validated French version of the PSQI-A has shown good psychometrics[114].

Maternal and infant physiological stress responses.

Physiological regulation. Resting heart rate of the mother and infant are assessed using resting HRV measured by Firstbeat Bodyguard 2 devices providing a continuous measure of cardiac activity. Resting heart rate will be assessed during the 15 minutes resting period before the stress paradigms (see *Physiological stress reactivity* for stress paradigms) at ≤1 week and 6 months. Baseline salivary cortisol and cortisol daily profile will be established for the mother in the two days after leaving the maternity ward (usually the 6th and 7th day postpartum) and for her and her baby for two days at 6 months through salivary sample; 5 saliva samples are taken per day, including CAR. Maternal salivary cortisol will be collected using Salivettes© Sarstedt (item number: 51.1534.500) and SalivaBio Infant's Swab (Salimetrics, item number: 5001.08 50) for infants.

Physiological stress reactivity. Maternal and infant stress reactivity will be assessed via salivary cortisol and HRV using Firstbeat Bodyguard 2 devices during the stress phases of their respective stress paradigms at 6 months for the mother and at ≤1 week and at 6 months for the infant. The stress paradigm for the mothers is the Trier Social Stress Test (TSST)[115] at

For peer review only - http://bmjopen.bmj.com/site/about/guidelines.xhtml

6 months. Maternal salivary cortisol will be measured 7 times before, during, and after the stress paradigms and heart rate throughout the stress paradigm. Stress paradigms for the infants involve the Neonatal Behavioural Assessment Scale (NBAS)[116] at ≤1 week and the double-exposure Face-to-Face Still-Face paradigm (FFSF)[117-120] at 6 months. Salivary cortisol will be measured 3 times, once before and twice after the stressor, and HRV will be measured throughout the stress paradigms. Maternal cortisol will also be collected during the FFSF. Maternal salivary cortisol will be collected using Salivettes© Sarstedt and SalivaBio Infant's Swab (Salimetrics) for infants. Participation in maternal stress reactivity assessments at ≤1 week is optional (involving an additional consent obtained before hospital discharge).

Mother-infant interaction

Mother-to-Infant Bonding Scale (MIBS)[121]. This 8-item questionnaire assesses the mothers' feelings towards her new baby in the first few weeks after birth. Eight adjectives are rated on a scale from *0=very much* to *5=not at all*. Scores are summed to create a total score, with a higher score indicating worse mother-to infant bonding. The MIBS has shown good initial psychometrics[121], and has been validated in French[122].

Emotional Availability Scale (EAS)[123 124]. Maternal sensitivity and responsiveness will be investigated during a free-play session of mothers with their six-months old. Interactions are coded on six dimensions: sensitivity, structuration, intrusion, hostility toward the infant, reactivity to the mother, and maternal involvement[123 124]. Higher scores on each scale indicate better performance. Two trained psychologists blind to the condition will rate each interaction and inter-observer reliability will be calculated. The EAS shows good psychometric properties[123 124].

For peer review only - http://bmjopen.bmj.com/site/about/guidelines.xhtml

Infant developmental outcomes.

Infant Behaviour Questionnaire – Revised (IBQ-R) Very Short Form[125]. This is a parent-report questionnaire consisting of 36 items answered on a 7-point Likert-scale (*1=never* to *7=always*). The items assess the frequency of infants' behaviours during the previous two weeks to measure child temperament. The very short form has shown good psychometric properties[125]. There was no available validation French translation. Therefore, the forward-back method was completed to realise a French version translation and cultural adaptation[102]

Bayley Scales of Infant Development (Bayley-lll)[126]. The cognitive, language and motor subscales of the Bayley Scales of Infant Development assess the developmental functioning of the infant. The scales are administered by a trained psychologist or paediatrician through a standard set of play tasks following a standardized protocol. The composite scores for the subscales are age-standardized with a mean score of 100[127].

Sociodemographic, obstetric and neonatal characteristics

Mothers will report demographic information, including marital status, nationality, profession, level of education[128], and previous and current psychiatric disease as well as any trauma history via a self-report questionnaire. Mothers will also report their height, weight (before pregnancy and current), menstrual cycle, smoking behaviours, and alcohol/drug use. Obstetric data will be extracted from the hospital medical record, such as pregnancy-, labour- and birth-related information, gravidity, parity, mode of previous childbirths, history of miscarriage, stillbirth, and prematurity, pain, medication, birth control, sexual activity, and psychological support. Neonatal characteristics will be collected from the medical record on severity of morbidity (gestational age and weight at birth, Apgar score, neonatal

For peer review only - http://bmjopen.bmj.com/site/about/guidelines.xhtml

complications), as well as the Clinical Risk Index for Babies (CRIB II)[129], which represents neonatal morbidity severity.

Sample size calculation

Based on a previous proof-of-principle RCT[4], a sample size of n=120 participants is necessary to have 80% power (α=0.05) to detect a between-group difference of d=0.30 of the primary outcome (PTSD symptoms at 6 weeks). Furthermore, we calculated the sample sizes necessary to obtain significant group differences with 80% power (α=0.05) based on small effect sizes (d<0.30) for maternal secondary outcomes (PTSD diagnosis, ASD symptoms and physiological stress reactivity) and infant secondary outcomes (physiological stress reactivity) based on small effect sizes (d<0.30) with 80% power (α=0.05), which range between a total of n=56 and n=84. Predicting a 20% drop-out rate, we aim to recruit 144 women.

PATIENT AND PUBLIC INVOLVEMENT

The acceptability of the intervention was assessed by participants during the previous proof-of-principle RCT[4]. A questionnaire of satisfaction and acceptability examined the burden of the intervention. An individual feedback and debriefing session will be offered to each participant. Results will be disseminated in written form to the participants and distributed to the public via social media and public events. They will also be discussed with clinical professionals involved in this project.

DATA MANAGEMENT AND STATISTICAL ANALYSES

All study data will be entered by research staff. Study data will be managed using REDCap electronic data capture tools hosted at Lausanne University Hospital[130 131]. REDCap (Research Electronic Data Capture) is a secure, web-based software platform designed to

For peer review only - http://bmjopen.bmj.com/site/about/guidelines.xhtml

support data capture for research studies. Double data entry will be done for the primary outcomes. For the rest of the data, a random 5% will be double-checked. Access to the full final trial dataset will be restricted to the principal investigator.

For the primary analyses, group differences regarding the mean subscale and total scores of the PCL-5 and CAPS-5 at 6 weeks will be analysed using separate linear regression analyses. Analyses will be adjusted for recruitment centre and the respective baseline values. Associations between primary outcomes and potential confounders, such as maternal and gestational age, will first be assessed applying univariate tests. Subsequent analyses will be adjusted for significant covariates. For secondary aims, the analyses will be performed for differences in changes between groups and differences between groups at different time points in maternal and infant outcomes. Proportion of participants meeting the diagnostic criteria for PTSD at 6 weeks and 6 months between the two groups will also be compared using logistic regression analyses. The same procedure for identifying significant covariates described above will be applied here. Furthermore, post-hoc exploratory analyses will be conducted but described as such in publications. All regression analyses regarding cortisol data will be adjusted for potential covariates, such as co-sleeping, breastfeeding, menstrual cycle, and infant and maternal medication. For HRV analyses interbeat intervals (IBIs) from baseline, stress task and recovery will be analysed by the extraction from ECG recordings. Statistical parameters of HRV[132] will be calculated using Kubios HRV Analysis2.2 software[133]. Time domain measures and spectral frequency measures will be used for calculations. Multiple imputation methods will be used to manage missing data, if appropriate.

ETHICAL APPROVAL

For peer review only - http://bmjopen.bmj.com/site/about/guidelines.xhtml

This study protocol was approved by the Human Research Ethics Committee of the Canton de Vaud (Switzerland, study number 2017-02142, protocol version 5 of September 13rd 2019) and is registered in *clinicaltrials.gov* (NCT03576586). Substantial amendments will only be implemented after approval of the ethics committee, and all non-substantial amendments are communicated to the ethics committee within the Annual Safety Report. Participating mothers and, where included, partners will provide their written informed consent before their enrolment in the study by the clinical and research staff. The written informed consent contains an optional part on the re-use of data in ancillary studies. Participation will not interfere with typical care provided to patients after an ECS. The scientific conduct of the study is overseen by an interdisciplinary steering committee of national and international experts. Confidentiality will be ensured by utilising subject identification code numbers to correspond to treatment data in the computer files.

ACKNOWLEDGMENTS

We thank Pierluigi Ballabeni for his statistical advice and the generation of the randomization sequence. We also thank our interns for help with study co-ordination and data collection: Lara Schütz, Anna Favero, Yasmina Lotfi, Mathilde Duroux, Cassie Pernet, Daniela Barros Rodrigues, Mélanie Hausser, and Annabelle Reuse. For institutional support, access to the study population, help with recruitment, data collection, and administrative support, we acknowledge Laetitia Accrombessi, Danièle Losguardi, Pascale Gerdy, Maria-Teresa Adjaho, Lauretta Monney, Nadia Carota, Annette Schauenberg, Laetitia Favre, Antonina Chilin, Céline Favrod, and all clinical teams of the labor and postpartum wards at the Lausanne and Geneva University Hospitals.

COLLABORATORS

For peer review only - http://bmjopen.bmj.com/site/about/guidelines.xhtml

The following are members of the *START Research Consortium* (including the authors of the present article), listed in alphabetical order:

- Valérie Avignon (Woman-Mother-Child Department, Lausanne University Hospital and University of Lausanne, Lausanne, Switzerland; valerie.avignon@chuv.ch),
- Susan Ayers (Centre for Maternal and Child Health Research, School of Health Sciences, City, University of London, London, UK; susan.ayers.1@city.ac.uk),
- Myriam Bickle-Graz (Neonatology Service, Woman-Mother-Child Department, Lausanne University Hospital and University of Lausanne, Lausanne, Switzerland; myriam.bickle-graz@chuv.ch),
- Cristina Borradori Tolsa (Department of Pediatrics, Geneva University Hospitals, Geneva, Switzerland; cristina.borradoritolsa@hcuge.ch),
- Julie Bourdin (Woman-Mother-Child Department, Lausanne University Hospital and University of Lausanne, Lausanne, Switzerland; julie.bourdin@chuv.ch),
- Camille Deforges (Institute of Higher Education and Research in Healthcare (IUFRS), University of Lausanne, Lausanne, Switzerland; camille.deforges@chuv.ch),
- Dominique Delecraz (Department of Woman, Child and Adolescent, Geneva University Hospitals, Geneva, Switzerland; dominique.delecraz@hcuge.ch),
- David Desseauve (Woman-Mother-Child Department, Lausanne University Hospital and University of Lausanne, Lausanne, Switzerland; david.desseauve@chuv.ch),
- Ulrike Ehlert (Department of Clinical Psychology and Psychotherapy, University of Zurich, Zurich, Switzerland; u.ehlert@psychologie.uzh.ch),
- Manuella Epiney (Department of Woman, Child and Adolescent, Geneva University Hospitals, Geneva, Switzerland; manuella.epiney@hcuge.ch),
- Isabelle Eragne (Department of Woman, Child and Adolescent, Geneva University Hospitals, Geneva, Switzerland; Isabelle.Eragne@hcuge.ch),

For peer review only - http://bmjopen.bmj.com/site/about/guidelines.xhtml

- Emily A. Holmes (Department of Psychology, Uppsala University, Uppsala, Sweden; emily.holmes@psyk.uu.se),
- Antje Horsch (Institute of Higher Education and Research in Healthcare (IUFRS), University of Lausanne and Neonatology Service, Woman-Mother-Child Department, Lausanne University Hospital and University of Lausanne, Lausanne, Switzerland; antje.horsch@chuv.ch),
- Justine Imbert (Woman-Mother-Child Department, Lausanne University Hospital and University of Lausanne, Lausanne, Switzerland; justine.imbert@chuv.ch),
- Nadine Messerli-Bürgy (Clinical Child Psychology & Biological Psychology, University of Fribourg, Fribourg, Switzerland; nadine.messerli@unifr.ch),
- Micah M. Murray (Faculty of Biology and Medicine, University of Lausanne, Lausanne, Switzerland; Micah.Murray@chuv.ch),
- Mathilde Morisod Harari (Service of Child and Adolescent Psychiatry, Lausanne University Hospital and University of Lausanne, Lausanne, Switzerland; mathilde.morisod@chuv.ch),
- Kate Porcheret (Turner Institute for Brain and Mental Health, Monash University, Monash University, Melbourne, Australia; kate.porcheret@monash.edu),
- Jardena J. Puder (Woman-Mother-Child Department, Lausanne University Hospital and University of Lausanne, Lausanne, Switzerland; jardena.puder@chuv.ch),
- Véronique Othenin-Girard (Department of Woman, Child and Adolescent, Geneva University Hospitals, Geneva, Switzerland; veronique.othenin-girard@hcuge.ch),
- Suzannah Stuijfzand (Institute of Higher Education and Research in Healthcare (IUFRS), University of Lausanne, Lausanne, Switzerland; suzannah.ravenscroft@chuv.ch),
- Vania Sandoz, M.Sc. (Institute of Higher Education and Research in Healthcare (IUFRS), University of Lausanne, Lausanne, Switzerland; vania.sandoz@chuv.ch),

For peer review only - http://bmjopen.bmj.com/site/about/guidelines.xhtml

- Daniel S. Schechter (Service of Child and Adolescent Psychiatry, Lausanne University Hospital and University of Lausanne, Lausanne, Switzerland, and Department of Psychiatry, University of Geneva Faculty of Medicine, Geneva, daniel.schechter@hcuge.ch),
- Nicole Sekarski (Paediatric Cardiology Unit, Woman-Mother-Child Department, Lausanne University Hospital and University of Lausanne, Lausanne, Switzerland; nicole.sekarski@chuv.ch),
- Jean-François Tolsa (Department of Woman-Mother-Child, Clinic of Neonatology, Lausanne University Hospital and University of Lausanne, Lausanne, Switzerland; jean-francois.tolsa@chuv.ch),
- Yvan Vial (Obstetrics and Gynecology Service, Woman-Mother-Child Department, Lausanne University Hospital and University of Lausanne, Lausanne, Switzerland; yvan.vial@chuv.ch).

AUTHOR CONTRIBUTIONS

AH designed the study with input from all co-authors and members of the consortium. VS, ME, YV, NS, NMB, UE, MBG, MMH, KP, DS, SA and EH participated in the design of the study. AH, VS, CD and SS drafted the manuscript. VS, CD, ME, NMB, MBG, SS, EH and AH significantly contributed to the establishment and refinement of study procedures. All authors critically revised the manuscript and approved the final version of the manuscript.

FUNDING

The START study is funded by a project grant from the Swiss National Science Foundation (SNF 32003B_172982/1).

For peer review only - http://bmjopen.bmj.com/site/about/guidelines.xhtml

COMPETING INTERESTS

None declared.

ETHICAL APPROVAL

Ethical approval was granted by the Human Research Ethics Committee of the Canton de Vaud (study number 2017-02142).

REFERENCES

1. Soet JE, Brack GA, DiIorio C. Prevalence and Predictors of Women's Experience of Psychological Trauma During Childbirth. *Birth* 2003;30(1):36-46. doi: http://dx.doi.org/10.1046/j.1523-536X.2003.00215.x
2. American Psychiatric Association. Diagnostic and statistical manual of mental disorders. 5 ed. Arlington, VA: American Psychiatric Publishing 2013.
3. Yildiz PD, Ayers S, Phillips L. The prevalence of posttraumatic stress disorder in pregnancy and after birth: a systematic review and meta-analysis. *Journal of affective disorders* 2017;208:634-47. doi: https://doi.org/10.1016/j.jad.2016.10.009
4. Horsch A, Vial Y, Favrod C, et al. Reducing intrusive traumatic memories after emergency caesarean section: A proof-of-principle randomized controlled study. *Behaviour research and therapy* 2017;94:36-47. doi: https://doi.org/10.1016/j.brat.2017.03.018
5. Rogal SS, Poschman K, Belanger K, et al. Effects of posttraumatic stress disorder on pregnancy outcomes. *Journal of affective disorders* 2007;102(1-3):137-43. doi: https://doi.org/10.1016/j.jad.2007.01.003
6. Seng JS, Oakley DJ, Sampselle CM, et al. Posttraumatic stress disorder and pregnancy complications. *Obstetrics & Gynecology* 2001;97(1):17-22. doi: https://doi.org/10.1097/00006250-200101000-00004
7. Cook N, Ayers S, Horsch A. Maternal posttraumatic stress disorder during the perinatal period and child outcomes: A systematic review. *Journal of affective disorders* 2018;225:18-31. doi: https://doi.org/10.1016/j.jad.2017.07.045
8. Hofberg K, Brockington I. Tokophobia: an unreasoning dread of childbirth. *The British journal of psychiatry : the journal of mental science* 2000;176(1):83-85. doi: https://doi.org/10.1192/bjp.176.1.83 [published Online First: 2000/05/02]
9. Morland L, Goebert D, Onoye J, et al. Posttraumatic stress disorder and pregnancy health: preliminary update and implications. *Psychosomatics* 2007;48(4):304-08. doi: https://doi.org/10.1176/appi.psy.48.4.304
10. Garthus-Niegel S, Horsch A, Ayers S, et al. The influence of postpartum PTSD on breastfeeding: A longitudinal population-based study. *Birth* 2018;45(2):193-201. doi: https://doi.org/10.1111/birt.12328
11. Garthus-Niegel S, Horsch A, Bickle Graz M, et al. The prospective relationship between postpartum PTSD and child sleep: A 2-year follow-up study. *Journal of affective disorders* 2018;241:71-79. doi: https://doi.org/10.1016/j.jad.2018.07.067
12. Garthus-Niegel S, Horsch A, Handtke E, et al. The Impact of Postpartum Posttraumatic Stress and Depression Symptoms on Couples' Relationship Satisfaction: A Population-

For peer review only - http://bmjopen.bmj.com/site/about/guidelines.xhtml

Based Prospective Study. *Frontiers in Psychology* 2018;9:1728. doi: https://doi.org/10.3389/fpsyg.2018.01728

13. Pisoni C, Spairani S, Fauci F, et al. Effect of maternal psychopathology on neurodevelopmental outcome and quality of the dyadic relationship in preterm infants: an explorative study. *The Journal of Maternal-Fetal & Neonatal Medicine* 2018:1-10. doi: https://doi.org/10.1080/14767058.2018.1487935
14. Grace SL, Evindar A, Stewart D. The effect of postpartum depression on child cognitive development and behavior: a review and critical analysis of the literature. *Archives of Women's Mental Health* 2003;6(4):263-74. doi: https://doi.org/10.1007/s00737-003-0024-6
15. Pearson RM, Bornstein MH, Cordero M, et al. Maternal perinatal mental health and offspring academic achievement at age 16: the mediating role of childhood executive function. *Journal of Child Psychology and Psychiatry* 2016;57(4):491-501. doi: https://doi.org/10.1111/jcpp.12483
16. Sandoz V, Bickle-Graz M, Camos V, et al. Maternal postpartum depression symptoms are negatively associated with emotion regulation of children born very preterm. *Acta paediatrica* 2019;108(5):869-970. doi: https://doi.org/10.1111/apa.14712
17. Bauer A, Parsonage M, Knapp M, et al. The costs of perinatal mental health problems. London: Centre for Mental Health and London School of Economics, 2014.
18. Berger A, Bachmann N, Signorell A, et al. Perinatal mental disorders in Switzerland: prevalence estimates and use of mental-health services. *Swiss Medical Weekly* 2017;147(0910):w14417. doi: https://doi.org/10.4414/smw.2017.14417
19. Horsch A. Post traumatic stress disorder following childbirth and pregnancy loss. In: Beinard H, Kennedy P, S. L, eds. Clinical Psychology in Practice. Oxford: Blackwell, BPS 2009:274-87.
20. Bradley R, Slade P. A review of mental health problems in fathers following the birth of a child. *Journal of reproductive and infant psychology* 2011;29(1):19-42. doi: https://doi.org/10.1080/02646838.2010.513047
21. Bradley R, Slade P, Leviston A. Low rates of PTSD in men attending childbirth: a preliminary study. *British Journal of Clinical Psychology* 2008;47(3):295-302. doi: https://doi.org/10.1348/014466508x279495
22. Lane A, Keville R, Morris M, et al. Postnatal depression and elation among mothers and their partners: prevalence and predictors. *British Journal of Psychiatry* 1997;171(6):550-55. doi: https://doi.org/10.1192/bjp.171.6.550
23. Horsch A, Jacobs I, Gilbert L, et al. Impact of perinatal asphyxia on parental mental health and bonding with the infant: a questionnaire survey of Swiss parents. *BMJ Paediatrics Open* 2017;1(1):e000059. doi: https://doi.org/10.1136/bmjpo-2017-000059
24. Ayers S, Wright DB, Wells N. Symptoms of post-traumatic stress disorder in couples after birth: association with the couple's relationship and parent–baby bond. *Journal of Reproductive and Infant Psychology* 2007;25(1):40-50. doi: https://doi.org/10.1080/02646830601117175
25. Lamb ME. The role of the father in child development. 4th ed. Hoboken, N.J.: John Wiley & Sons 2004.
26. Paulson JF, Keefe HA, Leiferman JA. Early parental depression and child language development. *Journal of Child Psychology and Psychiatry* 2009;50(3):254-62. doi: https://doi.org/10.1111/j.1469-7610.2008.01973.x

For peer review only

For peer review only - http://bmjopen.bmj.com/site/about/guidelines.xhtml

27. Ramchandani P, Psychogiou L. Paternal psychiatric disorders and children's psychosocial development. *The Lancet* 2009;374(9690):646-53. doi: https://doi.org/10.1016/s0140-6736(09)60238-5
28. de Kloet ER, Joels M, Holsboer F. Stress and the brain: from adaptation to disease. *Nature reviews Neuroscience* 2005;6(6):463-75. doi: 10.1038/nrn1683
29. Rohleder N, Nater UM, Wolf JM, et al. Psychosocial stress-induced activation if salivary alpha-amylase. An indicator of sympathetic activity? *Annals of the New York Academy of Sciences* 2004;1032(1):258-63. doi: https://doi.org/10.1196/annals.1314.033
30. Nater UM, Rohleder N, Scholtz W, et al. Determinants of the diurnal course of salivary alpha-amylase. *Psychoneuroendocrinology* 2007;32(4):392-401. doi: https://doi.org/10.1016/j.psyneuen.2007.02.007
31. Ehlert U, Gaab J, Heinrichs M. Psychoneuroendocrinological contributions to the etiology of depression, posttraumatic stress disorder, and stress-related bodily disorders: the role of the hypothalamus–pituitary–adrenal axis. *Biological psychology* 2001;57(1-3):141-52. doi: https://doi.org/10.1016/s0301-0511(01)00092-8
32. De Kloet ER, Vermetten E, Heijnen CJ, et al. Enhanced cortisol suppression in response to dexamethasone administration in traumatized veterans with and without posttraumatic stress disorder. *Psychoneuroendocrinology* 2007;32(3):215-26. doi: https://doi.org/10.1016/j.psyneuen.2006.12.009
33. Neylan TC, Brunet A, Pole N, et al. PTSD symptoms predict waking salivary cortisol levels in police officers. *Psychoneuroendocrinology* 2005;30(4):373-81. doi: https://doi.org/10.1016/j.psyneuen.2004.10.005
34. Wessa M, Rohleder N, Kirschbaum C, et al. Altered cortisol awakening response in posttraumatic stress disorder. *Psychoneuroendocrinology* 2006;31(2):209-15. doi: https://doi.org/10.1016/j.psyneuen.2005.06.010
35. Yehuda R, Golier JA, Kaufman S. Circadian rhythm of salivary cortisol in Holocaust survivors with and without PTSD. *American Journal of Psychiatry* 2005;162(5):998-1000. doi: https://doi.org/10.1176/appi.ajp.162.5.998
36. Pinto RJ, Correia-Santos P, Costa-Leite J, et al. Cortisol awakening response among women exposed to intimate partner violence. *Psychoneuroendocrinology* 2016;74:57-64. doi: http://dx.doi.org/10.1016/j.psyneuen.2016.08.024
37. Young EA, Breslau N. Cortisol and catecholamines in posttraumatic stress disorder: an epidemiologic community study. *Archives of General Psychiatry* 2004;61(4):394-401. doi: https://doi.org/10.1001/archpsyc.61.4.394
38. Shalev AY, Videlock EJ, Peleg T, et al. Stress hormones and posttraumatic stress disorder in civilian trauma victims: a longitudinal study. Part I: HPA axis responses. *The International Journal of Neuropsychopharmacology* 2008;11(3):365-72. doi: https://doi.org/10.1017/s1461145707008127
39. Meewisse ML, Reitsma JB, De Vries G-J, et al. Cortisol and post-traumatic stress disorder in adults. *British Journal of Psychiatry* 2007;191(5):387-92. doi: https://doi.org/10.1192/bjp.bp.106.024877
40. Habersaat S, Borghini A, Nessi J, et al. Posttraumatic stress symptoms and cortisol regulation in mothers of very preterm infants. *Stress and Health* 2014;30(2):134-41. doi: https://doi.org/10.1002/smi.2503
41. Santa Ana EJ, Saladin ME, Back SE, et al. PTSD and the HPA axis: Differences in response to the cold pressor task among individual with child vs. adult trauma.

For peer review only - http://bmjopen.bmj.com/site/about/guidelines.xhtml

Psychoneuroendocrinology 2006;31(4):501-09. doi: https://doi.org/10.1016/j.psyneuen.2005.11.009

42. Bremner JD, Vythilingam M, Vermetten E, et al. Cortisol response to a cognitive stress challenge in posttraumatic stress disorder (PTSD) related to childhood abuse. *Psychoneuroendocrinology* 2003;28(6):733-50. doi: https://doi.org/10.1016/s0306-4530(02)00067-7
43. Vyas A, Bernal S, Chattarji S. Effects of chronic stress on dendritic arborization in the central and extended amygdala. *Brain Research* 2003;965(1-2):290-94. doi: https://doi.org/10.1016/s0006-8993(02)04162-8
44. Fries E, Hesse J, Hellhammer J, et al. A new view on hypocortisolism. *Psychoneuroendocrinology* 2005;30(10):1010-16. doi: https://doi.org/10.1016/j.psyneuen.2005.04.006
45. Chrousos GP. Stress and disorders of the stress system. *Nature reviews endocrinology* 2009;5(7):374-81. doi: https://doi.org/10.1038/nrendo.2009.106
46. Beauchaine TP, Thayer JF. Heart rate variability as a transdiagnostic biomarker of psychopathology. *International Journal of Psychophysiology* 2015;98(2):338-50. doi: https://doi.org/10.1016/j.ijpsycho.2015.08.004
47. Nagpal M, Gleichauf K, Ginsberg J. Meta-analysis of heart rate variability as a psychophysiological indicator of posttraumatic stress disorder. *Trauma & Treatment* 2013;3(1) doi: https://doi.org/10.4172/2167-1222.1000182
48. Norte CE, Souza GG, Vilete L, et al. They know their trauma by heart: an assessment of psychophysiological failure to recover in PTSD. *Journal of affective disorders* 2013;150(1):136-41. doi: https://doi.org/10.1016/j.jad.2012.11.039
49. Maher MJ, Rego SA, Asnis GM. Sleep disturbances in patients with post-traumatic stress disorder. *CNS Drugs* 2006;20(7):567-90. doi: https://doi.org/10.2165/00023210-200620070-00003
50. Porcheret K, Holmes EA, Goodwin GM, et al. Psychological impact of an analogue traumatic event reduced by sleep deprivation. *Sleep* 2015;38(7):1017-25. doi: http://dx.doi.org/10.5665/sleep.4802
51. Kleim B, Wysokowsky J, Schmid N, et al. Effects of sleep after experimental trauma on intrusive emotional memories. *Sleep* 2016;39(12):2125-32. doi: https://doi.org/10.5665/sleep.6310
52. Woud ML, Cwik JC, Blackwell SE, et al. Does napping enhance the effects of Cognitive Bias Modification-Appraisal training? An experimental study. *PloS one* 2018;13(2):e0192837. doi: https://doi.org/10.1371/journal.pone.0192837
53. Porcheret K, van Heugten–van der Kloet D, Goodwin GM, et al. Investigation of the impact of total sleep deprivation at home on the number of intrusive memories to an analogue trauma. *Translational psychiatry* 2019;9(1) doi: https://doi.org/10.1038/s41398-019-0403-z
54. Feldman R. Parent-Infant synchrony and the construction of shared timing: psychological precursors, developmental outcomes, and risk conditions *Journal of Child Psychology and Psychiatry* 2007;48(3/4):329-54. doi: https://doi.org/10.1111/j.1469-7610.2006.01701.x
55. Treyvaud K, Anderson VA, Lee KJ, et al. Parental mental health and early social-emotional development of children born very preterm. *Journal of pediatric psychology* 2010;35(7):768-77. doi: https://doi.org/10.1093/jpepsy/jsp109

For peer review only - http://bmjopen.bmj.com/site/about/guidelines.xhtml

56. Blandon AY, Calkins SD, Keane SP, et al. Individual differences in trajectories of emotion regulation processes: the effects of maternal depressive symptomatology and children's physiological regulation. *Developmental Psychology* 2008;44(4):1110-23. doi: https://doi.org/10.1037/0012-1649.44.4.1110
57. Eisenberg N, Valiente C, Morris AS, et al. Longitudinal relations among parental emotional expressivity, children's regulation, and quality of socio-emotional functioning *Developmental Psychology* 2003;39(1):3-19. doi: https://doi.org/10.1037/0012-1649.39.1.3
58. Feng X, Shaw DS, Kovacs M, et al. Emotion regulation in preschoolers: the roles of behavioral inhibition, maternal affective behavior, and maternal depression. *Journal of Child Psychology and Psychiatry* 2008;49(2):132-41. doi: https://doi.org/10.1111/j.1469-7610.2007.01828.x
59. Francis DD, Meaney MJ. Maternal care and the development of stress responses. *Current Opinion in Neurobiology* 1999;9(1):128-34. doi: https://doi.org/10.1016/s0959-4388(99)80016-6
60. Liu D, Diorio J, Tannenbaum B, et al. Maternal care, hippocampal glucocorticoid receptors, and hypothalamic-pituitary-adrenal responses to stress. *Science* 1997;277(5332):1659-62. doi: https://doi.org/10.1126/science.277.5332.1659
61. Gunzenhauser N. Infant stimulation: for whom, what kind, when, and how much? Skillman, NJ: Johnson and Johnson 1987.
62. Schore AN. Affect regulation and the origin of the self. Hillsdale, NJ: Lawrence Erlbaum Associates, Inc 1994.
63. Liu K, Ruggero CJ, Goldstein B, et al. Elevated cortisol in healthy female adolescent offspring of mothers with posttraumatic stress disorder. *Journal of Anxiety Disorders* 2016;40:37-43. doi: http://dx.doi.org/10.1016/j.janxdis.2016.04.003
64. Essex MJ, Klein MH, Cho E, et al. Maternal stress begining in infancy may sensitize children to later stress exposure : Effects on cortisol and behavior. *Biological Psychiatry* 2002;52(8):776 - 84. doi: https://doi.org/10.1016/s0006-3223(02)01553-6
65. Lupien SJ, King S, Meaney MJ, et al. Child's stress hormone levels correlate with mother's socio-economic status and depressive state. *Biological Psychiatry* 2000;48(10):976 - 80. doi: https://doi.org/10.1016/s0006-3223(00)00965-3
66. Warren SL, Gunnar MR, Kagan J, et al. Maternal panic disorder: infant temperament, neurophysiology and parentiong behaviors. *Journal of the American Academy of Child and Adolescent Psychiatry* 2003;42(7):814 - 25 doi: https://doi.org/10.1097/01.chi.0000046872.56865.02
67. Yehuda R, Halligan SL, Bierer LM. Cortisol levels in adult offspring of Holocaust survivors: relation to PTSD symptom severity in the parent and child. *Psychoneuroendocrinology* 2002;27(1-2):171-80. doi: https://doi.org/10.1016/s0306-4530(01)00043-9
68. Ehlert U. Understanding the trans-generational consequences of prenatal stress. *Journal of psychosomatic research* 2013;75(4):297-98. doi: https://doi.org/10.1016/j.jpsychores.2013.09.002
69. Muller-Nix C, Forcada-Guex M, Pierrehumbert B, et al. Prematurity, maternal stress and mother-child interactions. *Early Human Development* 2004;79(2):145-58. doi: https://doi.org/10.1016/j.earlhumdev.2004.05.002

For peer review only - http://bmjopen.bmj.com/site/about/guidelines.xhtml

70. Schmücker G, Brisch K-H, Köhntop B, et al. The influence of prematurity, maternal anxiety, and infants' neurobiological risk on mother - infant interactions. *Infant Mental Health Journal* 2005;26(5):423 - 41. doi: https://doi.org/10.1002/imhj.20066
71. Zelkowitz P, Papageorgiou A. Maternal anxiety: an emerging prognostic factor in neonatology. *Acta Paediatrica* 2005;94(12):1704 - 05 doi: https://doi.org/10.1080/08035250500355030
72. Korja R, Savonlahti E, Ahlqvist-Bjorkroth S, et al. Maternal depression is associated with mother-infant interaction in preterm infants. *Acta paediatrica* 2008;97(6):724-30. doi: https://doi.org/10.1111/j.1651-2227.2008.00733.x
73. Crockett EE, Holmes BM, Granger DA, et al. Maternal Disrupted Communication During Face-to-Face Interaction at 4 months: Relation to Maternal and Infant Cortisol Among at-Risk Families. *Infancy* 2013;18(6):1111-34. doi: https://doi.org/10.1111/infa.12015
74. Ahnert L, Gunnar MR, Lamb ME, et al. Transition to Child Care: Associations with Infant–Mother Attachment, Infant Negative Emotion, and Cortisol Elevations. *Child Development* 2004;75(3):639-50. doi: https://doi.org/10.1111/j.1467-8624.2004.00698.x
75. Domes G, Heinrichs M, Reichwald U, et al. Hypothalamic-pituitary-adrenal axis reactivity to psychological stress and memory in middle-aged women: high responders exhibit enhanced declarative memory performance. *Psychoneuroendocrinology* 2002;27(7):843 - 53. doi: https://doi.org/10.1016/s0306-4530(01)00085-3
76. Nachmias M, Gunnar M, Mangelsdorf S, et al. Behavioral inhibition and stress reactivity: the moderating role of attachment security. *Child development* 1996;67(2):508-22. doi: https://doi.org/10.1111/j.1467-8624.1996.tb01748.x
77. Spangler G, Scheubeck R. Behavioral organization in newborns and its relation to adrenocortical and cardiac activity. *Child Development* 1993;64:622-33.
78. Cordero MI, Moser DA, Manini A, et al. Effects of interpersonal violence-related post-traumatic stress disorder (PTSD) on mother and child diurnal cortisol rhythm and cortisol reactivity to a laboratory stressor involving separation. *Hormones and behavior* 2017;90:15-24. doi: https://doi.org/10.1016/j.yhbeh.2017.02.007
79. Dierckx B, Tulen JH, van den Berg MP, et al. Maternal psychopathology influences infant heart rate variability: Generation R Study. *Psychosomatic Medicine* 2009;71(3):313-21. doi: https://doi.org/10.1097/psy.0b013e318198a82c
80. Braeken MA, Kemp AH, Outhred T, et al. Pregnant mothers with resolved anxiety disorders and their offspring have reduced heart rate variability: implications for the health of children. *PloS one* 2013;8(12):e83186. doi: https://doi.org/10.1371/journal.pone.0083186
81. Jovanovic T, Smith A, Kamkwalala A, et al. Physiological markers of anxiety are increased in children of abused mothers. *Journal of Child Psychology and Psychiatry* 2011;52(8):844-52. doi: https://doi.org/10.1111/j.1469-7610.2011.02410.x
82. Bastos MH, Furuta M, Small R, et al. Debriefing interventions for the prevention of psychological trauma in women following childbirth. *Cochrane Database of Systematic Reviews* 2015 doi: https://doi.org/10.1002/14651858.cd007194.pub2
83. O'Donnell ML, Elliott P, Lau W, et al. PTSD symptom trajectories: From early to chronic response. *Behaviour research and therapy* 2007;45(3):601-06. doi: https://doi.org/10.1016/j.brat.2006.03.015

For peer review only - http://bmjopen.bmj.com/site/about/guidelines.xhtml

84. Brewin CR. Episodic memory, perceptual memory, and their interaction: foundations for a theory of posttraumatic stress disorder. *Psychol Bulletin* 2014;140(1):69-97. doi: https://doi.org/10.1037/a0033722
85. Holmes EA, James EL, Coode-Bate T, et al. Can playing the computer game "Tetris" reduce the build-up of flashbacks for trauma? A proposal from cognitive science. *PLoS ONE v* 2009;4(1):e4153. doi: https://doi.org/10.1371/journal.pone.0004153
86. Holmes EA, James EL, Kilford EJ, et al. Key steps in developing a cognitive vaccine against traumatic flashbacks: visuospatial Tetris versus verbal Pub Quiz. *PloS one* 2010;5(11):e13706. doi: https://doi.org/10.1371/journal.pone.0013706
87. Kindt M, van den Hout M, Arntz A, et al. The influence of data-driven versus conceptually-driven processing on the development of PTSD-like symptoms. *Journal of behavior therapy and experimental psychiatry* 2008;39(4):546-57. doi: https://doi.org/10.1016/j.jbtep.2007.12.003
88. Deeprose C, Zhang S, Dejong H, et al. Imagery in the aftermath of viewing a traumatic film: Using cognitive tasks to modulate the development of involuntary memory. *Journal of behavior therapy and experimental psychiatry* 2012;43(2):758-64. doi: https://doi.org/10.1016/j.jbtep.2011.10.008
89. Walker MP, Brakefield T, Hobson JA, et al. Dissociable stages of human memory consolidation and reconsolidation. *Nature* 2003;425(6958):616-20. doi: https://doi.org/10.1038/nature01930
90. McGaugh JL. Memory--a Century of Consolidation. *Science* 2000;287(5451):248-51. doi: 10.1126/science.287.5451.248
91. Nader K, Schafe GE, LeDoux JE. The labile nature of consolidation theory. *Nature Reviews Neuroscience* 2000;1(3):216-19. doi: https://doi.org/10.1038/35044580
92. Iyadurai L, Visser RM, Lau-Zhu A, et al. Intrusive memories of trauma: A target for research bridging cognitive science and its clinical application. *Clinical psychology review* 2018;69:67-82. doi: https://doi.org/10.1016/j.cpr.2018.08.005
93. Iyadurai L, Blackwell SE, Meiser-Stedman R, et al. Preventing intrusive memories after trauma via a brief intervention involving Tetris computer game play in the emergency department: a proof-of-concept randomized controlled trial. *Molecular psychiatry* 2018;23(3):674-82. doi: https://doi.org/10.1038/mp.2017.23
94. Foa EB, Cashman L, Jaycox L, et al. The validation of a self-report measure of posttraumatic stress disorder: the Posttraumatic Diagnostic Scale. *Psychological assessment* 1997;9(4):445-51. doi: https://doi.org/10.1037/1040-3590.9.4.445
95. Kim J, Shin W. How to Do Random Allocation (Randomization). *Clinics in Orthopedic Surgery* 2014;6(1):103-09. doi: https://doi.org/10.4055/cios.2014.6.1.103
96. Furuta M, Horsch A, Ng ES, et al. Effectiveness of trauma-focused psychological therapies for treating post-traumatic stress disorder symptoms in women following childbirth: a systematic review and meta-analysis. *Frontiers in psychiatry* 2018;9 doi: https://doi.org/10.3389/fpsyt.2018.00591
97. Roberts NP, Kitchiner NJ, Kenardy J, et al. Multiple session early psychological interventions for the prevention of post-traumatic stress disorder. *Cochrane Database of Systematic Reviews* 2009 doi: https://doi.org/10.1002/14651858.cd006869.pub2
98. Sijbrandij M, Kleiboer A, Bisson JI, et al. Pharmacological prevention of post-traumatic stress disorder and acute stress disorder: a systematic review and meta-analysis. *The*

For peer review only - http://bmjopen.bmj.com/site/about/guidelines.xhtml

Lancet Psychiatry 2015;2(5):413-21. doi: https://doi.org/10.1016/s2215-0366(14)00121-7
99. Rose SC, Bisson J, Churchill R, et al. Psychological debriefing for preventing post traumatic stress disorder (PTSD). *Cochrane Database of Systematic Reviews* 2002 doi: https://doi.org/10.1002/14651858.cd000560
100. Weathers FW, Bovin MJ, Lee DJ, et al. The Clinician-Administered PTSD Scale for DSM–5 (CAPS-5): Development and Initial Psychometric Evaluation in Military Veterans. *Psychological Assessment* 2018;30(3):383-95. doi: https://doi.org/10.1037/pas0000486
101. Blevins CA, Weathers FW, Davis MT, et al. The posttraumatic stress disorder checklist for DSM-5 (PCL-5): Development and initial psychometric evaluation. *Journal of Traumatic Stress* 2015;28(6):489-98. doi: https://doi.org/10.1002/jts.22059
102. Wild D, Grove A, Martin M, et al. Principles of Good Practice for the Translation and Cultural Adaptation Process for Patient-Reported Outcomes (PRO) Measures: report of the ISPOR Task Force for Translation and Cultural Adaptation. *Value Health* 2005;8(2):94-104. doi: https://doi.org/10.1111/j.1524-4733.2005.04054.x
103. Bovin MJ, Marx BP, Weathers FW, et al. Psychometric properties of the PTSD checklist for diagnostic and statistical manual of mental disorders–fifth edition (PCL-5) in veterans. *Psychological Assessment* 2016;28(11):1379-91. doi: https://doi.org/10.1037/pas0000254
104. Ashbaugh AR, Houle-Johnson S, Herbert C, et al. Psychometric Validation of the English and French Versions of the Posttraumatic Stress Disorder Checklist for DSM-5 (PCL-5). *PloS one* 2016;11(10):e0161645. doi: https://doi.org/10.1371/journal.pone.0161645
105. Bryant RA, Moulds ML, Guthrie RM. Acute Stress Disorder Scale: a self-report measure of acute stress disorder. *Psychological Assessment* 2000;12(1):61-68. doi: https://doi.org/10.1037//1040-3590.12.1.61
106. American Psychiatric Association. Diagnostic and statistical manual of mental disorders. 4th ed., text rev. ed. Washington, DC: American Psychiatric Publishing 2000.
107. Zigmond AS, Snaith RP. The hospital anxiety and depression scale. *Acta psychiatrica scandinavica* 1983;67(6):361-70. doi: https://doi.org/10.1111/j.1600-0447.1983.tb09716.x
108. Bocerean C, Dupret E. A validation study of the Hospital Anxiety and Depression Scale (HADS) in a large sample of French employees. *BMC Psychiatry* 2014;14(1) doi: https://doi.org/10.1186/s12888-014-0354-0 [published Online First: 2014/12/17]
109. Cox JL, Holden JM, Sagovsky R. Detection of postnatal depression. Development of the 10-item Edinburgh Postnatal Depression Scale. *British Journal of Psychiatry* 1987;150(6):782-86. doi: https://doi.org/10.1192/bjp.150.6.782
110. Guedeney N, Fermanian J. Validation study of the French version of the Edinburgh Postnatal Depression Scale (EPDS): new results about use and psychometric properties. *Eur Psychiatry* 1998;13(2):83-89. doi: https://doi.org/10.1016/s0924-9338(98)80023-0
111. Esliger DW, Rowlands AV, Hurst TL, et al. Validation of the GENEA Accelerometer. *Medicine & Science in Sports & Exercise* 2011;43(6):1085-93. doi: 10.1249/MSS.0b013e31820513be
112. Buysse DJ, Reynolds CF, Monk TH, et al. The Pittsburgh sleep quality index: A new instrument for psychiatric practice and research. *Psychiatry research* 1989;28(2):193-213. doi: https://doi.org/10.1016/0165-1781(89)90047-4

For peer review only - http://bmjopen.bmj.com/site/about/guidelines.xhtml

113. Blais F, Gendron L, Mimeault V, et al. Evaluation de l'insomnie: Validation de trois questionnaires. *L'Encéphale: Revue de psychiatrie clinique biologique et thérapeutique* 1997;23(6):447-53.
114. Ait-Aoudia M, Levy PP, Bui E, et al. Validation of the French version of the Pittsburgh Sleep Quality Index Addendum for posttraumatic stress disorder. *European journal of psychotraumatology* 2013;4(1):19298. doi: https://doi.org/10.3402/ejpt.v4i0.19298
115. Kirschbaum C, Pirke K-M, Hellhammer DH. The 'Trier Social Stress Test'–a tool for investigating psychobiological stress responses in a laboratory setting. *Neuropsychobiology* 1993;28(1-2):76-81. doi: https://doi.org/10.1159/000119004
116. Brazelton TB, Nugent JK. The Neontal Behavioral Assessment Scale. 4th ed. London: Mac Keith Press 2011.
117. Tronick E, Als H, Adamson L, et al. The infant's response to entrapment between contradictory messages in face-to-face interaction. *Journal of the American Academy of Child Psychiatry* 1978;17(1):1-13. doi: https://doi.org/10.1016/s0002-7138(09)62273-1
118. Provenzi L, Giusti L, Montirosso R. Do infants exhibit significant cortisol reactivity to the Face-to-Face Still-Face paradigm? A narrative review and meta-analysis. *Developmental Review* 2016;42:34-55. doi: https://doi.org/10.1016/j.dr.2016.07.001
119. Haley DW, Stansbury K. Infant stress and parent responsiveness: regulation of physiology and behavior during still-face and reunion. *Child development* 2003;74(5):1534-46. doi: https://doi.org/10.1111/1467-8624.00621
120. DiCorcia JA, Snidman N, Sravish AV, et al. Evaluating the Nature of the Still-Face Effect in the Double Face-to-Face Still-Face Paradigm Using Different Comparison Groups. *Infancy* 2016;21(3):332-52. doi: https://doi.org/10.1111/infa.12123
121. Taylor A, Atkins R, Kumar R, et al. A new Mother-to-Infant Bonding Scale: links with early maternal mood. *Archives of women's mental health* 2005;8(1):45-51. doi: https://doi.org/10.1007/s00737-005-0074-z
122. Bienfait M, Haquet A, Maury M, et al. Traduction française de l'autoquestionnaire MIBS (Mother to Infant Bonding Scale) et validation comme évaluation du lien mère-nouveau-né en maternité. *Devenir* 2017;29(4):233-53. doi: https://doi.org/10.3917/dev.174.0233
123. Biringen Z. The Emotional Availability (EA) Scales and EA Zones Evaluation: Infancy/early childhood version; middle childhood/youth versions; therapist/interventionist/professional manual; couple relationship manual. 4 ed. Colorado: Boulder 2008.
124. Biringen Z, Derscheid D, Vliegen N, et al. Emotional Availability (EA): Theoretical background, empirical research using the EA Scales and clinical implications. . *Developmental Review* 2014;34:114-67. doi: https://doi.org/10.1016/j.dr.2014.01.002
125. Putnam SP, Helbig AL, Gartstein MA, et al. Development and Assessment of Short and Very Short Forms of the Infant Behavior Questionnaire–Revised. *Journal of Personality Assessment* 2014;96(4):445-58. doi: https://doi.org/10.1080/00223891.2013.841171
126. Bayley N, Reuner G. Bayley scales of infant and toddler development: Bayley-III: Harcourt Assessment, Psych. Corporation San Antonio, Tex, USA 2006.
127. Albers EM, Marianne Riksen-Walraven J, Sweep FC, et al. Maternal behavior predicts infant cortisol recovery from a mild everyday stressor. *Journal of Child Psychology*

For peer review only - http://bmjopen.bmj.com/site/about/guidelines.xhtml

and Psychiatry 2008;49(1):97-103. doi: https://doi.org/10.1111/j.1469-7610.2007.01818.x
128. Largo RH, Pfister D, Molinari L, et al. Significance of prenatal, perinatal and postnatal factors in the development of AGA preterm infants at five to seven years. *Developmental Medicine & Child Neurology* 1989;31(4):440-56. doi: https://doi.org/10.1111/j.1469-8749.1989.tb04022.x
129. Parry G, Tucker J, Tarnow-Mordi W, et al. CRIB II: an update of the clinical risk index for babies score. *Lancet* 2003;361(9371):1789-91. doi: https://doi.org/10.1016/s0140-6736(03)13397-1
130. Harris PA, Taylor R, Minor BL, et al. The REDCap consortium: Building an international community of software platform partners. *Journal of Biomedical Informatics* 2019;95:103208. doi: https://doi.org/10.1016/j.jbi.2019.103208
131. Harris PA, Taylor R, Thielke R, et al. Research electronic data capture (REDCap)—a metadata-driven methodology and workflow process for providing translational research informatics support. *Journal of Biomedical Informatics* 2009;42(2):377-81. doi: https://doi.org/10.1016/j.jbi.2008.08.010
132. Camm AJ, Malik M, Bigger J, et al. Heart rate variability: standards of measurement, physiological interpretation and clinical use. . *European Heart Journal* 1996;17(3):354-81. doi: https://doi.org/10.1093/oxfordjournals.eurheartj.a014868
133. Tarvainen MP, Niskanen JP, Lipponen JA, et al. Kubios HRV--heart rate variability analysis software. *Computer methods and programs in biomedicine* 2014;113(1):210-20. doi: https://doi.org/10.1016/j.cmpb.2013.07.024 [published Online First: 2013/09/24]
134. Razurel C, Kaiser B, Dupuis M, et al. Validation of the post-delivery perceived stress inventory. *Psychology, Health & Medicine* 2014;19(1):70-82. doi: https://doi.org/10.1080/13548506.2013.774431
135. Koch FS, Sepa A, Ludvigsson J. Psychological stress and obesity. *The Journal of Pediatrics* 2008;153(6):839-44. doi: https://doi.org/10.1016/j.jpeds.2008.06.016
136. Obel C, Hedegaard M, Henriksen TB, et al. Stress and salivary cortisol during pregnancy. *Psychoneuroendocrinology* 2005;30(7):647-56. doi: https://doi.org/10.1016/j.psyneuen.2004.11.006
137. Razurel C, Kaiser B, Dupuis M, et al. Validation of the Postnatal Perceived Stress Inventory in a French Speaking Population of Primiparous Women. *Journal of Obstetric, Gynecologic, & Neonatal Nursing* 2013;42(6):685-96. doi: https://doi.org/10.1111/1552-6909.12251
138. Abidin RR. Parenting stress index-short form: Pediatric Psychology Press Charlottesville, VA 1990.
139. Haskett ME, Ahern LS, Ward CS, et al. Factor structure and validity of the parenting stress index-short form. *Journal of Clinical Child & Adolescent Psychology* 2006;35(2):302-12. doi: https://doi.org/10.1207/s15374424jccp3502_14
140. Moser A, Stuck AE, Silliman RA, et al. The eight-item modified Medical Outcomes Study Social Support Survey: psychometric evaluation showed excellent performance. *Journal of Clinical Epidemiology* 2012;65(10):1107-16. doi: http://dx.doi.org/10.1016/j.jclinepi.2012.04.007
141. Busby DM, Christensen C, Crane DR, et al. A revision of the Dyadic Adjustment Scale for use with distressed and nondistressed couples: Construct hierarchy and

For peer review only - http://bmjopen.bmj.com/site/about/guidelines.xhtml

multidimensional scales. *Journal of Marital and family Therapy* 1995;21(3):289-308. doi: https://doi.org/10.1111/j.1752-0606.1995.tb00163.x
142. Crane DR, Middleton KC, Bean RA. Establishing criterion scores for the Kansas marital satisfaction scale and the revised dyadic adjustment scale. *American Journal of Family Therapy* 2000;28(1):53-60. doi: https://doi.org/10.1080/019261800261815
143. Horne JA, Ostberg O. A self-assessment questionnaire to determine morningness-eveningness in human circadian rhythms. *International Journal of Chronobiology* 1976;4:97-110.
144. Esliger DW, Rowlands AV, Hurst TL, et al. Validation of the GENEA Accelerometer. 2011
145. Blake DD, Weathers FW, Nagy LM, et al. The development of a clinician-administered PTSD scale. *Journal of Traumatic Stress* 1995;8(1):75-90. doi: https://doi.org/10.1007/bf02105408
146. Dubowitz L, Mercuri E, Dubowitz V. An optimality score for the neurologic examination of the term newborn. *The Journal of pediatrics* 1998;133(3):406-16. doi: https://doi.org/10.1016/s0022-3476(98)70279-3

For peer review only

For peer review only - http://bmjopen.bmj.com/site/about/guidelines.xhtml

Figure legends

Figure 1. Study variables: Processes in mothers, partner, infants, and their interactions following traumatic childbirth

Figure 2. Flowchart of study procedures

For peer review only

For peer review only - http://bmjopen.bmj.com/site/about/guidelines.xhtml

Table 1. Overview of primary, secondary and other outcomes, measures, and time points

	DOMAIN	VARIABLES	INSTRUMENTS	TIMING			
				6h after ECS (T1)	≤ 1 week (T2)	6 weeks (T3)	6 months (T4)
**MOTHER**	Sociodemographic and medical data	Sociodemographic variables	Demographic questionnaire		X	X	X
		Medical data	Obstetric data and pregnancy outcomes from medical records		X		X
			Menstrual cycle (if applicable)				X
			Breastfeeding diary/questions		X	X	X
	Acceptability and expectancy of the intervention	Satisfaction and expectancy	Feedback questionnaire	X			
	Maternal psychological vulnerability	Intrusive trauma-related memories	Trauma-related intrusive memories diary [4]		X		
		Stress exposure and perception	Post-delivery Perceived Stress Inventory (PDPSI) [134]		X		
			Major life events [135 136]		X		X
			Postnatal Perceived Stress Inventory (PPSI) [137]				X
			Parenting Stress Index – Short Form (PSI-SF) [138 139]				X
		PTSD	Clinician-administered PTSD scale (CAPS) [102]			X	X
			PTSD Checklist (PCL-5) [101]			X	X
		ASD	Acute Stress Disorder Scale (ASDS) [105]	X	X		
		Anxiety	Hospital Anxiety and Depression Scale: anxiety subscale (HADS) [107]	X	X	X	X

For peer review only

		Depression	Edinburgh Postnatal Depression Scale (EPDS) [109 109]	X	X	X	X
		Social support	Modified Medical Outcomes Study Social Support Survey (MOS-8) [140]		X	X	X
			Revised Dyadic Adjustment Scale (RDAS) [141 142]		X	X	X
		Sleep	Pittsburgh Sleep Quality Index Addendum for posttraumatic stress disorder (PSQI-A) [114]			X	X
			Morningness-Eveningness questionnaire (MEQ) [143]		X		
			Pittsburgh Sleep Quality Index (PSQI) [112]		X	X	X
			Sleep diary		X		
			Overnight accelerometer assessments (GENEActiv®)[144]		X		X
	Maternal physiological stress responses	Physiological regulation	Baseline cortisol and cortisol daily profile (saliva)		X		X
			Resting heart rate and heart rate variability (Firstbeat Bodyguard 2)				X
		Physiological stress reactivity	Cortisol (saliva)				X
			Heart rate variability (Firstbeat Bodyguard 2)				X
**PARTNER**	Sociodemographic data	Sociodemographic variables	Demographic questionnaire		X	X	X
	Paternal psychological vulnerability	Intrusive traumatic memories	Traumatic intrusions diary [4]		X		
		Stress exposure and	Major life events [135 136]		X		X

For peer review only - http://bmjopen.bmj.com/site/about/guidelines.xhtml

		perception	Parenting Stress Index – Short Form (PSI-SF) [138 139]				X
		PTSD	Clinician-administered PTSD scale (CAPS) [102 145]			X	X
			PTSD Checklist (PCL-5) [101]			X	X
		ASD	Acute Stress Disorder Scale (ASDS) [105]		X		
		Anxiety	Hospital Anxiety and Depression Scale: anxiety subscale (HADS) [107]		X	X	X
		Depression	Edinburgh Postnatal Depression Scale (EPDS) [109]		X	X	X
		Social support	Modified Medical Outcomes Study Social Support Survey (MOS-8) [140]		X	X	X
			Revised Dyadic Adjustment Scale (RDAS) [142 141]		X	X	X
		Perception of ECS-related trauma	Screening questions of the Posttraumatic Diagnostic Scale [94]		X		
		Sleep	Pittsburgh Sleep Quality Index (PSQI) [112]		X	X	X
			Pittsburgh Sleep Quality Index Addendum for posttraumatic stress disorder (PSQI-A)[114]			X	X
			Morningness-Eveningness questionnaire (MEQ) [143]		X		
	Partner physiological responses	Physiological regulation	Cortisol daily profile (saliva)		X		X

only

**INFANT**	Sociodemographic and medical data	Sociodemographic variables	Demographic questionnaire (completed by mother)		**X**	**X**	**X**
		Medical data	Neonatal outcomes from medical records		**X**		
	Infant neurodevelopmental vulnerability	Infant irritability	Dubowitz neurologic examination [146]		**X**		
	Infant physiological stress responses	Physiological regulation	Baseline cortisol and cortisol daily profile (saliva)				**X**
			Resting heart rate and heart rate variability by (Firstbeat Bodyguard 2)		**X**		**X**
		Physiological stress reactivity	Cortisol (saliva)		**X**		**X**
			Heart rate variability (Firstbeat Bodyguard 2)		**X**		**X**
	Developmental outcomes	Neonatal behaviour	Neonatal Behavioural Assessment Scale (NBAS) [116]		**X**		
		Infant development	Infant Behaviour Questionnaire-revised (IBQ-R) [125]				**X***
			Bayley Scales of Infant Development (Bayley-III; clinician-rated) [126]				**X**
**PARENT-INFANT INTERACTION**	Mother-infant interaction	Maternal sensitivity	Emotional Availability Scale (EAS, clinician-rated) [123, 124]				**X**
		Bonding	Mother-to-Infant-Bonding Scale (MIBS) [121]		**X**	**X**	**X**
	Partner-infant interaction	Bonding	Mother-Infant-Bonding Scale (MIBS) [121]		**X**	**X**	**X**

Note: * = completed by mothers and partners

For peer review only

Table 2. Other Outcomes and a brief description of the measures used for each outcome. Participant groups for whom the outcome is relevant are highlighted in bold.

Domain	Instruments	Description
**Maternal and paternal psychological vulnerability**	Morningness-Eveningness Questionnaire (MEQ) [143] (at ≤1 week follow-up)	19 multiple-choice questions self-assessment questionnaire investigating the **mother and partner**'s circadian rhythm. Scores are summed to create a total and a higher score indicates a more 'morning' person.
	Modified Medical Outcomes Study Social Support Survey (MOS-8) [140] [122] (≤1 and 6 weeks, and 6 months follow-up)	8-item self-report questionnaire assessing **mother and partner** social support across four functional support scales: emotional/ informative, tangible, affectionate, and positive social interaction. Items are answered on a 6 point scale (0= *Never*, 5 = *Always*).
	Revised Dyadic Adjustment Scale (RDAS) [141] [142] (at ≤1 and 6 weeks, and 6 months follow-up)	14-item self-report questionnaire measuring the **mother and the partner** relationship satisfaction. Three categories are evaluated: consensus, satisfaction and cohesion.
	Parenting Stress Index - Short Form (PSI-SF)[139] [138] (6 months follow-up)	Self-report questionnaire measuring the **mother and partner** parenting stress. It consists of three subscales: parental distress, parent-child dysfunctional interactions, and child difficulties. 36 items are answered on a 1-point Likert scale *(1 = Strongly agree, 5 = Strongly disagree).*
	The Post-delivery Perceived Stress Inventory (PDPSI) [134] (at ≤1 week follow-up)	16-item self-report questionnaire assessing **mother's** perceived stress linked to delivery. Each item is a potential stressor the mother may have

	experienced during or after delivery. Mothers are asked whether they found the items more or less stressful using a 5-point Likert scale *(1=Never, 5=Very often).*
Postnatal Perceived Stress Inventory (PPSI)[137] (at 6 weeks, and 6 months follow-up)	19-item self-report questionnaire assessing **maternal** postpartum perceived stress. Each item relates to a potential stressor they may encounter within the postnatal period. Mothers are asked to indicate whether they were more or less stressed on a 5 point scale (1 = Not at all, 5 = Extremely).
Life Events Questionnaire [135 136] (at ≤1 week and 6 months follow-up)	15-item self-report scale where **mother and partners** are asked to indicate if they have experienced different major life events and how stressful they found these events. One item allows participants to identify an event not mentioned and one asks if a major life event occurred within the first trimester of pregnancy.
Trauma-related intrusive memories diary [4] (at ≤1 week follow-up)	Completed by **partners**; see Measures section for more details.
Clinician-administered PTSD scale (CAPS) [100] (at 6 weeks and 6 months follow-up)	Completed by **partners**; see Measures section for more details.
PTSD Checklist (PCL-5) [101] (at 6 weeks and 6 months follow-up)	Completed by **partners**; see Measures section for more details.
Acute Stress Disorder Scale (ASDS) [105] (at ≤1 week follow-up)	Completed by **partners**; see Measures section for more details.
Edinburgh Postnatal Depression Scale (EPDS) [109] (at ≤1 and 6 weeks, and 6 months follow-up)	Completed by **partners**; see Measures section for more details.

For peer review only - http://bmjopen.bmj.com/site/about/guidelines.xhtml

	Anxiety subscale of Hospital Anxiety and Depression Scale (HADS) [107] (at ≤1 and 6 weeks, and 6 months follow-up)	Completed by **partners**; see Measures section for more details.
	Pittsburgh Sleep Quality Index (PSQI) [112] (at ≤1 and 6 weeks, and 6 months follow-up)	Completed by **partners; s**ee Measures section for more details.
	Pittsburgh Sleep Quality Index Addendum for posttraumatic stress disorder (PSQI-A) [114] (at 6 weeks and 6 months follow-up)	Completed by **partners**; see Measures section for more details.
	Perception of ECS-related trauma (at ≤1 week follow-up)	**Partners** complete the 4 screening questions of the Posttraumatic Diagnostic Scale [94]
**Partner physiological regulation**	Cortisol daily profile (at ≤1 and 6 months follow-up)	Completed by **partners**; see Measures section for more details.
**Partner-infant interaction**	Mother-to-Infant-Bonding Scale (MIBS) [121] (at ≤1 and 6 weeks, and 6 months follow-up)	Adapted for partners, **s**ee Measures section for more details
**Demographic Information**	Partner demographics (at ≤1 and 6 weeks, and 6 months follow-up)	Adapted for partners, **s**ee Measures section for more details
	Infant demographics (at ≤1 and 6 weeks, and 6 months follow-up)	**Mothers** report on their child's weight and height.
**Medical data**	Breastfeeding diary and questions (at ≤1 and 6 weeks, and 6 months follow-up)	**Mothers** report on breastfeeding in a 2-day daily diary including breastfeeding length and type (exclusive or mixed).
**Acceptability and expectancy of the intervention**	Self-report questionnaire of satisfaction and acceptability of the intervention (at ≥6 hours following ECS)	On completing the intervention activity, **mothers** complete 7 items to assess intervention fidelity, satisfaction and acceptability of the intervention.
**Infant neurodevelopmental vulnerability**	Dubowitz neurologic examination [146] (at ≤1 week follow-up)	Examination of the **infant** on 34 items subdivided into 6 categories (tone, tone patterns, reflexes, movements, abnormal signs, and behavior). Full examination by a pediatrician follows

For peer review only - http://bmjopen.bmj.com/site/about/guidelines.xhtml

		standardised instructions and takes 10-15 minutes.

For peer review only

For peer review only - http://bmjopen.bmj.com/site/about/guidelines.xhtml

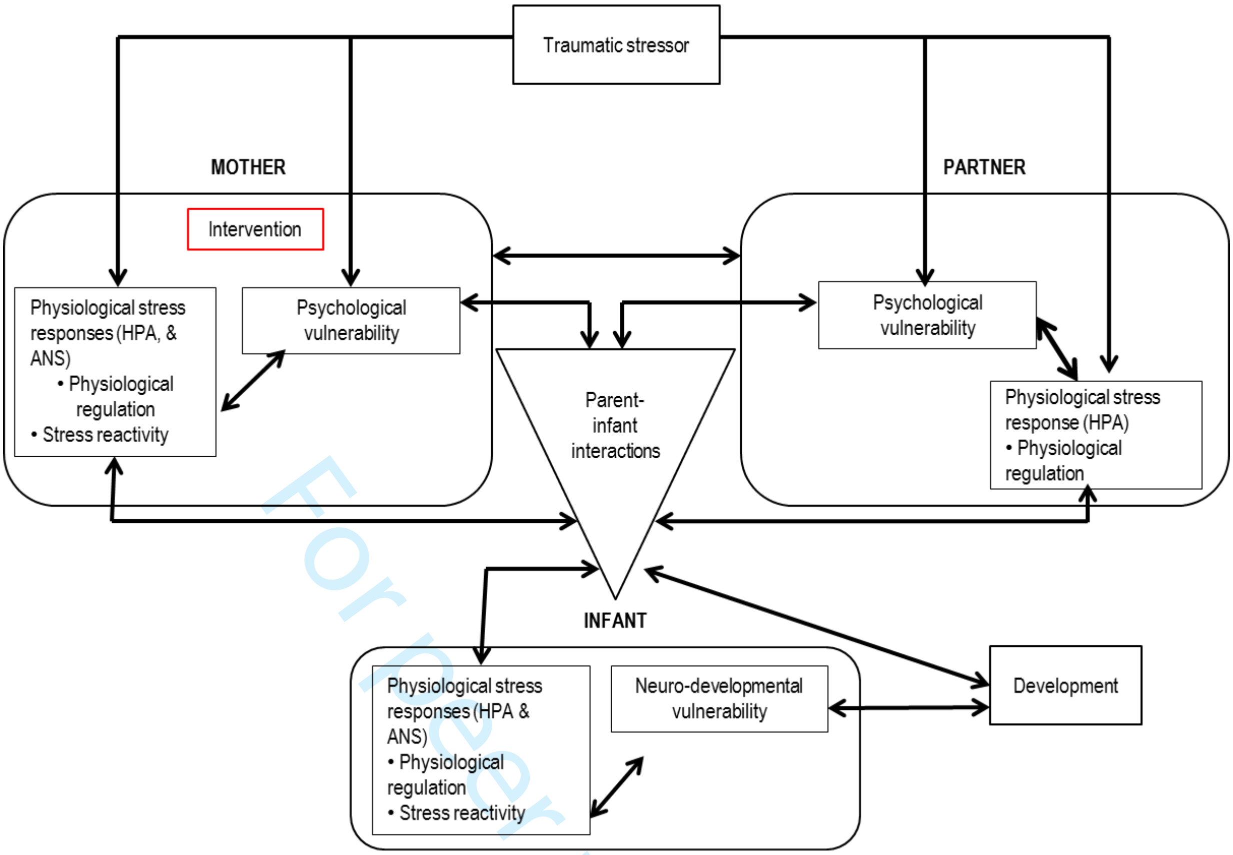

For peer review only

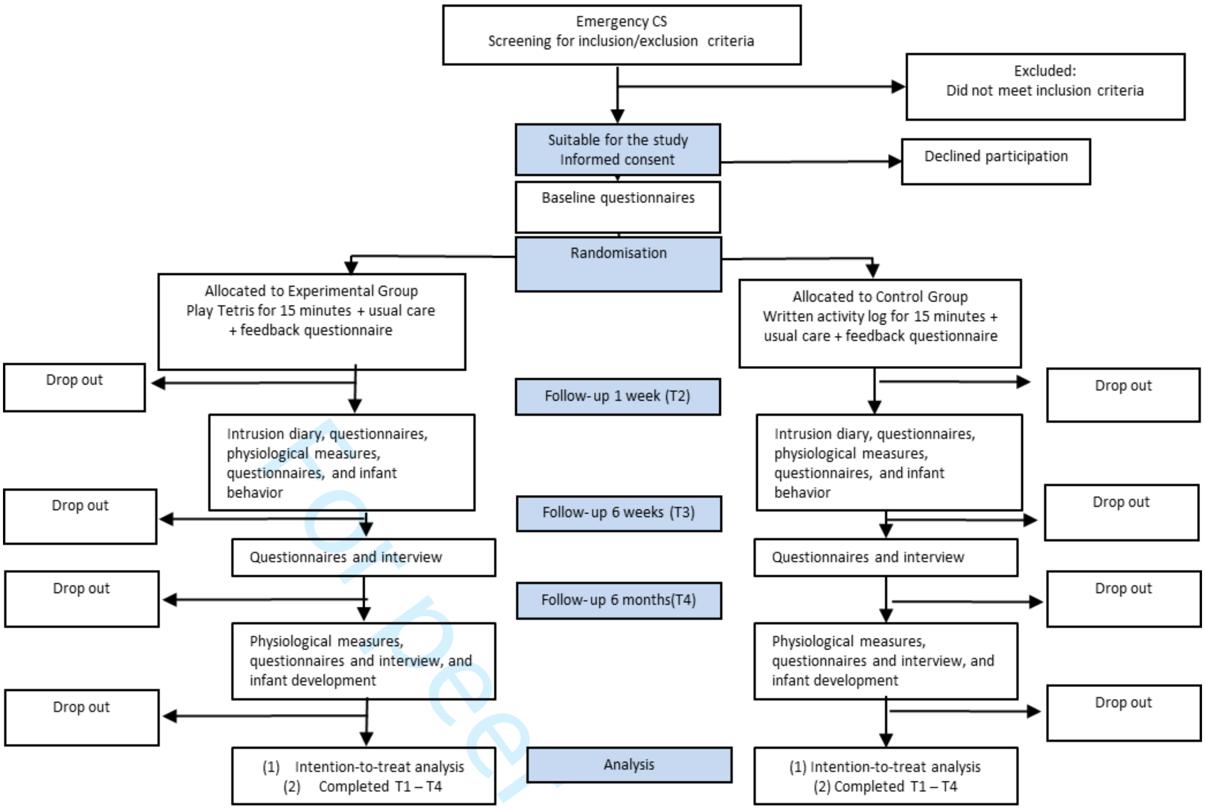

For peer review only

STANDARD PROTOCOL ITEMS: RECOMMENDATIONS FOR INTERVENTIONAL TRIALS

SPIRIT 2013 Checklist: Recommended items to address in a clinical trial protocol and related documents*

Section/item	Item No	Description	Addressed on page number
**Administrative information**			
Title	1	Descriptive title identifying the study design, population, interventions, and, if applicable, trial acronym	1
Trial registration	2a	Trial identifier and registry name. If not yet registered, name of intended registry	6
	2b	All items from the World Health Organization Trial Registration Data Set	n/a
Protocol version	3	Date and version identifier	23
Funding	4	Sources and types of financial, material, and other support	28
Roles and responsibilities	5a	Names, affiliations, and roles of protocol contributors	27
	5b	Name and contact information for the trial sponsor	1
	5c	Role of study sponsor and funders, if any, in study design; collection, management, analysis, and interpretation of data; writing of the report; and the decision to submit the report for publication, including whether they will have ultimate authority over any of these activities	27
	5d	Composition, roles, and responsibilities of the coordinating centre, steering committee, endpoint adjudication committee, data management team, and other individuals or groups overseeing the trial, if applicable (see Item 21a for data monitoring committee)	24
**Introduction**			

For peer review only

Background and rationale	6a	Description of research question and justification for undertaking the trial, including summary of relevant studies (published and unpublished) examining benefits and harms for each intervention	8-14
	6b	Explanation for choice of comparators	16-17
Objectives	7	Specific objectives or hypotheses	14
Trial design	8	Description of trial design including type of trial (eg, parallel group, crossover, factorial, single group), allocation ratio, and framework (eg, superiority, equivalence, noninferiority, exploratory)	14
**Methods: Participants, interventions, and outcomes**			
Study setting	9	Description of study settings (eg, community clinic, academic hospital) and list of countries where data will be collected. Reference to where list of study sites can be obtained	14-15
Eligibility criteria	10	Inclusion and exclusion criteria for participants. If applicable, eligibility criteria for study centres and individuals who will perform the interventions (eg, surgeons, psychotherapists)	14-15
Interventions	11a	Interventions for each group with sufficient detail to allow replication, including how and when they will be administered	16-17
	11b	Criteria for discontinuing or modifying allocated interventions for a given trial participant (eg, drug dose change in response to harms, participant request, or improving/worsening disease)	n/a
	11c	Strategies to improve adherence to intervention protocols, and any procedures for monitoring adherence (eg, drug tablet return, laboratory tests)	14
	11d	Relevant concomitant care and interventions that are permitted or prohibited during the trial	15
Outcomes	12	Primary, secondary, and other outcomes, including the specific measurement variable (eg, systolic blood pressure), analysis metric (eg, change from baseline, final value, time to event), method of aggregation (eg, median, proportion), and time point for each outcome. Explanation of the clinical relevance of chosen efficacy and harm outcomes is strongly recommended	17-22
Participant timeline	13	Time schedule of enrolment, interventions (including any run-ins and washouts), assessments, and visits for participants. A schematic diagram is highly recommended (see Figure)	Table 1 Figure 2

Sample size	14	Estimated number of participants needed to achieve study objectives and how it was determined, including clinical and statistical assumptions supporting any sample size calculations	23
Recruitment	15	Strategies for achieving adequate participant enrolment to reach target sample size	14-15

Methods: Assignment of interventions (for controlled trials)

Allocation:

Sequence generation	16a	Method of generating the allocation sequence (eg, computer-generated random numbers), and list of any factors for stratification. To reduce predictability of a random sequence, details of any planned restriction (eg, blocking) should be provided in a separate document that is unavailable to those who enrol participants or assign interventions	15
Allocation concealment mechanism	16b	Mechanism of implementing the allocation sequence (eg, central telephone; sequentially numbered, opaque, sealed envelopes), describing any steps to conceal the sequence until interventions are assigned	15
Implementation	16c	Who will generate the allocation sequence, who will enrol participants, and who will assign participants to interventions	15-16
Blinding (masking)	17a	Who will be blinded after assignment to interventions (eg, trial participants, care providers, outcome assessors, data analysts), and how	16
	17b	If blinded, circumstances under which unblinding is permissible, and procedure for revealing a participant's allocated intervention during the trial	n/a

Methods: Data collection, management, and analysis

Data collection methods	18a	Plans for assessment and collection of outcome, baseline, and other trial data, including any related processes to promote data quality (eg, duplicate measurements, training of assessors) and a description of study instruments (eg, questionnaires, laboratory tests) along with their reliability and validity, if known. Reference to where data collection forms can be found, if not in the protocol	18-22
	18b	Plans to promote participant retention and complete follow-up, including list of any outcome data to be collected for participants who discontinue or deviate from intervention protocols	n/a

Data management	19	Plans for data entry, coding, security, and storage, including any related processes to promote data quality (eg, double data entry; range checks for data values). Reference to where details of data management procedures can be found, if not in the protocol	22-23
Statistical methods	20a	Statistical methods for analysing primary and secondary outcomes. Reference to where other details of the statistical analysis plan can be found, if not in the protocol	24-25
	20b	Methods for any additional analyses (eg, subgroup and adjusted analyses)	23-24
	20c	Definition of analysis population relating to protocol non-adherence (eg, as randomised analysis), and any statistical methods to handle missing data (eg, multiple imputation)	23-24
**Methods: Monitoring**			
Data monitoring	21a	Composition of data monitoring committee (DMC); summary of its role and reporting structure; statement of whether it is independent from the sponsor and competing interests; and reference to where further details about its charter can be found, if not in the protocol. Alternatively, an explanation of why a DMC is not needed	n/a
	21b	Description of any interim analyses and stopping guidelines, including who will have access to these interim results and make the final decision to terminate the trial	n/a
Harms	22	Plans for collecting, assessing, reporting, and managing solicited and spontaneously reported adverse events and other unintended effects of trial interventions or trial conduct	n/a
Auditing	23	Frequency and procedures for auditing trial conduct, if any, and whether the process will be independent from investigators and the sponsor	n/a
**Ethics and dissemination**			
Research ethics approval	24	Plans for seeking research ethics committee/institutional review board (REC/IRB) approval	24
Protocol amendments	25	Plans for communicating important protocol modifications (eg, changes to eligibility criteria, outcomes, analyses) to relevant parties (eg, investigators, REC/IRBs, trial participants, trial registries, journals, regulators)	23-24

Consent or assent	26a	Who will obtain informed consent or assent from potential trial participants or authorised surrogates, and how (see Item 32)	24
	26b	Additional consent provisions for collection and use of participant data and biological specimens in ancillary studies, if applicable	24
Confidentiality	27	How personal information about potential and enrolled participants will be collected, shared, and maintained in order to protect confidentiality before, during, and after the trial	24
Declaration of interests	28	Financial and other competing interests for principal investigators for the overall trial and each study site	28
Access to data	29	Statement of who will have access to the final trial dataset, and disclosure of contractual agreements that limit such access for investigators	23
Ancillary and post-trial care	30	Provisions, if any, for ancillary and post-trial care, and for compensation to those who suffer harm from trial participation	n/a
Dissemination policy	31a	Plans for investigators and sponsor to communicate trial results to participants, healthcare professionals, the public, and other relevant groups (eg, via publication, reporting in results databases, or other data sharing arrangements), including any publication restrictions	24
	31b	Authorship eligibility guidelines and any intended use of professional writers	n/a
	31c	Plans, if any, for granting public access to the full protocol, participant-level dataset, and statistical code	n/a
**Appendices**			
Informed consent materials	32	Model consent form and other related documentation given to participants and authorised surrogates	upon request
Biological specimens	33	Plans for collection, laboratory evaluation, and storage of biological specimens for genetic or molecular analysis in the current trial and for future use in ancillary studies, if applicable	n/a

*It is strongly recommended that this checklist be read in conjunction with the SPIRIT 2013 Explanation & Elaboration for important clarification on the items. Amendments to the protocol should be tracked and dated. The SPIRIT checklist is copyrighted by the SPIRIT Group under the Creative Commons "Attribution-NonCommercial-NoDerivs 3.0 Unported" license.
